# Exploring the pathways of drug repurposing and *Panax ginseng* treatment mechanisms in chronic heart failure: a disease module analysis perspective

**DOI:** 10.1038/s41598-024-61926-2

**Published:** 2024-05-27

**Authors:** Chengzhi Xie, Ying Zhang, Baochen Zhu, Lin Yang, Jianxun Ren, Na Lang

**Affiliations:** 1 Institute of Basic Medical Sciences, Xiyuan Hospital, China Academy of Chinese Medical Sciences, Beijing, 100091 China; 2 Department of Pharmacy, Dongzhimen Hospital, Beijing University of Chinese Medicine, Beijing, 100700 China; 3 National Clinical Research Center for Chinese Medicine Cardiology, Xiyuan Hospital, China Academy of Chinese Medical Sciences, Beijing, 100091 China; 4 Department of Education, Xiyuan Hospital, China Academy of Chinese Medical Sciences, Beijing, 100091 China

**Keywords:** Drug development, Cardiovascular diseases, Data mining, Predictive medicine, Virtual drug screening

## Abstract

Chronic Heart Failure (CHF) is a significant global public health issue, with high mortality and morbidity rates and associated costs. Disease modules, which are collections of disease-related genes, offer an effective approach to understanding diseases from a biological network perspective. We employed the multi-Steiner tree algorithm within the NeDRex platform to extract CHF disease modules, and subsequently utilized the Trustrank algorithm to rank potential drugs for repurposing. The constructed disease module was then used to investigate the mechanism by which *Panax ginseng* ameliorates CHF. The active constituents of *Panax ginseng* were identified through a comprehensive review of the TCMSP database and relevant literature. The Swiss target prediction database was utilized to determine the action targets of these components. These targets were then cross-referenced with the CHF disease module in the STRING database to establish protein–protein interaction (PPI) relationships. Potential action pathways were uncovered through Gene Ontology (GO) and KEGG pathway enrichment analyses on the DAVID platform. Molecular docking, the determination of the interaction of biological macromolecules with their ligands, and visualization were conducted using Autodock Vina, PLIP, and PyMOL, respectively. The findings suggest that drugs such as dasatinib and mitoxantrone, which have low docking scores with key disease proteins and are reported in the literature as effective against CHF, could be promising. Key components of *Panax ginseng*, including ginsenoside rh4 and ginsenoside rg5, may exert their effects by targeting key proteins such as AKT1, TNF, NFKB1, among others, thereby influencing the PI3K-Akt and calcium signaling pathways. In conclusion, drugs like dasatinib and midostaurin may be suitable for CHF treatment, and *Panax ginseng* could potentially mitigate the progression of CHF through a multi-component-multi-target-multi-pathway approach. Disease module analysis emerges as an effective strategy for exploring drug repurposing and the mechanisms of traditional Chinese medicine in disease treatment.

## Introduction

Chronic heart failure (CHF) is a long-term and progressive clinical syndrome induced by various cardiac disorders, characterized by increased intracardiac pressure and reduced left ventricular ejection fraction^1^. CHF is a major cause of death and disability worldwide, posing a significant public health challenge^2^. Despite considerable advances in the treatment of heart diseases, the prevalence of CHF continues to rise, with only a slight increase in survival rates^3^. A large body of evidence suggests that CHF exhibits significant pathophysiologic heterogeneity. The pathological process of CHF is highly complex, involving pressure or volume overload, systolic and diastolic dysfunction, maladaptive neurohumoral activation, hemodynamic instability, and adverse ventricular remodeling^4^. A combination of cellular and molecular mechanisms contributes to myocardial fibrosis, mitochondrial dysfunction, severe cardiomyocyte apoptosis, myocyte Ca^2+^ cycling impairment or overload, excessive oxidative stress, chronic low-grade inflammation, epigenetic modifications, and noncoding RNAs. Initially, the response to these detrimental molecular mechanisms is adaptive and compensatory, producing beneficial effects for cardiac function in the early stages of CHF. However, when prolonged, this compensatory process ultimately becomes maladaptive or decompensatory, leading to the heart's failure to effectively pump blood throughout the body. Consequently, manipulating these mechanisms is crucial for the pharmacological management of CHF^4,5^. Angiotensin-converting enzyme inhibitors (ACEi) or angiotensin receptor-neprilysin inhibitors (ARNI), beta-blockers (BBs), and mineralocorticoid receptor antagonists (MRAs) form the foundation of therapy for all patients with reduced ejection fraction (HFrEF) in CHF^6^. Over the past decade, treatment options for HFrEF have expanded with the addition of sacubitril/valsartan (ARNi) and ivabradine^7,8^. Recent trial results indicate that alternative drugs may include sodium-glucose cotransporter 2 inhibitors (SGLT2i) such as empagliflozin and dapagliflozin, soluble guanylate cyclase stimulator vericiguat, and cardiac-specific myosin activator omecamtiv mecarbil^9^. However, none of the drugs tested to date have been definitively proven to improve long-term survival in CHF patients, even in those with mild symptoms. Given the complex and dynamic progression of CHF induced by cardiovascular diseases and the increasing clinical interventions for drugs, there is an urgent need to develop new, more effective drugs^10^.

Traditional Chinese medicine has demonstrated therapeutic benefits for CHF in clinical settings. Various Chinese medicinal compounds have been shown to alleviate heart failure symptoms by enhancing myocardial metabolism, promoting angiogenesis and cardiac repair, inhibiting inflammation, and modulating activation of neurohumoral pathways^11–13^. *Panax ginseng* is a quintessential medicinal herb in TCM, characterized predominantly by its bioactive constituents, which are natural saponin compounds. These include ginsenosides Rb1, ginsenosides Rb3, ginsenosides Rg3, among others^14^. These ginsenosides have demonstrated various cardiovascular protective effects, such as anti-atherosclerosis, anti-hypertension, and inhibition of myocardial hypertrophy. When combined with routine treatments like beta-receptor blockers, *Panax ginseng* injections can significantly enhance therapeutic outcomes for patients with heart failure. However, the biological and molecular mechanisms underlying the beneficial effects of *Panax ginseng*'s myriad and complex components remain to be fully elucidated, with the goal of developing effective natural products for CHF at different stages^15–17^.

Drug repurposing, also known as drug repositioning, advocates for the reuse of "old" drugs to treat diseases beyond their original medical indications^18^. This approach has a lower risk of failure and can significantly shorten drug development time and reduce costs, making it an increasingly attractive method of drug development. However, this approach requires the identification of disease modules that play a key role in analyzing pathophysiological processes and potential drug targets of specific diseases, based on protein properties and biomolecular networks^19^. In recent years, research on disease protein interactions has advanced the development of network-based disease molecular mechanisms and analysis methods. These studies have further deduced that disease-associated genes are not randomly distributed within biological networks but instead reside within disease modules^20^. The NeDRex platform^21^, which incorporates sophisticated algorithms for disease modules and drug repositioning, has demonstrated better results in identifying disease modules for various conditions and drug repurposing, such as ovarian cancer and inflammatory bowel disease. This platform can be further exploited to elucidate more disease biological networks and therapeutic mechanisms. In this study, we utilized the CHF disease module for drug repurposing and combined it with network pharmacology approaches to further explore the therapeutic effects and mechanisms of *Panax ginseng* on CHF. This study demonstrates the potential of the disease module approach in understanding diseases and in drug development.

**Figure 1:**
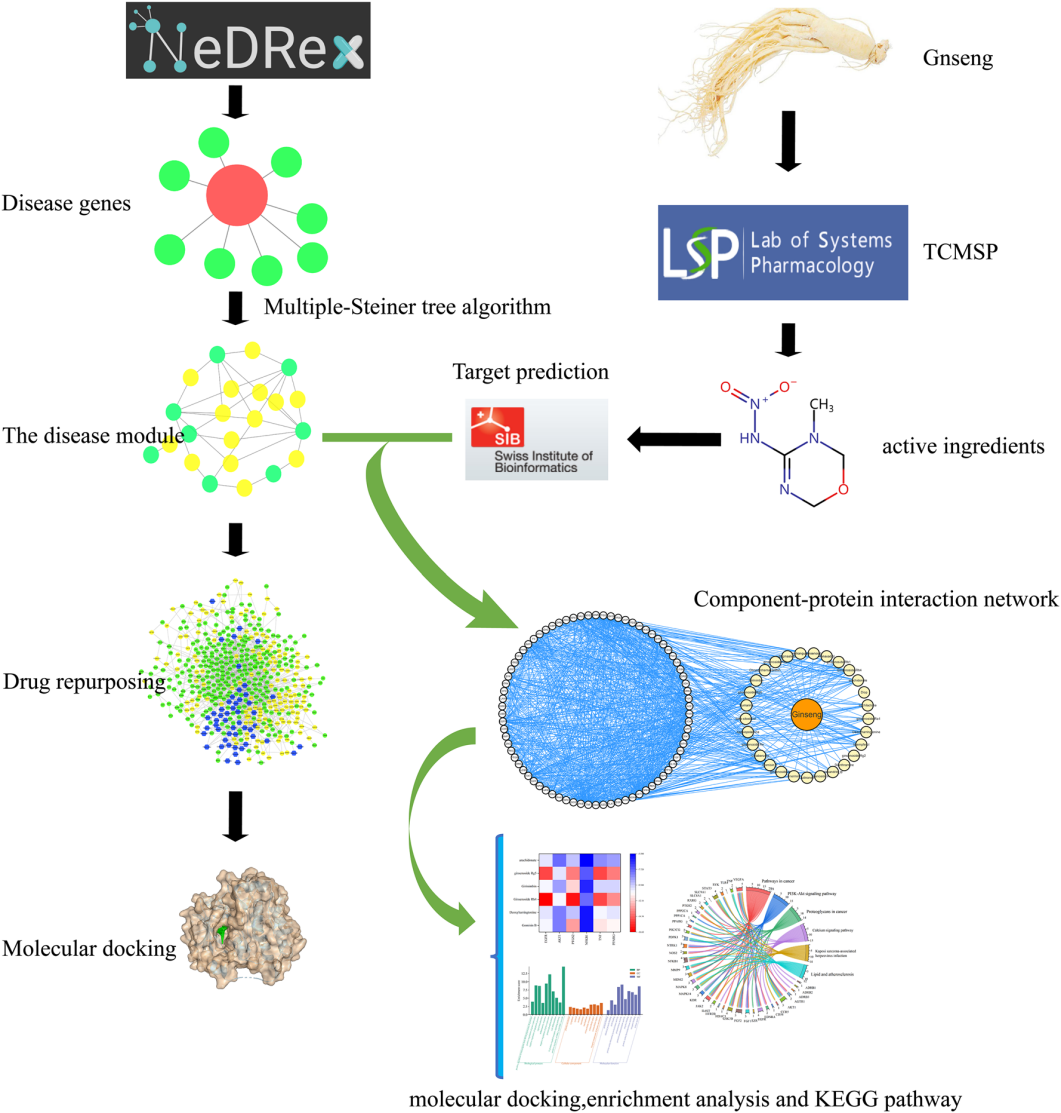
Integrative Framework for Drug Repositioning and Network Pharmacology of *Panax Ginseng* in CHF Management.

The software and databases used for drug repurposing and network pharmacology include: NeDRex (https://nedrex.net/), TCMSP (Traditional Chinese Medicine Systems Pharmacology Database and Analysis Platform, https://old.tcmsp-e.com/tcmsp.php), STRING 12.0, DAVID, Cytoscape 3.9.1, Autodock vina, OpenBabel2.4.1, and PLIP (Protein–Ligand Interaction Profiler, PLIP-Welcome(tu-dresden.de)). The technical process is shown in Fig. 1.

## Methods

### Construction of the CHF disease module

We utilized NeDRex, a web-based and Cytoscape-integrated drug repurposing platform, for its comprehensive capability to integrate disease module detection and drug repurposing algorithms. Through the NeDRex plugin for Cytoscape 3.9.2, we imported networks from NeDRexDB, selecting data options including "Gene-Disorder", "Gene-Protein", and "Protein–Protein". This approach facilitated the construction of gene-disease-pathway networks and protein interaction networks. The "Quick select" function within the NeDRex APP was employed to identify the diseases "congestive heart failure" and "Myocardial ischemia". Subsequently, the "Get Disease Genes" feature enabled the acquisition of seed genes for CHF, grounded in validated disease gene databases. Utilizing the multiple-Steiner tree algorithm, disease modules were mined. In the algorithm's settings, the option "Return Multiple-Steiner tree" was activated with a value of 5, denoting the number of Steiner trees generated by the algorithm. The "Max number of iterations" was set to 5, indicating the algorithm's maximum iteration count. This algorithm sequentially connected genes with seed genes based on protein interaction relations, culminating in the formation of a cohesive disease network. Upon completing these calculations, we established a CHF disease module.

We then analyzed all genes within this disease module for their protein interaction relationships with high confidence (combined score > 0.7) using the String12.0 database, and imported the results into Cytoscape for further analysis. The CentiScaPe 2.2 tool calculated the degree values (Degree unDir), betweenness centrality (Betweenness unDir), and closeness centrality (Closeness unDir) for each node within the network. Targets surpassing the median values for these three parameters were identified as key targets. Among these, the top five targets according to the Degree unDir ranking were selected for molecular docking studies.

### Drug repurposing based on disease module

We applied the TrustRank algorithm, renowned for its ability to rank network credibility, to prioritize candidate drugs by assessing their connectivity within the established disease module. Specifically, the CHF disease module served as the seed node, guiding the algorithm in ranking drugs based on their relevance to the module's network. The ranking process culminated in the selection of the top 30 drugs, which were deemed most closely connected to the CHF disease module. These selected drugs were subsequently subjected to molecular docking analysis to assess their potential efficacy in treating congestive heart failure.

### Selection of *Panax ginseng* targets

We engaged the Traditional Chinese Medicine Systems Pharmacology Database and Analysis Platform (TCMSP) for the identification of active components in *Panax ginseng*. The selection criteria focused on active components with oral bioavailability (OB) values exceeding 30% and drug-likeness (DL) values above 0.18^22^. This screening was complemented by a thorough review of the existing literature to enrich the dataset with validated active components. To predict the targets of these active components, the Swiss Target Prediction platform was utilized. After eliminating predicted duplicate entries, the resulting targets were designated as the active targets for *Panax ginseng*. This methodological approach ensured a comprehensive and scientifically grounded selection of active components and their corresponding targets, pivotal for the subsequent stages of the research.

### Identification and analysis of key components and targets

The expanded disease module genes, derived from NeDRex, were identified as CHF-correlated genes. Utilizing Venny 2.1.0, we performed an intersection between these genes and the active targets of *Panax ginseng*, pinpointing the shared targets. This intersection facilitated the construction of a "component-disease target" network, employing Cytoscape to visualize the interactions pertinent to CHF treatment within traditional Chinese medicine paradigms. The CentiScaPe 2.2 tool within Cytoscape was instrumental in calculating the degree, betweenness, and closeness centrality metrics for each component. Components exceeding the median values across these three metrics were designated as key components for the compound treatment of CHF.

Concurrently, we analyzed the shared targets in the STRING database, specifying "Homo sapiens" as the species. This analysis aimed to elucidate protein interaction relationships with medium confidence (combined score > 0.4), excluding solitary targets lacking interactions. The resultant Protein–Protein Interaction (PPI) network was then imported into Cytoscape 3.9.2, where the CentiScaPe 2.2 plugin facilitated topological analysis and visualization, identifying the paramount targets for *Panax ginseng* in the amelioration of CHF.

### Gene ontology and pathway enrichment analysis

We entered the intersecting targets between *Panax ginseng* and the CHF disease module into DAVID, selecting the full human gene set as the background, to conduct Gene Ontology (GO) analysis and Kyoto Encyclopedia of Genes and Genomes (KEGG) pathway enrichment analysis^23–25^. The focus was on "Homo sapiens" to ensure species-specific relevance. Criteria for significance were set with a P Value threshold of < 0.05 to filter the results. This rigorous selection process facilitated the identification of the most pertinent GO terms and KEGG pathways, which were then meticulously ranked by their Count value. The enrichment results underwent visualization through an online data analysis and visualization platform, https://www.bioinformatics.com.cn, enhancing the interpretability of the complex data and providing a clear, graphical representation of the findings.

### Molecular docking studies

We carried out molecular docking between the top 30 candidate drugs derived from our TrustRank analysis and the five key targets within the CHF disease module. Initially, three-dimensional (3D) structural files of protein targets were obtained in PDB format from the Protein Data Bank (PDB) database, while the structural files for key components were sourced from the PubChem database and converted into PDB format using OpenBabel 2.4.1. Prior to docking, protein targets underwent preparation processes including dehydration and the addition of hydrogen atoms using AutoDockTools 1.5.6, which also facilitated the setting of appropriate active sites and docking parameters.

Subsequently, molecular docking simulations were conducted using AutoDock Vina, aiming to identify the most favorable interactions. For a more detailed examination of these interactions, the Protein–Ligand Interaction Profiler (PLIP) was employed, enabling an exploration of the forces at play between the molecules and the proteins. Visualization of these intricate interactions was achieved using PyMOL software, providing a graphical representation that underscored the potential of these compounds in the context of therapeutic interventions for CHF. The five key components and five key proteins in *panax ginseng* improving CHF were also subjected to the above operations to further determine the therapeutic effect of *panax ginseng*.

## Results

### Construction of CHF disease module

**Figure 2:**
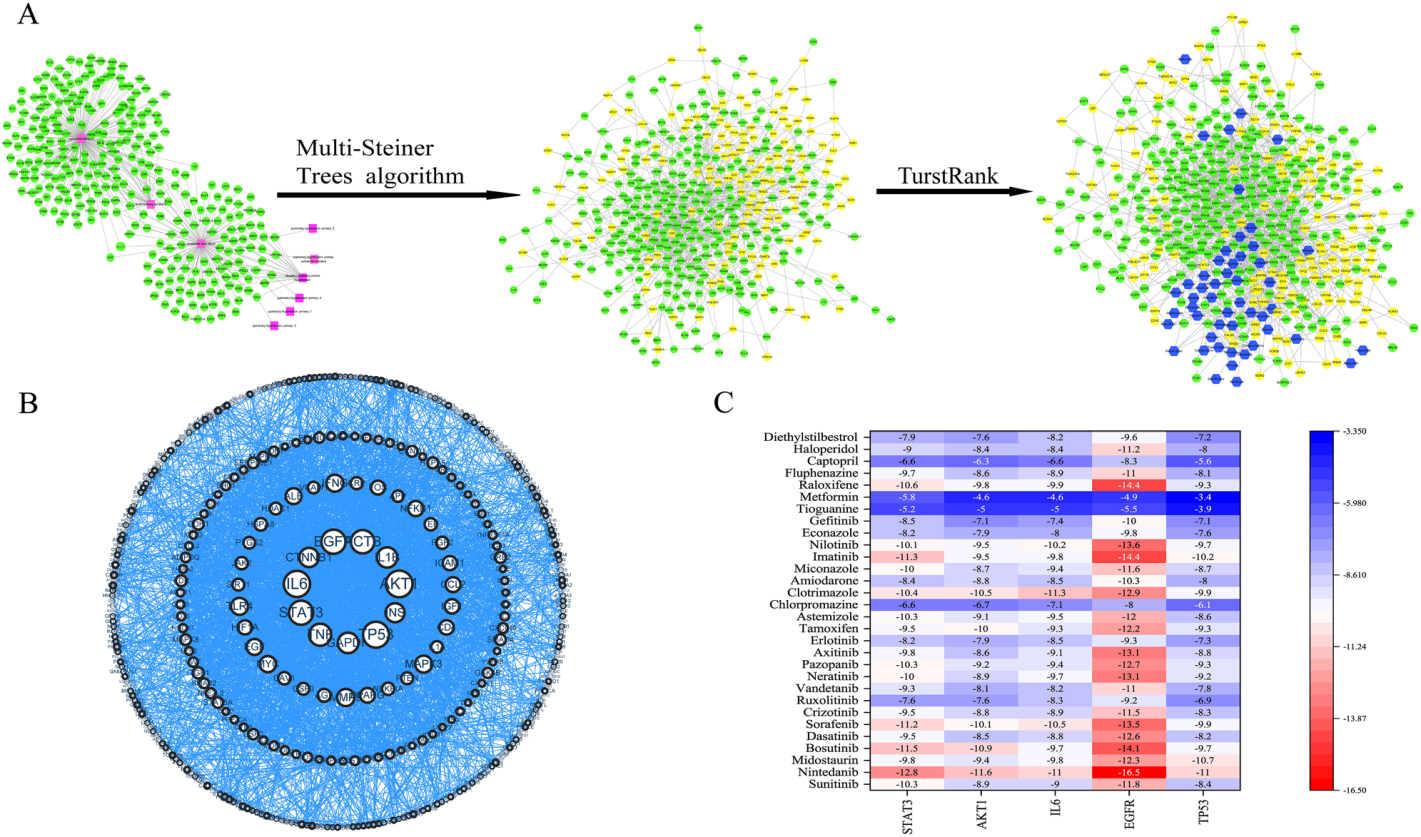
Comprehensive Analysis of CHF Disease Module: (**A**) CHF Module Construction with Seed and Connecting Gene Analysis for Drug Repurposing, Green represents seed genes, i.e., verified disease genes. Yellow is the connecting gene, used to connect seed disease genes to form a disease module. Blue is the candidate drug, (**B**) CHF Protein Interaction Network, (**C**) Top Protein-Drug Docking Evaluations in CHF Module.

From NeDRexDB, 296 CHF seed genes were collected. After calculating with the multiple-Steiner tree algorithm, a total of 445 targets were formed in the CHF disease module. Figure 2A shows the process of establishing the disease module of CHF. Green nodes are CHF seed genes obtained from NeDRexDB, and yellow nodes are connecting genes added after calculation by the multiple-Steiner tree algorithm, which are used to connect seed genes to form the smallest subnetwork. The algorithm integrates the two into the disease module of CHF.

The protein interaction relationship in the disease module was explored using string12.0. The results were imported into Cytoscape software for analysis and visualization, as shown in Fig. 2B. The top five targets selected by the Centiccape2.2 tool are signal transducer and activator of transcription 3(STAT3), interleukin 6 (IL-6), tumor protein p53 (TP53), AKT serine/threonine kinase 1 (AKT1), epidermal growth factor receptor (EGFR).

### Drug reuse of CHF

Based on TurstRank, 50 subsequent drugs for CHF were co-ranked, including Sunitinib, Nintedanib, Midostaurin, Bosutinib, Dasatinib, Sorafenib, Crizotinib, etc., all of which have been approved by the FDA. The top 30 drugs are shown in Table 1.

**Table 1:** TrustRank algorithm-driven CHF drug repurposing rankings.

Rank	Drug	Drugbank ID
1	Sunitinib	DB01268
2	Nintedanib	DB09079
3	Midostaurin	DB06595
4	Bosutinib	DB06616
5	Dasatinib	DB01254
6	Sorafenib	DB00398
7	Crizotinib	DB08865
8	Ruxolitinib	DB08877
9	Vandetanib	DB05294
10	Neratinib	DB11828
11	Pazopanib	DB06589
12	Axitinib	DB06626
13	Erlotinib	DB00530
14	Tamoxifen	DB00675
15	Astemizole	DB00637
16	Chlorpromazine	DB00477
17	Clotrimazole	DB00257
18	Amiodarone	DB01118
19	Miconazole	DB01110
20	Imatinib	DB00619
21	Nilotinib	DB04868
22	Econazole	DB01127
23	Gefitinib	DB00317
24	Tioguanine	DB00352
25	Metformin	DB00331
26	Raloxifene	DB00481
27	Fluphenazine	DB00623
28	Captopril	DB01197
29	Haloperidol	DB00502
30	Diethylstilbestrol	DB00255

It is worth noting that the results of the algorithm ranking can only represent that the drug has a direct connection with the disease module, and the closeness of the connection increases with the ranking, but it cannot determine whether the drug is beneficial to the disease. The specific effect still needs to be determined in combination with previous research and animal experiments. For example, Sunitinib, ranked first, has been shown in clinical trials to potentially cause strong cardiovascular side effects, including causing cardiac toxicity, cardiac dysfunction, and promoting the development of hypertension^26,27^. This result indicates that the drugs screened through the disease module have a close relationship with the disease, but it is still necessary to determine whether it is beneficial. Nintedanib, ranked second, is a tyrosine kinase inhibitor approved for idiopathic pulmonary fibrosis (IPF) and chronic fibrosing interstitial lung disease (ILD)^28^. Current animal experiments have shown that it has a good effect on myocardial fibrosis in CHF, which may be related to reducing the expression of collagen genes (collagen type I alpha 1 chain, collagen type III alpha 1 chain), and its beneficial effect on the cardiac function of mice undergoing transverse aortic constriction (TAC) surgery remains unchanged even after treatment is interrupted^29^. The authors in the literature have encouraged further verification of its effect on CHF through large animal model experiments. The study of Midostaurin, ranked third, also shows that it can up-regulate the expression of endothelia NO synthase(eNOS) gene in mouse microcirculation and protect eNOS function, thus possibly playing a role in vascular protection and anti-atherosclerosis^30^.

### Analysis of *Panax ginseng*'s mechanism in improving CHF

**Figure 3:**
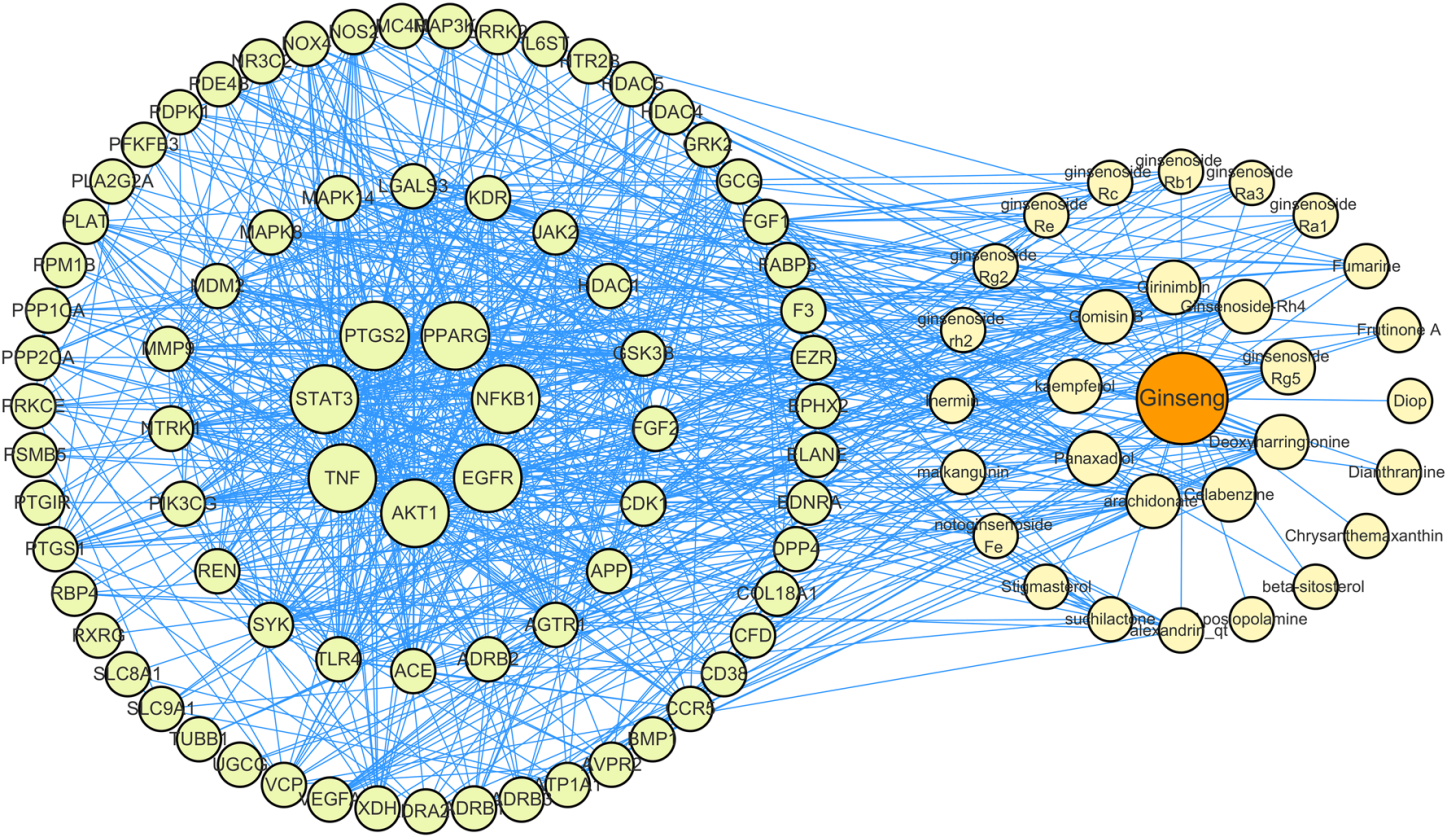
Mapping the *Panax ginseng* Interaction Network with Components and Proteins. The orange node is *Panax ginseng*, the yellow node is the component in *Panax ginseng*, and the green node is the target protein.

**Figure 4:**
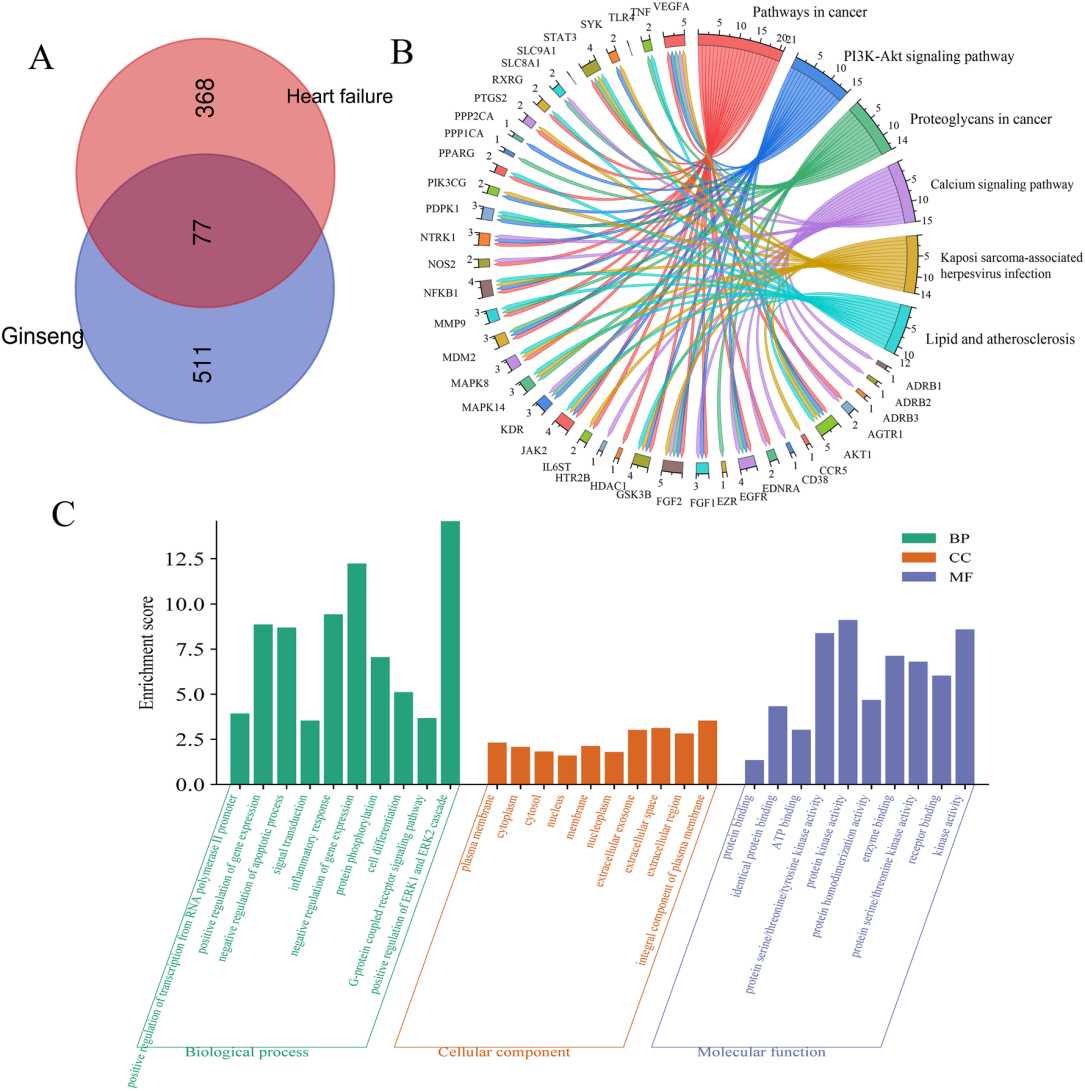
Integrated Analysis of Traditional Chinese Medicine and CHF Targets: (**A**) Venn Diagram of Overlapping Targets. (**B**) KEGG Pathway Enrichment Chord Diagram. (**C**) GO Enrichment Analysis.

Through TCMSP, 22 components of *Panax ginseng* were collected, and 7 were added through literature review, totaling 29 active ingredients. Using Swiss target prediction, 588 unique targets were predicted for the active ingredients. The intersection of the active ingredient targets and the CHF disease module targets resulted in 77 intersecting targets, venn diagram is shown in Fig. 4A. The protein interaction relationship was explored using string12.0 and exported. The *Panax ginseng* bioactive ingredients, related target information, and protein interaction relationships were imported into Cytoscape 3.9.2 software to construct a component-protein interaction network, as shown in Fig. 3. It contains 108 nodes and 833 edges. The top six key components and targets were selected based on degree, centrality, and closeness centrality. The top six key components for treatment are Gomisin B, Deoxyharringtonine, ginsenoside rh4, Girinimbin, ginsenoside rg5, arachidonate. The top six targets are AKT1, tumor necrosis factor (TNF), nuclear factor kappa B subunit 1(NFKB1), peroxisome proliferator activated receptor gamma (PPARG), EGFR, prostaglandin-endoperoxide synthase 2 (PTGS2).

### Key gene screening and enrichment analysis

The DAVID database was used to perform GO and KEGG analysis on the 77 intersecting targets. With *P* < 0.01 as the screening condition, 306 biological process (BP) entries were obtained, mainly related to positive regulation of transcription from RNA polymerase II promoter, positive regulation of gene expression, negative regulation of apoptotic process, signal transduction, inflammatory response, negative regulation of gene expression, etc. 46 cellular component (CC) entries were obtained, including plasma membrane, cytoplasm, cytosol, nucleus, membrane, nucleoplasm, extracellular exosome. 54 molecular function (MF) entries were obtained, mainly involving protein binding, identical protein binding, ATP binding, protein serine/threonine/tyrosine kinase activity, protein kinase activity, protein homodimerization activity, enzyme binding. The results of the GO analysis are depicted as a bar graph in Fig. 4C. KEGG pathway enrichment with *P* < 0.05 as the screening condition resulted in 110 pathways. The enrichment analysis results show that the signaling pathways closely related to CHF include: PI3K-Akt signaling pathway, Calcium signaling pathway, Lipid and atherosclerosis, AGE-RAGE signaling pathway in diabetic complications, etc. The top six pathways were plotted as a chord diagram, as shown in Fig. 4B.

### Molecular docking

The top thirty candidate drugs were docked with key proteins in the disease module. The results are shown in the heatmap part of Fig. 2C. Except for Dasatinib and STAT3, which could not predict binding, the highest docking scores of metformin and TP53 protein was − 3.4 kcal/mol. The lowest docking scores of Nintedanib and EGFR was − 16.5. The docking scores of most drugs and proteins is below − 7 kcal/mol, indicating that the drugs have good binding activity with the proteins.

**Figure 5:**
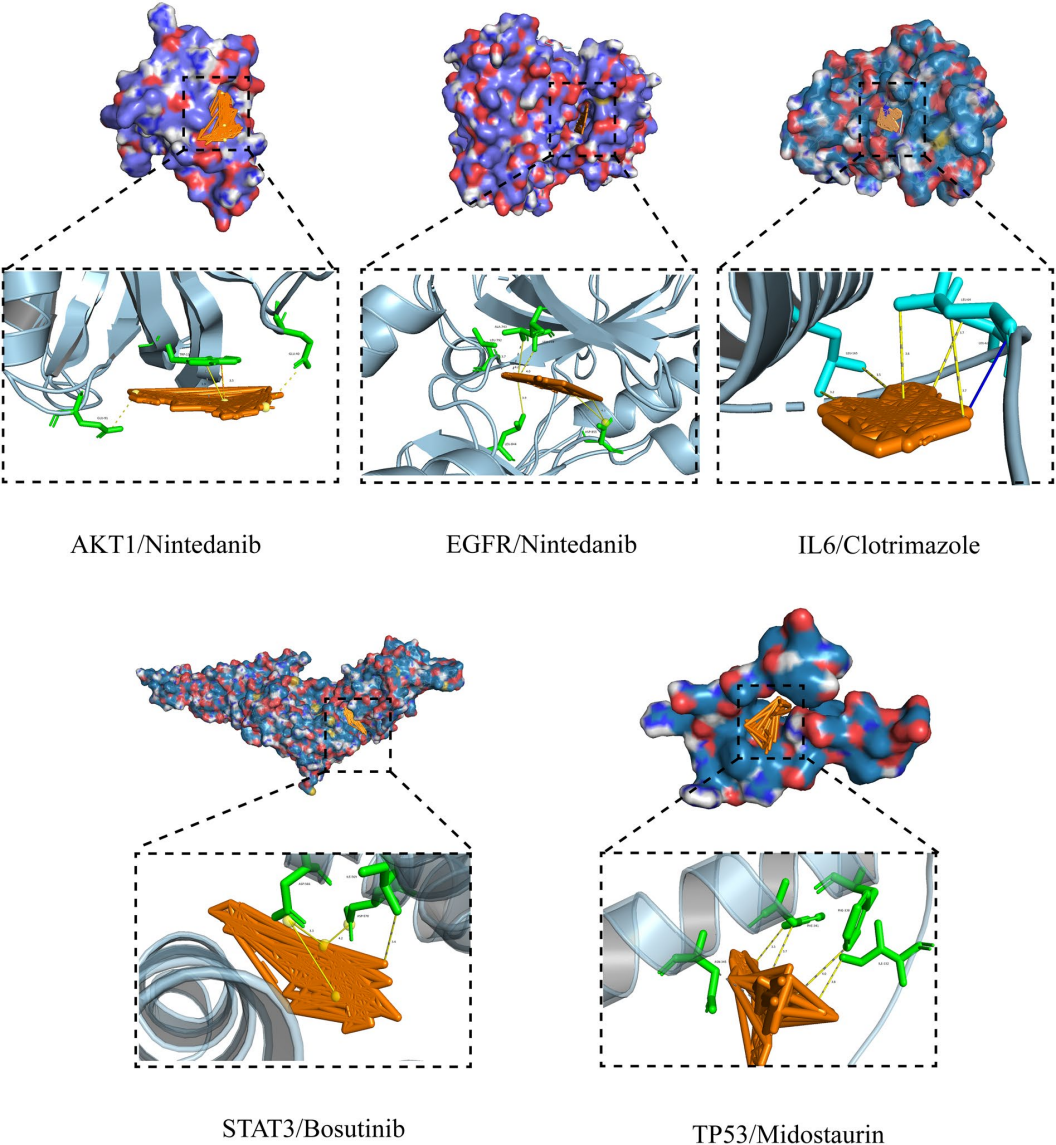
Drug Repurposing Insights: Molecular Docking of Repurposed Drugs with Key Protein Targets.

PLIP was used to explore the forces between molecules and proteins, and the results were visualized using PyMOL software, as shown in Fig. 5. Nintedanib has low docking scores with each protein, with hydrogen bonding with AKT1 and salt bridge and hydrophobic interactions with EGFR. IL-6 and Clotrimazole are connected by hydrogen bonds and hydrophobic interactions. In summary, each drug binds to key proteins in different ways, showing low docking scores.

The molecular docking results of the key components in *Panax ginseng* and the key targets show that ginsenoside rh4 and ginsenoside rg5 have extremely low docking scores with each key target. The molecular docking results are shown in Table 2. Previous research has shown that ginsenoside components play an important role in the treatment of CHF, so ginsenoside rh4 and ginsenoside rg5 may play an important role in treatment, and further exploration can be combined with experiments in the future.

**Table 2:** Binding affinity scores (kcal/mol) between *Panax Ginseng* components and key protein targets.

	EGFR	AKT1	PTGS2	NFKB1	TNF	PPARG
Gomisin B	− 10.9	− 8.3	− 13	− 6.1	− 11.5	− 11.2
Deoxyharringtonine	− 10.5	− 8.4	− 9.9	− 6	− 10.7	− 10.6
Ginsenoside rh4	− 16.6	− 10.7	− 16.1	− 7.9	− 15.1	− 13.6
Girinimbin	− 10.8	− 9	− 12.1	− 6.3	− 10	− 10.2
Ginsenoside rg5	− 15	− 10.4	− 13.8	− 7.6	− 15	− 13.7
arachidonate	− 10.5	− 7.9	− 9.8	− 5.5	− 8.6	− 9

## Discussion

Increasing evidences show that special proteins related to diseases do not exist in isolation, but exist within disease modules^31^. The diversity of pathological characteristics and clinical manifestations of diseases may stem from the disruption of disease modules, reflecting the imbalance in interactions and regulatory mechanisms across different levels of biological systems during the disease process. For most diseases, the related disease modules are not still complete known, so network analysis methods based on genome-wide disease gene associations and high-throughput interactions are effective ways to explore disease modules^32,33^. CHF is considered a global disease affecting about 1% to 2% of adults, with various, parallel developing clinical signs and symptoms. The important treatment strategies are the prevention of worsening chronic heart failure in patients who are prone to decompensation. Continuously optimized drug treatments and fine-tuning of these processes based on evidence-based medicine are all trying to keep these compensatory mechanisms of CHF within a reasonable physiological range. New drugs may bring more feasibility to the treatment of CHF, and drug repurposing is an economically reasonable choice. In this study, we highlight the utility of the disease module approach in elucidating disease mechanisms by exploring drug repurposing for CHF and assessing the therapeutic potential of *panax ginseng* against CHF.

Utilizing the NeDRex platform, which amalgamates biomedical databases with sophisticated network algorithms, we have developed a disease module for CHF. This module was constructed by identifying seed genes associated with CHF, then discovering genes connected to these seeds and the pathways linking them through protein interaction networks. These components were subsequently integrated to form a comprehensive disease network. Through the analysis of the disease module, we found a variety of drugs that may be applicable to CHF, including Sunitinib, Nintedanib, Midostaurin, Bosutinib, Dasatinib, etc. Sunitinib has the closest relationship with the disease module and the highest Trustrank ranking result, but clinical results show that it has strong cardiotoxicity. Therefore, the drugs mined based on disease modules have a close relationship with the disease, but the benefits need to be evaluated in combination with animal experiments and clinical experiments. Previous studies have shown that Midostaurin can up-regulate the expression of eNOS genes in mouse microcirculation and maintian eNOS function, which may have vascular protective and anti-atherosclerotic effects by attenuating oxidized stress. The results of molecular docking show that Nintedanib has extremely strong binding activity with key proteins. As a tyrosine kinase inhibitor used for idiopathic pulmonary fibrosis (IPF) and chronic fibrotic interstitial lung disease (ILD), studies have shown that it can well inhibit myocardial fibrosis in the CHF process of mice in the transverse aortic constriction (TAC) surgery model, and its beneficial effects on cardiac function continue even after the end of treatment. Therefore, the effectiveness of Nintedanib in treating CHF can be further verified in the future in combination with large samples clinical study or animal experiments. Dasatinib combined with quercetin can effectively eliminate senescent cells in myocardial tissue, can alleviate the progression of myocardial injury, and can be used for age-related cardiac pathology^34^. In summary, candidate drugs such as Midostaurin, Nintedanib, and Dasatinib screened out through drug repurposing of disease modules can be verified for their effectiveness against CHF through animal experiments or clinical research in the future.

TCM interventions the progression of diseases with a multi-component, multi-target mechanism. Network pharmacology and integrative pharmacology have attempted to understand TCM treatment in a more holistic and systematic way. Better understanding of diseases is required to better explain the therapeutic mechanisms of TCM. Disease modules may provide a better explanation for TCM treatment. Previous studies have shown that *Panax ginseng* has a good therapeutic effect on CHF. Systematic pharmacology analysis shows that the key bioactive components are Gomisin B, Deoxyharringtonine, ginsenoside rh4, Girinimbin, ginsenoside rg5, and arachidonate. ginsenosides are the main effective components in *Panax ginseng* and play an important role in the treatment of cardiovascular diseases^35^. ginsenoside rg5 and Rh4, as important bioactive components found in *Panax ginseng*, have shown certain benefits for CHF in this study. Previous research, such as the work by Yang et al.^36^, has revealed the role of ginsenoside rg5 in enhancing the resistance of cardiomyocytes to ischemic injury. This effect is mainly achieved by regulating two key enzymes, hexokinase II (HK-II) and dynamin-related protein 1 (Drp1). Specifically, ginsenoside rg5 can protect HK-II in mitochondria by counteracting fatty acid oxidation and preventing cellular oxidation. In a mouse model of myocardial ischemia induced by isoproterenol, it increases the binding of HK-II and reduces the recruitment of Drp1 to mitochondria, thereby preventing cell apoptosis. Furthermore, ginsenoside rg5 regulates angiogenesis and vasodilation by activating the IGF-1 receptor (IGF-1R), with its angiogenic activity closely related to the specific increase in IGF-1R phosphorylation and the subsequent activation of multiple angiogenic signals^37^. At the same time, studies on ginsenoside rh4 have also demonstrated its significant anti-inflammatory activity. Hu et al. found through RT-PCR, Western blot, and ELISA analyses that ginsenoside rh4 significantly inhibited the production of pro-inflammatory cytokines (such as TNF-α, IL-6, and IL-1β) and inflammation-related enzymes induced by lipopolysaccharide (LPS)^38^. Additionally, ginsenoside rh4 can interfere with NFIL3, inhibiting myocardial remodeling induced by Ang II^39^. Considering the analysis results of this study, we believe that ginsenoside rg5 and ginsenoside rh4 have significant benefits for cardiovascular health, although the underlying mechanisms still require further exploration. The top six targets are AKT1, TNF, NFKB1, PPARG, EGFR, PTGS2. The AKT coenzyme series plays an extremely important role in the cardiovascular system, with its effects on downstream targets determining its functions in cardiovascular processes, including cell survival, growth, proliferation, angiogenesis, vascular relaxation, and cell metabolism. AKT1 is also one of the therapeutic targets of the key component ginsenoside rg5. A clinical study has shown that a reduction in NFKB1 activity may play a regulatory role in the onset of CHF, potentially increasing the severity of the disease by promoting cardiac remodeling and functional deterioration^40^. The intervention of the PPARG/LXRα pathway can alleviate cholesterol accumulation induced by atherosclerosis^41^. EGFR is a receptor tyrosine kinase^42^, including EGFR (ErbB-1, HER1), HER2 (ErbB2/neu), etc. Studies have shown that ErbB2-4 has potential roles in regulating cardiomyocyte proliferation and cardiotoxicity related to cancer treatment. Endogenous ligands bind to EGFR to induce dimerization or stabilization of existing dimers, and participate in various signal transduction pathways in myocardial tissue by trans-phosphorylating the C-terminal tyrosine residues through the kinase structure domain. In essence, the treatment of Chronic Heart Failure (CHF) with *Panax ginseng* employs a comprehensive approach that involves multiple targets, pathways, and components. Key constituents, namely ginsenoside rh4 and ginsenoside rg5, potentially exert their medicinal effects by specifically targeting EGFR, PTGS2, TNF among others. This, in turn, influences a variety of pathways such as the PI3K-Akt signaling pathway, Calcium signaling pathway, and Lipid and atherosclerosis, thereby contributing to their therapeutic efficacy. Combined with the disease module. It may further explain the complex mechanisms of traditional Chinese medicine treatment for CHF.

Disease module analysis, as a network-based research method, provides a new way to comprehensively explore disease mechanisms and develop potential therapeutic drugs in the present study. This approach may uncover the intricacies of disease onset and progression, along with its interplay with various biological processes, by mapping out a network comprising disease-associated genes and proteins. It is noteworthy that analyzing disease modules holds substantial importance in deciphering the complex mechanisms behind TCM treatments, offering profound insights into their efficacy. For example, the improvement of CHF by *Panax ginseng* involves multiple components affecting differently pathological targets. We can understand the multi-component-multi-target action of TCM more deeply through disease module analysis, that provide a new perspective and path for TCM research and application. In general, disease module analysis provides valuable information for disease research and drug development, helping to optimize treatment strategies for various diseases. Nonetheless, it's essential to acknowledge the limitations inherent in disease module analysis and the necessity to supplement it with a variety of other research methodologies. This will guarantee a more thorough and scientifically robust assessment of potential treatment strategies. In future studies, integrating diverse data sources and methodologies, as well as a more exhaustive analysis of protein interaction networks, will be crucial. Such efforts are expected to enhance the precision and applicability of disease module studies, paving the way for more innovative breakthroughs in disease treatment (Supplementary Information file 1).

### Supplementary Information

Supplementary Tables.

## Data Availability

All data generated or analysed during this study are included in this published article and its supplementary information files.
