# Supplementary material for: Exploring the pathways of drug repurposing and Panax ginseng treatment mechanisms in chronic heart failure: a disease module analysis perspective
Source: Sci Rep. 2024 May 27;14:12109. doi: 10.1038/s41598-024-61926-2 (PMC11130340; doi:10.1038/s41598-024-61926-2)
Supplement: Supplementary file 1 — Supplementary Tables. [file 41598_2024_61926_MOESM1_ESM.pdf]

Table1 Genes in the disease module.

The 'True' in the 'seed' column represents its seed gene, otherwise it is the connection gene calculated by the algorithm.

displayName	name	participation_number	seed	shared name	type	YES
CXCL10	entrez.3627	5	TRUE	entrez.3627	Gene	1
COX5B	entrez.1329	5	TRUE	entrez.1329	Gene	1
KCNA5	entrez.3741	5	TRUE	entrez.3741	Gene	1
CSF3R	entrez.1441	2		entrez.1441	Gene	0
PTX3	entrez.5806	1		entrez.5806	Gene	0
MAP3K8	entrez.1326	5	TRUE	entrez.1326	Gene	1
TUBB1	entrez.81027	5	TRUE	entrez.81027	Gene	1
GDF2	entrez.2658	5	TRUE	entrez.2658	Gene	1
UBQLN1	entrez.29979	1		entrez.29979	Gene	0
MSTN	entrez.2660	5	TRUE	entrez.2660	Gene	1
GCG	entrez.2641	5	TRUE	entrez.2641	Gene	1
CS	entrez.1431	5	TRUE	entrez.1431	Gene	1
MAPK14	entrez.1432	5	TRUE	entrez.1432	Gene	1
GRB2	entrez.2885	1		entrez.2885	Gene	0
GCH1	entrez.2643	5	TRUE	entrez.2643	Gene	1
CFD	entrez.1675	5	TRUE	entrez.1675	Gene	1
GNA12	entrez.2768	5	TRUE	entrez.2768	Gene	1
CSF2	entrez.1437	5	TRUE	entrez.1437	Gene	1
NKIRAS1	entrez.28512	1		entrez.28512	Gene	0
ELOVL6	entrez.79071	5	TRUE	entrez.79071	Gene	1
CSF3	entrez.1440	5	TRUE	entrez.1440	Gene	1

GHRL	entrez.51738	5	TRUE	entrez.51738	Gene	1
GPX4	entrez.2879	5	TRUE	entrez.2879	Gene	1
LGALS9	entrez.3965	1		entrez.3965	Gene	0
GYG1	entrez.2992	5	TRUE	entrez.2992	Gene	1
GBP2	entrez.2634	5	TRUE	entrez.2634	Gene	1
JUNB	entrez.3726	5	TRUE	entrez.3726	Gene	1
TLN1	entrez.7094	1		entrez.7094	Gene	0
RHOU	entrez.58480	5		entrez.58480	Gene	0
TLE4	entrez.7091	5	TRUE	entrez.7091	Gene	1
CIAO1	entrez.9391	1		entrez.9391	Gene	0
TLR4	entrez.7099	5	TRUE	entrez.7099	Gene	1
GATM	entrez.2628	5	TRUE	entrez.2628	Gene	1
LEP	entrez.3952	1		entrez.3952	Gene	0
CRYAB	entrez.1410	1		entrez.1410	Gene	0
FBXO2	entrez.26232	1		entrez.26232	Gene	0
DDX5	entrez.1655	1		entrez.1655	Gene	0
JAK2	entrez.3717	5	TRUE	entrez.3717	Gene	1
LGALS3	entrez.3958	2		entrez.3958	Gene	0
CYBB	entrez.1536	5	TRUE	entrez.1536	Gene	1
SOCS3	entrez.9021	5	TRUE	entrez.9021	Gene	1
NOX4	entrez.50507	5	TRUE	entrez.50507	Gene	1
LDHB	entrez.3945	5	TRUE	entrez.3945	Gene	1
CRP	entrez.1401	5	TRUE	entrez.1401	Gene	1
RAB12	entrez.201475	5	TRUE	entrez.201475	Gene	1
NTRK1	entrez.4914	1		entrez.4914	Gene	0
ADIPOQ	entrez.9370	5	TRUE	entrez.9370	Gene	1
TBX4	entrez.9496	5	TRUE	entrez.9496	Gene	1
ACE	entrez.1636	5	TRUE	entrez.1636	Gene	1
GCLC	entrez.2729	5	TRUE	entrez.2729	Gene	1
ELANE	entrez.1991	3		entrez.1991	Gene	0
ELAVL1	entrez.1994	5		entrez.1994	Gene	0
E2F4	entrez.1874	1		entrez.1874	Gene	0
DCN	entrez.1634	5	TRUE	entrez.1634	Gene	1

GPR21	entrez.2844	1		entrez.2844	Gene	0
MAPRE1	entrez.22919	5	TRUE	entrez.22919	Gene	1
GSTP1	entrez.2950	5	TRUE	entrez.2950	Gene	1
GK	entrez.2710	5	TRUE	entrez.2710	Gene	1
SCNM1	entrez.79005	1		entrez.79005	Gene	0
TPM4	entrez.7171	5	TRUE	entrez.7171	Gene	1
LSM3	entrez.27258	1		entrez.27258	Gene	0
TXNRD1	entrez.7296	5	TRUE	entrez.7296	Gene	1
GSTM2	entrez.2946	5	TRUE	entrez.2946	Gene	1
FBXO6	entrez.26270	2		entrez.26270	Gene	0
GSTM3	entrez.2947	1		entrez.2947	Gene	0
DLAT	entrez.1737	5	TRUE	entrez.1737	Gene	1
CCR10	entrez.2826	1		entrez.2826	Gene	0
GSTM4	entrez.2948	1		entrez.2948	Gene	0
DLG1	entrez.1739	1		entrez.1739	Gene	0
ACVRL1	entrez.94	5	TRUE	entrez.94	Gene	1
CTTNBP2	entrez.83992	5	TRUE	entrez.83992	Gene	1
VAPA	entrez.9218	3		entrez.9218	Gene	0
GSTM1	entrez.2944	1		entrez.2944	Gene	0
RALBP1	entrez.10928	5	TRUE	entrez.10928	Gene	1
PRDX6	entrez.9588	5	TRUE	entrez.9588	Gene	1
GSN	entrez.2934	5	TRUE	entrez.2934	Gene	1
DUSP6	entrez.1848	5	TRUE	entrez.1848	Gene	1
GSTA1	entrez.2938	5	TRUE	entrez.2938	Gene	1
H4C15	entrez.554313	1		entrez.554313	Gene	0
DTX1	entrez.1840	1		entrez.1840	Gene	0
DUSP1	entrez.1843	5	TRUE	entrez.1843	Gene	1
GSK3B	entrez.2932	5	TRUE	entrez.2932	Gene	1
AGTR1	entrez.185	5	TRUE	entrez.185	Gene	1
EIF2AK3	entrez.9451	5	TRUE	entrez.9451	Gene	1
AGT	entrez.183	5	TRUE	entrez.183	Gene	1
FAM241A	entrez.132720	2		entrez.132720	Gene	0
TP53	entrez.7157	5		entrez.7157	Gene	0

TFRC	entrez.7037	5	TRUE	entrez.7037	Gene	1
PDIA3	entrez.2923	5	TRUE	entrez.2923	Gene	1
EGFR	entrez.1956	2		entrez.1956	Gene	0
EGR2	entrez.1959	5	TRUE	entrez.1959	Gene	1
ACTG1	entrez.71	5	TRUE	entrez.71	Gene	1
TNNT2	entrez.7139	5	TRUE	entrez.7139	Gene	1
EGF	entrez.1950	1		entrez.1950	Gene	0
KAT8	entrez.84148	5	TRUE	entrez.84148	Gene	1
CXCL2	entrez.2920	5	TRUE	entrez.2920	Gene	1
IFNL3	entrez.282617	2		entrez.282617	Gene	0
UQCRFS1	entrez.7386	5	TRUE	entrez.7386	Gene	1
CBLN4	entrez.140689	5		entrez.140689	Gene	0
ACTB	entrez.60	5	TRUE	entrez.60	Gene	1
LRRK2	entrez.120892	4		entrez.120892	Gene	0
TNS2	entrez.23371	1		entrez.23371	Gene	0
CES3	entrez.23491	5	TRUE	entrez.23491	Gene	1
TLE5	entrez.166	5	TRUE	entrez.166	Gene	1
KLF4	entrez.9314	5	TRUE	entrez.9314	Gene	1
TNNI3	entrez.7137	5	TRUE	entrez.7137	Gene	1
XDH	entrez.7498	5	TRUE	entrez.7498	Gene	1
H2BC4	entrez.8347	1		entrez.8347	Gene	0
ACTA2	entrez.59	1		entrez.59	Gene	0
ACTA1	entrez.58	3		entrez.58	Gene	0
TERF1	entrez.7013	2		entrez.7013	Gene	0
H2BC6	entrez.8344	2		entrez.8344	Gene	0
H2BC7	entrez.8343	2		entrez.8343	Gene	0
TNFRSF1A	entrez.7132	5	TRUE	entrez.7132	Gene	1
DNM1L	entrez.10059	5	TRUE	entrez.10059	Gene	1
PDE4DIP	entrez.9659	5		entrez.9659	Gene	0
GRK2	entrez.156	5	TRUE	entrez.156	Gene	1
ADRB3	entrez.155	5	TRUE	entrez.155	Gene	1
ARHGDIB	entrez.397	5	TRUE	entrez.397	Gene	1
ADRB2	entrez.154	5	TRUE	entrez.154	Gene	1

PAK2	entrez.5062	3		entrez.5062	Gene	0
ADRB1	entrez.153	5	TRUE	entrez.153	Gene	1
ADRA2C	entrez.152	5	TRUE	entrez.152	Gene	1
SMAD9	entrez.4093	5	TRUE	entrez.4093	Gene	1
TPRA1	entrez.131601	1		entrez.131601	Gene	0
TMEFF1	entrez.8577	1		entrez.8577	Gene	0
PAPPA	entrez.5069	5	TRUE	entrez.5069	Gene	1
TNF	entrez.7124	5	TRUE	entrez.7124	Gene	1
CUL3	entrez.8452	1		entrez.8452	Gene	0
ACLY	entrez.47	5	TRUE	entrez.47	Gene	1
DPP4	entrez.1803	1		entrez.1803	Gene	0
RPL17	entrez.6139	5	TRUE	entrez.6139	Gene	1
RHOC	entrez.389	5	TRUE	entrez.389	Gene	1
UCP1	entrez.7350	5	TRUE	entrez.7350	Gene	1
PDPK1	entrez.5170	5	TRUE	entrez.5170	Gene	1
TSPAN11	entrez.441631	1		entrez.441631	Gene	0
ACE2	entrez.59272	2		entrez.59272	Gene	0
UGCG	entrez.7357	5	TRUE	entrez.7357	Gene	1
SMAD4	entrez.4089	5	TRUE	entrez.4089	Gene	1
NRIP1	entrez.8204	5	TRUE	entrez.8204	Gene	1
SMAD2	entrez.4087	1		entrez.4087	Gene	0
SERPINE1	entrez.5054	5	TRUE	entrez.5054	Gene	1
SMAD1	entrez.4086	5	TRUE	entrez.4086	Gene	1
TMPO	entrez.7112	5	TRUE	entrez.7112	Gene	1
RYR2	entrez.6262	5	TRUE	entrez.6262	Gene	1
PIK3CG	entrez.5294	5	TRUE	entrez.5294	Gene	1
BAG3	entrez.9531	1		entrez.9531	Gene	0
ACACA	entrez.31	5	TRUE	entrez.31	Gene	1
FEZ2	entrez.9637	5	TRUE	entrez.9637	Gene	1
ISG15	entrez.9636	2		entrez.9636	Gene	0
GDF15	entrez.9518	5	TRUE	entrez.9518	Gene	1
HDAC4	entrez.9759	5	TRUE	entrez.9759	Gene	1
EDNRB	entrez.1910	5	TRUE	entrez.1910	Gene	1

AREG	entrez.374	5	TRUE	entrez.374	Gene	1
MDM2	entrez.4193	1		entrez.4193	Gene	0
PDHA1	entrez.5160	5	TRUE	entrez.5160	Gene	1
RXRG	entrez.6258	5	TRUE	entrez.6258	Gene	1
CX3CL1	entrez.6376	5	TRUE	entrez.6376	Gene	1
PDK4	entrez.5166	5	TRUE	entrez.5166	Gene	1
BHLHE40	entrez.8553	5	TRUE	entrez.8553	Gene	1
RTN1	entrez.6252	5	TRUE	entrez.6252	Gene	1
NUP43	entrez.348995	3		entrez.348995	Gene	0
PDHB	entrez.5162	1		entrez.5162	Gene	0
TUBA1C	entrez.84790	5	TRUE	entrez.84790	Gene	1
UBE2I	entrez.7329	1		entrez.7329	Gene	0
EDN1	entrez.1906	5	TRUE	entrez.1906	Gene	1
RGCC	entrez.28984	5	TRUE	entrez.28984	Gene	1
ATP2A2	entrez.488	5	TRUE	entrez.488	Gene	1
ATP2A1	entrez.487	5	TRUE	entrez.487	Gene	1
HIVEP2	entrez.3097	5	TRUE	entrez.3097	Gene	1
HK1	entrez.3098	5	TRUE	entrez.3098	Gene	1
HK2	entrez.3099	5	TRUE	entrez.3099	Gene	1
PEBP1	entrez.5037	5	TRUE	entrez.5037	Gene	1
EDNRA	entrez.1909	5	TRUE	entrez.1909	Gene	1
P4HB	entrez.5034	5	TRUE	entrez.5034	Gene	1
HIF1A	entrez.3091	5	TRUE	entrez.3091	Gene	1
VWF	entrez.7450	5	TRUE	entrez.7450	Gene	1
CCL2	entrez.6347	5	TRUE	entrez.6347	Gene	1
CCL3	entrez.6348	5	TRUE	entrez.6348	Gene	1
AQP1	entrez.358	5	TRUE	entrez.358	Gene	1
ATP1A3	entrez.478	5	TRUE	entrez.478	Gene	1
ATP1A1	entrez.476	5	TRUE	entrez.476	Gene	1
CCND1	entrez.595	5	TRUE	entrez.595	Gene	1
APP	entrez.351	5		entrez.351	Gene	0
DNMT3L	entrez.29947	1		entrez.29947	Gene	0
NRG1	entrez.3084	5	TRUE	entrez.3084	Gene	1

RLN3	entrez.117579	5	TRUE	entrez.117579	Gene	1
SIAH1	entrez.6477	2		entrez.6477	Gene	0
CCT3	entrez.7203	2		entrez.7203	Gene	0
PDE4B	entrez.5142	5	TRUE	entrez.5142	Gene	1
SNAP23	entrez.8773	5	TRUE	entrez.8773	Gene	1
CCL4	entrez.6351	5	TRUE	entrez.6351	Gene	1
HGF	entrez.3082	5	TRUE	entrez.3082	Gene	1
SQSTM1	entrez.8878	2		entrez.8878	Gene	0
ALDOA	entrez.226	5	TRUE	entrez.226	Gene	1
TCTN2	entrez.79867	2		entrez.79867	Gene	0
SERPINC1	entrez.462	5	TRUE	entrez.462	Gene	1
APOC1	entrez.341	5	TRUE	entrez.341	Gene	1
MC4R	entrez.4160	1		entrez.4160	Gene	0
CBLN2	entrez.147381	5	TRUE	entrez.147381	Gene	1
PPP1CA	entrez.5499	3		entrez.5499	Gene	0
SHC1	entrez.6464	3		entrez.6464	Gene	0
UBA52	entrez.7311	1		entrez.7311	Gene	0
SLC25A11	entrez.8402	1		entrez.8402	Gene	0
EZR	entrez.7430	3		entrez.7430	Gene	0
PPM1B	entrez.5495	5	TRUE	entrez.5495	Gene	1
VIM	entrez.7431	5	TRUE	entrez.7431	Gene	1
ZFP36	entrez.7538	5	TRUE	entrez.7538	Gene	1
SGTA	entrez.6449	1		entrez.6449	Gene	0
MIDEAS	entrez.91748	1		entrez.91748	Gene	0
PPIB	entrez.5479	1		entrez.5479	Gene	0
VCL	entrez.7414	5	TRUE	entrez.7414	Gene	1
VCP	entrez.7415	1		entrez.7415	Gene	0
MAPK8	entrez.5599	2		entrez.5599	Gene	0
PGM1	entrez.5236	5	TRUE	entrez.5236	Gene	1
APLP2	entrez.334	5	TRUE	entrez.334	Gene	1
ALB	entrez.213	5	TRUE	entrez.213	Gene	1
HDAC1	entrez.3065	5	TRUE	entrez.3065	Gene	1
SPR	entrez.6697	1		entrez.6697	Gene	0

TMEM17	entrez.200728	3		entrez.200728	Gene	0
VEGFA	entrez.7422	5	TRUE	entrez.7422	Gene	1
MVP	entrez.9961	2		entrez.9961	Gene	0
YWHAB	entrez.7529	2		entrez.7529	Gene	0
AKT1	entrez.207	3		entrez.207	Gene	0
PCK1	entrez.5105	5	TRUE	entrez.5105	Gene	1
PPARG	entrez.5468	5	TRUE	entrez.5468	Gene	1
SPARC	entrez.6678	5	TRUE	entrez.6678	Gene	1
CLEC10A	entrez.10462	5	TRUE	entrez.10462	Gene	1
APCS	entrez.325	5	TRUE	entrez.325	Gene	1
AK1	entrez.203	5	TRUE	entrez.203	Gene	1
SIRT1	entrez.23411	5	TRUE	entrez.23411	Gene	1
VCAM1	entrez.7412	5	TRUE	entrez.7412	Gene	1
YWHAZ	entrez.7534	3		entrez.7534	Gene	0
YWHAE	entrez.7531	1		entrez.7531	Gene	0
MAPK3	entrez.5595	2		entrez.5595	Gene	0
PCNA	entrez.5111	1		entrez.5111	Gene	0
YWHAG	entrez.7532	2		entrez.7532	Gene	0
MAS1	entrez.4142	1		entrez.4142	Gene	0
PPAT	entrez.5471	5	TRUE	entrez.5471	Gene	1
CCT8	entrez.10694	1		entrez.10694	Gene	0
KSR1	entrez.8844	1		entrez.8844	Gene	0
RAB7A	entrez.7879	5	TRUE	entrez.7879	Gene	1
SLC9A1	entrez.6548	5	TRUE	entrez.6548	Gene	1
PRKAR2B	entrez.5577	5	TRUE	entrez.5577	Gene	1
SLC8A1	entrez.6546	5	TRUE	entrez.6546	Gene	1
XPO1	entrez.7514	1		entrez.7514	Gene	0
ID3	entrez.3399	5	TRUE	entrez.3399	Gene	1
HMGCS2	entrez.3158	5	TRUE	entrez.3158	Gene	1
RXFP3	entrez.51289	3		entrez.51289	Gene	0
AVPR2	entrez.554	5	TRUE	entrez.554	Gene	1
AVP	entrez.551	5	TRUE	entrez.551	Gene	1
HMOX1	entrez.3162	5	TRUE	entrez.3162	Gene	1

FASN	entrez.2194	5	TRUE	entrez.2194	Gene	1
NR4A1	entrez.3164	5	TRUE	entrez.3164	Gene	1
PITPNB	entrez.23760	1		entrez.23760	Gene	0
CUL7	entrez.9820	1		entrez.9820	Gene	0
KITLG	entrez.4254	5	TRUE	entrez.4254	Gene	1
PRKCE	entrez.5581	5	TRUE	entrez.5581	Gene	1
PFKFB3	entrez.5209	5	TRUE	entrez.5209	Gene	1
PLAT	entrez.5327	5	TRUE	entrez.5327	Gene	1
PSMB1	entrez.5689	5	TRUE	entrez.5689	Gene	1
MX1	entrez.4599	5	TRUE	entrez.4599	Gene	1
RETN	entrez.56729	5	TRUE	entrez.56729	Gene	1
TOMM20	entrez.9804	4		entrez.9804	Gene	0
STAT5A	entrez.6776	5	TRUE	entrez.6776	Gene	1
MSN	entrez.4478	5	TRUE	entrez.4478	Gene	1
HMGB1	entrez.3146	5	TRUE	entrez.3146	Gene	1
BNIP3L	entrez.665	1		entrez.665	Gene	0
HSD17B10	entrez.3028	1		entrez.3028	Gene	0
HADHA	entrez.3030	5	TRUE	entrez.3030	Gene	1
ID1	entrez.3397	5	TRUE	entrez.3397	Gene	1
PFKM	entrez.5213	5	TRUE	entrez.5213	Gene	1
NXF1	entrez.10482	2		entrez.10482	Gene	0
LMNA	entrez.4000	1		entrez.4000	Gene	0
PSMB5	entrez.5693	5	TRUE	entrez.5693	Gene	1
SOD2	entrez.6648	5	TRUE	entrez.6648	Gene	1
SOD3	entrez.6649	5	TRUE	entrez.6649	Gene	1
BMPR2	entrez.659	5	TRUE	entrez.659	Gene	1
SOD1	entrez.6647	5	TRUE	entrez.6647	Gene	1
BMPR1B	entrez.658	5	TRUE	entrez.658	Gene	1
MEP1A	entrez.4224	3		entrez.4224	Gene	0
SELP	entrez.6403	5	TRUE	entrez.6403	Gene	1
BTRC	entrez.8945	1		entrez.8945	Gene	0
CCND2	entrez.894	5	TRUE	entrez.894	Gene	1
EIF2AK4	entrez.440275	5	TRUE	entrez.440275	Gene	1

FABP5	entrez.2171	5	TRUE	entrez.2171	Gene	1
RXFP4	entrez.339403	2		entrez.339403	Gene	0
ICAM1	entrez.3383	5	TRUE	entrez.3383	Gene	1
EPHX2	entrez.2053	5	TRUE	entrez.2053	Gene	1
PON1	entrez.5444	5	TRUE	entrez.5444	Gene	1
STAT3	entrez.6774	3		entrez.6774	Gene	0
POMC	entrez.5443	5	TRUE	entrez.5443	Gene	1
SLC6A6	entrez.6533	5	TRUE	entrez.6533	Gene	1
PLA2G2A	entrez.5320	5	TRUE	entrez.5320	Gene	1
TAP1	entrez.6890	5	TRUE	entrez.6890	Gene	1
G3BP1	entrez.10146	1		entrez.10146	Gene	0
ARRB1	entrez.408	2		entrez.408	Gene	0
TMEM216	entrez.51259	1		entrez.51259	Gene	0
BMP1	entrez.649	1		entrez.649	Gene	0
TUBA1A	entrez.7846	5	TRUE	entrez.7846	Gene	1
ARNTL	entrez.406	5	TRUE	entrez.406	Gene	1
PTPRA	entrez.5786	5	TRUE	entrez.5786	Gene	1
ADM2	entrez.79924	5	TRUE	entrez.79924	Gene	1
IGFBP7	entrez.3490	1		entrez.3490	Gene	0
CTSC	entrez.1075	5	TRUE	entrez.1075	Gene	1
HLA-E	entrez.3133	2		entrez.3133	Gene	0
MEOX2	entrez.4223	5	TRUE	entrez.4223	Gene	1
SELE	entrez.6401	5	TRUE	entrez.6401	Gene	1
SOX17	entrez.64321	5	TRUE	entrez.64321	Gene	1
ALDH6A1	entrez.4329	5	TRUE	entrez.4329	Gene	1
HDAC5	entrez.10014	5	TRUE	entrez.10014	Gene	1
OPTN	entrez.10133	1		entrez.10133	Gene	0
TENT5A	entrez.55603	2		entrez.55603	Gene	0
CDH1	entrez.999	1		entrez.999	Gene	0
PPP3R1	entrez.5534	5	TRUE	entrez.5534	Gene	1
EZH2	entrez.2146	1		entrez.2146	Gene	0
HTR2B	entrez.3357	5	TRUE	entrez.3357	Gene	1
IGF1	entrez.3479	5	TRUE	entrez.3479	Gene	1

ENSA	entrez.2029	5	TRUE	entrez.2029	Gene	1
CDC20	entrez.991	1		entrez.991	Gene	0
F2RL1	entrez.2150	5	TRUE	entrez.2150	Gene	1
F3	entrez.2152	5	TRUE	entrez.2152	Gene	1
FHL2	entrez.2274	2		entrez.2274	Gene	0
IGFBP3	entrez.3486	5	TRUE	entrez.3486	Gene	1
NCL	entrez.4691	5	TRUE	entrez.4691	Gene	1
TRIM25	entrez.7706	5		entrez.7706	Gene	0
MMP9	entrez.4318	5	TRUE	entrez.4318	Gene	1
CDC5L	entrez.988	2		entrez.988	Gene	0
SRPK2	entrez.6733	2		entrez.6733	Gene	0
NOX1	entrez.27035	5	TRUE	entrez.27035	Gene	1
IL10RB	entrez.3588	5		entrez.3588	Gene	0
CDK1	entrez.983	1		entrez.983	Gene	0
ACOT2	entrez.10965	5	TRUE	entrez.10965	Gene	1
CEBPD	entrez.1052	5	TRUE	entrez.1052	Gene	1
ENG	entrez.2022	5	TRUE	entrez.2022	Gene	1
ENO1	entrez.2023	5	TRUE	entrez.2023	Gene	1
NR3C2	entrez.4306	5	TRUE	entrez.4306	Gene	1
RAC1	entrez.5879	5	TRUE	entrez.5879	Gene	1
CAV3	entrez.859	1		entrez.859	Gene	0
PPP2CA	entrez.5515	5	TRUE	entrez.5515	Gene	1
CAV1	entrez.857	5	TRUE	entrez.857	Gene	1
CD81	entrez.975	1		entrez.975	Gene	0
TMED2	entrez.10959	5	TRUE	entrez.10959	Gene	1
CRK	entrez.1398	5	TRUE	entrez.1398	Gene	1
FGF1	entrez.2246	5	TRUE	entrez.2246	Gene	1
FGF2	entrez.2247	5	TRUE	entrez.2247	Gene	1
CKM	entrez.1158	5	TRUE	entrez.1158	Gene	1
NPTN	entrez.27020	5	TRUE	entrez.27020	Gene	1
IFNG	entrez.3458	5	TRUE	entrez.3458	Gene	1
ELN	entrez.2006	5	TRUE	entrez.2006	Gene	1
IFNGR1	entrez.3459	5	TRUE	entrez.3459	Gene	1

SNW1	entrez.22938	1		entrez.22938	Gene	0
NFKBIA	entrez.4792	5	TRUE	entrez.4792	Gene	1
SYK	entrez.6850	1		entrez.6850	Gene	0
NFKB1	entrez.4790	1		entrez.4790	Gene	0
RAB5A	entrez.5868	5	TRUE	entrez.5868	Gene	1
CAT	entrez.847	5	TRUE	entrez.847	Gene	1
PTH1R	entrez.5745	1		entrez.5745	Gene	0
ITGB1	entrez.3688	5	TRUE	entrez.3688	Gene	1
PPP1R1A	entrez.5502	5	TRUE	entrez.5502	Gene	1
CBLN3	entrez.643866	5		entrez.643866	Gene	0
PTGS2	entrez.5743	5	TRUE	entrez.5743	Gene	1
PTGS1	entrez.5742	5	TRUE	entrez.5742	Gene	1
GAPDH	entrez.2597	5	TRUE	entrez.2597	Gene	1
CIDEA	entrez.1149	5	TRUE	entrez.1149	Gene	1
IL6	entrez.3569	5	TRUE	entrez.3569	Gene	1
HSPD1	entrez.3329	5	TRUE	entrez.3329	Gene	1
CKB	entrez.1152	5	TRUE	entrez.1152	Gene	1
IL6ST	entrez.3572	5	TRUE	entrez.3572	Gene	1
EIF2A	entrez.83939	5	TRUE	entrez.83939	Gene	1
CIRBP	entrez.1153	5	TRUE	entrez.1153	Gene	1
NFE2L2	entrez.4780	5	TRUE	entrez.4780	Gene	1
ITGB3	entrez.3690	5	TRUE	entrez.3690	Gene	1
PRL	entrez.5617	5	TRUE	entrez.5617	Gene	1
PDLIM5	entrez.10611	5	TRUE	entrez.10611	Gene	1
YWHAQ	entrez.10971	4		entrez.10971	Gene	0
C1QB	entrez.713	5	TRUE	entrez.713	Gene	1
C1QA	entrez.712	1		entrez.712	Gene	0
CPT1B	entrez.1375	5	TRUE	entrez.1375	Gene	1
IL1B	entrez.3553	5	TRUE	entrez.3553	Gene	1
CAPZB	entrez.832	5	TRUE	entrez.832	Gene	1
CDH13	entrez.1012	2		entrez.1012	Gene	0
CD38	entrez.952	5	TRUE	entrez.952	Gene	1
HSPA8	entrez.3312	5	TRUE	entrez.3312	Gene	1

HSPA9	entrez.3313	5	TRUE	entrez.3313	Gene	1
CTNNB1	entrez.1499	4		entrez.1499	Gene	0
HSPB1	entrez.3315	5	TRUE	entrez.3315	Gene	1
BAG6	entrez.7917	1		entrez.7917	Gene	0
PTGIR	entrez.5739	1		entrez.5739	Gene	0
ATP13A3	entrez.79572	5	TRUE	entrez.79572	Gene	1
FOS	entrez.2353	2		entrez.2353	Gene	0
PTH	entrez.5741	5	TRUE	entrez.5741	Gene	1
NFATC1	entrez.4772	2		entrez.4772	Gene	0
NPPB	entrez.4879	5	TRUE	entrez.4879	Gene	1
HSPA5	entrez.3309	2		entrez.3309	Gene	0
CD36	entrez.948	5	TRUE	entrez.948	Gene	1
NPPA	entrez.4878	5	TRUE	entrez.4878	Gene	1
ZEB1	entrez.6935	5	TRUE	entrez.6935	Gene	1
CANX	entrez.821	5	TRUE	entrez.821	Gene	1
IL22RA1	entrez.58985	3		entrez.58985	Gene	0
NOP56	entrez.10528	1		entrez.10528	Gene	0
HSPA1A	entrez.3303	5	TRUE	entrez.3303	Gene	1
MAP2K7	entrez.5609	5	TRUE	entrez.5609	Gene	1
HSPA2	entrez.3306	5	TRUE	entrez.3306	Gene	1
SULT1A1	entrez.6817	5	TRUE	entrez.6817	Gene	1
PTEN	entrez.5728	3		entrez.5728	Gene	0
CCN2	entrez.1490	5	TRUE	entrez.1490	Gene	1
GABARAPL1	entrez.23710	1		entrez.23710	Gene	0
IL1A	entrez.3552	5	TRUE	entrez.3552	Gene	1
REN	entrez.5972	5	TRUE	entrez.5972	Gene	1
RSAD2	entrez.91543	5	TRUE	entrez.91543	Gene	1
NPR1	entrez.4881	5	TRUE	entrez.4881	Gene	1
RAB7B	entrez.338382	5	TRUE	entrez.338382	Gene	1
KDR	entrez.3791	5	TRUE	entrez.3791	Gene	1
IRF1	entrez.3659	5	TRUE	entrez.3659	Gene	1
NPM1	entrez.4869	2		entrez.4869	Gene	0
CAMK2D	entrez.817	5	TRUE	entrez.817	Gene	1

PYGB	entrez.5834	5	TRUE	entrez.5834	Gene	1
KCNK3	entrez.3777	5	TRUE	entrez.3777	Gene	1
PEX5	entrez.5830	1		entrez.5830	Gene	0
CALR	entrez.811	5	TRUE	entrez.811	Gene	1
CST6	entrez.1474	3		entrez.1474	Gene	0
CCR5	entrez.1234	1		entrez.1234	Gene	0
FBLN5	entrez.10516	5	TRUE	entrez.10516	Gene	1
RAB1B	entrez.81876	5	TRUE	entrez.81876	Gene	1
GHR	entrez.2690	5	TRUE	entrez.2690	Gene	1
CDH2	entrez.1000	5	TRUE	entrez.1000	Gene	1
UCN2	entrez.90226	5	TRUE	entrez.90226	Gene	1
CD9	entrez.928	1		entrez.928	Gene	0
NEDD4	entrez.4734	5	TRUE	entrez.4734	Gene	1
KCNJ8	entrez.3764	5	TRUE	entrez.3764	Gene	1
CALM1	entrez.801	1		entrez.801	Gene	0
OLR1	entrez.4973	5	TRUE	entrez.4973	Gene	1
CD4	entrez.920	4		entrez.920	Gene	0
A2M	entrez.2	1		entrez.2	Gene	0
RBP4	entrez.5950	5	TRUE	entrez.5950	Gene	1
NOS3	entrez.4846	5	TRUE	entrez.4846	Gene	1
NOS2	entrez.4843	5	TRUE	entrez.4843	Gene	1
NOS1	entrez.4842	1		entrez.4842	Gene	0
INS	entrez.3630	5	TRUE	entrez.3630	Gene	1
PPARGC1A	entrez.10891	5	TRUE	entrez.10891	Gene	1
GNB2	entrez.2783	1		entrez.2783	Gene	0
UBQLN4	entrez.56893	1		entrez.56893	Gene	0
MYC	entrez.4609	5		entrez.4609	Gene	0
VCAN	entrez.1462	3		entrez.1462	Gene	0

Table2 Candidate drugs sorted by the trust rank algorithm

displayName	drugGroups	name	rank	score	selected	type
Sunitinib	approved, investigational	drugbank.DB01268	1	3.12E-04	FALSE	Drug
Nintedanib	approved	drugbank.DB09079	2	2.71E-04	FALSE	Drug
Midostaurin	approved, investigational	drugbank.DB06595	3	2.47E-04	FALSE	Drug
Bosutinib	approved	drugbank.DB06616	4	2.39E-04	FALSE	Drug
Dasatinib	approved, investigational	drugbank.DB01254	5	1.74E-04	FALSE	Drug
Sorafenib	approved, investigational	drugbank.DB00398	6	1.65E-04	FALSE	Drug
Crizotinib	approved	drugbank.DB08865	7	1.64E-04	FALSE	Drug
Ruxolitinib	approved	drugbank.DB08877	8	1.44E-04	FALSE	Drug
Vandetanib	approved	drugbank.DB05294	9	1.37E-04	FALSE	Drug
Neratinib	approved, investigational	drugbank.DB11828	10	1.29E-04	FALSE	Drug
Pazopanib	approved	drugbank.DB06589	11	1.26E-04	FALSE	Drug
Axitinib	approved, investigational	drugbank.DB06626	12	1.18E-04	FALSE	Drug
Erlotinib	approved, investigational	drugbank.DB00530	13	1.17E-04	FALSE	Drug
Tamoxifen	approved	drugbank.DB00675	14	1.09E-04	FALSE	Drug
Astemizole	approved, withdrawn	drugbank.DB00637	15	1.07E-04	FALSE	Drug
Chlorpromazine	approved, investigational, vet_approved	drugbank.DB00477	16	1.05E-04	FALSE	Drug
Clotrimazole	approved, vet_approved	drugbank.DB00257	17	1.03E-04	FALSE	Drug
Amiodarone	approved, investigational	drugbank.DB01118	18	9.95E-05	FALSE	Drug
Miconazole	approved, investigational, vet_approved	drugbank.DB01110	19	9.48E-05	FALSE	Drug
Imatinib	approved	drugbank.DB00619	20	8.80E-05	FALSE	Drug
Nilotinib	approved, investigational	drugbank.DB04868	21	8.32E-05	FALSE	Drug
Econazole	approved	drugbank.DB01127	22	8.05E-05	FALSE	Drug
Gefitinib	approved, investigational	drugbank.DB00317	23	7.90E-05	FALSE	Drug
Tioguanine	approved	drugbank.DB00352	24	7.71E-05	FALSE	Drug
Metformin	approved	drugbank.DB00331	25	6.99E-05	FALSE	Drug
Raloxifene	approved, investigational	drugbank.DB00481	26	6.49E-05	FALSE	Drug

Fluphenazine	approved	drugbank.DB00623	27	6.49E-05	FALSE	Drug
Captopril	approved	drugbank.DB01197	28	6.34E-05	FALSE	Drug
Haloperidol	approved	drugbank.DB00502	29	6.32E-05	FALSE	Drug
Diethylstilbestrol	approved, investigational	drugbank.DB00255	30	6.27E-05	FALSE	Drug
Allopurinol	approved	drugbank.DB00437	31	6.12E-05	FALSE	Drug
Febuxostat	approved	drugbank.DB04854	32	6.09E-05	FALSE	Drug
Isoflurane	approved, vet_approved	drugbank.DB00753	33	5.56E-05	FALSE	Drug
Cyproheptadine	approved	drugbank.DB00434	34	5.51E-05	FALSE	Drug
Thioridazine	approved, withdrawn	drugbank.DB00679	35	5.43E-05	FALSE	Drug
Propafenone	approved	drugbank.DB01182	36	5.38E-05	FALSE	Drug
Desflurane	approved	drugbank.DB01189	37	5.27E-05	FALSE	Drug
Halothane	approved, vet_approved	drugbank.DB01159	37	5.27E-05	FALSE	Drug
Losartan	approved	drugbank.DB00678	38	5.26E-05	FALSE	Drug
Clozapine	approved	drugbank.DB00363	39	5.26E-05	FALSE	Drug
Sevoflurane	approved, vet_approved	drugbank.DB01236	40	5.15E-05	FALSE	Drug
Clomifene	approved, investigational	drugbank.DB00882	41	5.15E-05	FALSE	Drug
Celecoxib	approved, investigational	drugbank.DB00482	42	5.09E-05	FALSE	Drug
Pimozide	approved	drugbank.DB01100	43	5.06E-05	FALSE	Drug
Risperidone	approved, investigational	drugbank.DB00734	44	5.05E-05	FALSE	Drug
Mezlocillin	approved, investigational	drugbank.DB00948	45	5.02E-05	FALSE	Drug
Ceritinib	approved	drugbank.DB09063	46	4.82E-05	FALSE	Drug
Olanzapine	approved, investigational	drugbank.DB00334	47	4.77E-05	FALSE	Drug
Domperidone	approved, investigational, vet_approved	drugbank.DB01184	48	4.65E-05	FALSE	Drug
Aripiprazole	approved, investigational	drugbank.DB01238	49	4.60E-05	FALSE	Drug

Table3 The Active Constituents of Ginseng and Their Smiles Formula

Herb	Ingredients	Smiles
Pandx	Diop	CC(C)CCCCCOC(=O)C1=CC=CC=C1C(=O)OCCCCCC(C)C

ginseng		
Pandx ginseng	Stigmasterol	CCC(C=CC(C)C1CCC2C1(CCC3C2CC=C4C3(CCC(C4)O)C)C)C(C)C
Pandx ginseng	beta-sitosterol	CCC(CCC(C)C1CCC2C1(CCC3C2CC=C4C3(CCC(C4)O)C)C)C(C)C
Pandx ginseng	Inermin	C1C2C(C3=C(O1)C=C(C=C3)O)OC4=CC5=C(C=C24)OCO5
Pandx ginseng	kaempferol	C1=CC(=CC=C1C2=C(C(=O)C3=C(C=C(C=C3O2)O)O)O)O
Pandx ginseng	Chrysanthemaxanthin	CC1=CC(CC(C1C=CC(=CC=CC(=CC=CC=C(C)C=CC=C(C)C2C=C3C(CC(CC3(O2)C)O)(C)C)C)C)(C)C)O
Pandx ginseng	Aposiopolamine	///
Pandx ginseng	Celabenzine	C1CCN(CCCNC(=O)CC(NC1)C2=CC=CC=C2)C(=O)C3=CC=CC=C3
Pandx ginseng	Deoxyharringtonine	CC(C)CCC(CC(=O)OC)(C(=O)OC1C2C3=CC4=C(C=C3CCN5C2(CCC5)C=C1OC)OCO4)O
Pandx ginseng	Dianthramine	C1=CC(=C(C=C1O)NC2=C(C=CC(=C2)O)C(=O)O)C(=O)O
Pandx ginseng	arachidonate	CCCCCC=CCC=CCC=CCC=CCCCC(=O)O
Pandx ginseng	Frutinone A	C1=CC=C2C(=C1)C3=C(C(=O)C4=CC=CC=C4O3)C(=O)O2
Pandx ginseng	ginsenoside rh2	CC(=CCCC(C)(C1CCC2(C1C(CC3C2(CCC4C3(CCC(C4(C)C)OC5C(C(C(C(O5)CO)O)O)O)C)C)O)C)O)C
Pandx ginseng	Ginsenoside-Rh4	CC(=CCC=C(C)C1CCC2(C1C(CC3C2(CC(C4C3(CCC(C4(C)C)O)C)OC5C(C(C(C(O5)CO)O)O)O)C)O)C)C
Pandx ginseng	Girinimbin	CC1=CC2=C(C3=C1OC(C=C3)(C)C)NC4=CC=CC=C42
Pandx ginseng	Gomisin B	CC=C(C)C(=O)OC1C2=CC(=C(C(=C2C3=C(C4=C(C=C3CC(C1(C)O)C)OCO4)OC)OC)OC)OC
Pandx ginseng	malkangunin	CC1CCC(C2(C13CC(C(C2OC(=O)C4=CC=CC=C4)OC(=O)C)C(O3)(C)C)CO)O

Pandx ginseng	Panaxadiol	CC1(CCCC(O1)(C)C2CCC3(C2C(CC4C3(CCC5C4(CCC(C5(C)C)O)C)C)O)C)C
Pandx ginseng	suchilactone	COC1=C(C=C(C=C1)CC2COC(=O)C2=CC3=CC4=C(C=C3)OCO4)OC
Pandx ginseng	alexandrin_qt	CCC(CCC(C)C1CCC2C1(CCC3C2CC=C4C3(CCC(C4)OC5C(C(C(C(O5)CO)O)O)O)C)C)C(C)C
Pandx ginseng	ginsenoside Rg5	CC(=CCC=C(C)C1CCC2(C1C(CC3C2(CCC4C3(CCC(C4(C)C)OC5C(C(C(C(O5)CO)O)O)OC6C(C(C(C(O6)CO)O)O)O)C)C)O)C)C
Pandx ginseng	Fumarine	CN1CCC2=CC3=C(C=C2C(=O)CC4=C(C1)C5=C(C=C4)OCO5)OCO3
Pandx ginseng	ginsenoside Ra1	CC(=CCCC(C)(C1CCC2(C1C(CC3C2(CCC4C3(CCC(C4(C)C)OC5C(C(C(C(O5)CO)O)O)OC6C(C(C(C(O6)CO)O)O)O)C)C)O)C)OC7C(C(C(C(O7)COC8C(C(C(CO8)OC9C(C(C(CO9)O)O)O)O)O)O)O)O)C
Pandx ginseng	ginsenoside Ra3	CC(=CCCC(C)(C1CCC2(C1C(CC3C2(CCC4C3(CCC(C4(C)C)OC5C(C(C(C(O5)CO)O)O)OC6C(C(C(C(O6)CO)O)O)O)C)C)O)C)OC7C(C(C(C(O7)COC8C(C(C(C(O8)CO)O)OC9C(C(C(CO9)O)O)O)O)O)O)O)C
Pandx ginseng	ginsenoside Rb1	CC(=CCCC(C)(C1CCC2(C1C(CC3C2(CCC4C3(CCC(C4(C)C)OC5C(C(C(C(O5)CO)O)O)OC6C(C(C(C(O6)CO)O)O)O)C)C)O)C)OC7C(C(C(C(O7)COC8C(C(C(C(O8)CO)O)O)O)O)O)O)C
Pandx ginseng	ginsenoside Rc	CC(=CCCC(C)(C1CCC2(C1C(CC3C2(CCC4C3(CCC(C4(C)C)OC5C(C(C(C(O5)CO)O)O)OC6C(C(C(C(O6)CO)O)O)O)C)C)O)C)OC7C(C(C(C(O7)COC8C(C(C(O8)CO)O)O)O)O)O)C
Pandx ginseng	ginsenoside Re	CC1C(C(C(C(O1)OC2C(C(C(OC2OC3CC4(C(CC(C5C4(CCC5C(C)(CCC=C(C)C)OC6C(C(C(C(O6)CO)O)O)O)C)O)C7(C3C(C(CC7)O)(C)C)C)C)CO)O)O)O)O)O
Pandx ginseng	ginsenoside Rg2	CN1COCN=C1N[N+](=O)[O-]
Pandx ginseng	notoginsenoside Fe	CC(=CCCC(C)(C1CCC2(C1C(CC3C2(CCC4C3(CCC(C4(C)C)OC5C(C(C(C(O5)CO)O)O)O)C)C)O)C)OC6C(C(C(C(O6)COC7C(C(C(O7)CO)O)O)O)O)O)C

Table4 Protein target prediction results of active ingredients in ginseng

**Ingredients**	**Common name**	**Uniprot ID**	**Target Class**
Stigmasterol	HMGCR	P04035	Oxidoreductase

Stigmasterol	NR1H3	Q13133	Nuclear receptor
Stigmasterol	NPC1L1	Q9UHC9	Other membrane protein
Stigmasterol	CYP17A1	P05093	Cytochrome P450
Stigmasterol	AR	P10275	Nuclear receptor
Stigmasterol	SREBF2	Q12772	Unclassified protein
Stigmasterol	CYP19A1	P11511	Cytochrome P450
Stigmasterol	RORC	P51449	Nuclear receptor
Stigmasterol	ESR1	P03372	Nuclear receptor
Stigmasterol	ESR2	Q92731	Nuclear receptor
Stigmasterol	SHBG	P04278	Secreted protein
Stigmasterol	SLC6A2	P23975	Electrochemical transporter
Stigmasterol	SERPINA6	P08185	Secreted protein
Stigmasterol	PTPN1	P18031	Phosphatase
Stigmasterol	CHRM2	P08172	Family A G protein-coupled receptor
Stigmasterol	RORA	P35398	Nuclear receptor
Stigmasterol	BCHE	P06276	Hydrolase
Stigmasterol	SLC6A4	P31645	Electrochemical transporter
Stigmasterol	ACHE	P22303	Hydrolase
Stigmasterol	CYP2C19	P33261	Cytochrome P450
Stigmasterol	G6PD	P11413	Enzyme
Stigmasterol	CDC25A	P30304	Phosphatase
Stigmasterol	CES2	O00748	Enzyme
Stigmasterol	NR1H2	P55055	Nuclear receptor
Stigmasterol	VDR	P11473	Nuclear receptor
Stigmasterol	PTGER1	P34995	Family A G protein-coupled receptor
Stigmasterol	PTGER2	P43116	Family A G protein-coupled receptor
Stigmasterol	HSD11B1	P28845	Enzyme
Stigmasterol	PPARD	Q03181	Nuclear receptor
Stigmasterol	GLRA1	P23415	Ligand-gated ion channel
Stigmasterol	SQLE	Q14534	Enzyme
Stigmasterol	NR1I3	Q14994	Nuclear receptor
Stigmasterol	DHCR7	Q9UBM7	Enzyme
Stigmasterol	PTPN6	P29350	Phosphatase

Stigmasterol	NR3C1	P04150	Nuclear receptor
Stigmasterol	TBXAS1	P24557	Cytochrome P450
Stigmasterol	GCGR	P47871	Family B G protein-coupled receptor
Stigmasterol	NOS2	P35228	Enzyme
Stigmasterol	HSD11B2	P80365	Enzyme
Stigmasterol	POLB	P06746	Enzyme
Diop	PRKCD	Q05655	Kinase
Diop	PTPN2	P17706	Phosphatase
Diop	AR	P10275	Nuclear receptor
Diop	PTPN1	P18031	Phosphatase
Diop	KCNK2	O95069	Voltage-gated ion channel
beta-sitosterol	HMGCR	P04035	Oxidoreductase
beta-sitosterol	CYP51A1	Q16850	Cytochrome P450
beta-sitosterol	AR	P10275	Nuclear receptor
beta-sitosterol	NPC1L1	Q9UHC9	Other membrane protein
beta-sitosterol	NR1H3	Q13133	Nuclear receptor
beta-sitosterol	CYP17A1	P05093	Cytochrome P450
beta-sitosterol	RORC	P51449	Nuclear receptor
beta-sitosterol	CYP19A1	P11511	Cytochrome P450
beta-sitosterol	ESR2	Q92731	Nuclear receptor
beta-sitosterol	ESR1	P03372	Nuclear receptor
beta-sitosterol	SHBG	P04278	Secreted protein
beta-sitosterol	SREBF2	Q12772	Unclassified protein
beta-sitosterol	CYP2C19	P33261	Cytochrome P450
beta-sitosterol	SLC6A2	P23975	Electrochemical transporter
beta-sitosterol	BCHE	P06276	Hydrolase
beta-sitosterol	RORA	P35398	Nuclear receptor
beta-sitosterol	PTPN1	P18031	Phosphatase
beta-sitosterol	SERPINA6	P08185	Secreted protein
beta-sitosterol	SLC6A4	P31645	Electrochemical transporter
beta-sitosterol	G6PD	P11413	Enzyme
beta-sitosterol	NR1I3	Q14994	Nuclear receptor
beta-sitosterol	CHRM2	P08172	Family A G protein-coupled receptor

beta-sitosterol	ACHE	P22303	Hydrolase
beta-sitosterol	VDR	P11473	Nuclear receptor
beta-sitosterol	CES2	O00748	Enzyme
beta-sitosterol	NR1H2	P55055	Nuclear receptor
beta-sitosterol	CDC25A	P30304	Phosphatase
beta-sitosterol	PTGER1	P34995	Family A G protein-coupled receptor
beta-sitosterol	PTGER2	P43116	Family A G protein-coupled receptor
beta-sitosterol	HSD11B1	P28845	Enzyme
beta-sitosterol	DHCR7	Q9UBM7	Enzyme
beta-sitosterol	GLRA1	P23415	Ligand-gated ion channel
beta-sitosterol	PPARD	Q03181	Nuclear receptor
beta-sitosterol	SQLE	Q14534	Enzyme
beta-sitosterol	PTPN6	P29350	Phosphatase
beta-sitosterol	FDFT1	P37268	Enzyme
beta-sitosterol	NOS2	P35228	Enzyme
beta-sitosterol	NR3C1	P04150	Nuclear receptor
beta-sitosterol	CDC25B	P30305	Phosphatase
beta-sitosterol	SHH	Q15465	Unclassified protein
beta-sitosterol	UGT2B7	P16662	Enzyme
beta-sitosterol	HSD11B2	P80365	Enzyme
beta-sitosterol	DRD2	P14416	Family A G protein-coupled receptor
beta-sitosterol	POLB	P06746	Enzyme
ginsenoside rh2	STAT3	P40763	Transcription factor
ginsenoside rh2	IL2	P60568	Secreted protein
ginsenoside rh2	HSP90AA1	P07900	Other cytosolic protein
ginsenoside rh2	PTAFR	P25105	Family A G protein-coupled receptor
	PSEN2 PSENEN NCSTN APH1A PSEN1	P49810 Q9NZ42 Q92542 Q96BI3 P49768	
ginsenoside rh2	APH1B	Q8WW43	Protease
ginsenoside rh2	VEGFA	P15692	Secreted protein
ginsenoside rh2	FGF1	P05230	Secreted protein
ginsenoside rh2	FGF2	P09038	Secreted protein
ginsenoside rh2	HPSE	Q9Y251	Enzyme
ginsenoside rh2	ATP1A1	P05023	Primary active transporter

ginsenoside rh2	BCL2L1	Q07817	Other ion channel
ginsenoside rh2	S1PR1	P21453	Family A G protein-coupled receptor
ginsenoside rh2	RORC	P51449	Nuclear receptor
ginsenoside rh2	LGALS4	P56470	Other cytosolic protein
ginsenoside rh2	LGALS3	P17931	Other cytosolic protein
ginsenoside rh2	LGALS8	O00214	Other cytosolic protein
ginsenoside rh2	TACR2	P21452	Family A G protein-coupled receptor
ginsenoside rh2	ADORA1	P30542	Family A G protein-coupled receptor
ginsenoside rh2	SYK	P43405	Kinase
ginsenoside rh2	HSD11B2	P80365	Enzyme
ginsenosideRe	STAT3	P40763	Transcription factor
ginsenosideRe	PTAFR	P25105	Family A G protein-coupled receptor
ginsenosideRe	IL2	P60568	Secreted protein
ginsenosideRe	VEGFA	P15692	Secreted protein
ginsenosideRe	FGF1	P05230	Secreted protein
ginsenosideRe	FGF2	P09038	Secreted protein
ginsenosideRe	HPSE	Q9Y251	Enzyme
ginsenosideRe	ATP1A1	P05023	Primary active transporter
	PSEN2 PSENEN NCSTN APH1A PSEN1	P49810 Q9NZ42 Q92542 Q96BI3 P49768	
ginsenosideRe	APH1B	Q8WW43	Protease
ginsenosideRe	HSP90AA1	P07900	Other cytosolic protein
ginsenosideRe	LGALS4	P56470	Other cytosolic protein
ginsenosideRe	LGALS3	P17931	Other cytosolic protein
ginsenosideRe	LGALS8	O00214	Other cytosolic protein
ginsenosideRe	BCL2L1	Q07817	Other ion channel
ginsenosideRe	CDK1	P06493	Kinase
ginsenosideRe	HSD11B2	P80365	Enzyme
ginsenosideRe	HSD11B1	P28845	Enzyme
ginsenosideRg2	CES1	P23141	Enzyme
ginsenosideRg2	CES2	O00748	Enzyme
ginsenosideRb1	STAT3	P40763	Transcription factor
ginsenosideRb1	PTAFR	P25105	Family A G protein-coupled receptor
ginsenosideRb1	IL2	P60568	Secreted protein

ginsenosideRb1	VEGFA	P15692	Secreted protein
ginsenosideRb1	FGF1	P05230	Secreted protein
ginsenosideRb1	FGF2	P09038	Secreted protein
ginsenosideRb1	HPSE	Q9Y251	Enzyme
ginsenosideRb1	RORC	P51449	Nuclear receptor
	PSEN2 PSENEN NCSTN APH1A PSEN1	P49810 Q9NZ42 Q92542 Q96BI3 P49768	
ginsenosideRb1	APH1B	Q8WW43	Protease
ginsenosideRb1	HSP90AA1	P07900	Other cytosolic protein
ginsenosideRb1	LGALS4	P56470	Other cytosolic protein
ginsenosideRb1	LGALS3	P17931	Other cytosolic protein
ginsenosideRb1	LGALS8	O00214	Other cytosolic protein
ginsenosideRb1	BCL2L1	Q07817	Other ion channel
ginsenosideRb1	CDK1	P06493	Kinase
ginsenosideRb1	HSD11B2	P80365	Enzyme
ginsenosideRb1	HSD11B1	P28845	Enzyme
ginsenosideRb1	ATP1A1	P05023	Primary active transporter
ginsenosideRg2	CES1	P23141	Enzyme
ginsenosideRg2	CES2	O00748	Enzyme
Inermin	ALOX15	P16050	Enzyme
Inermin	PTPN1	P18031	Phosphatase
Inermin	MAPKAPK2	P49137	Kinase
Inermin	CSNK1G1	Q9HCP0	Kinase
Inermin	RPS6KA1	Q15418	Kinase
Inermin	ROCK1	Q13464	Kinase
Inermin	MAPK1	P28482	Kinase
Inermin	ERN1	O75460	Enzyme
Inermin	RET	P07949	Kinase
Inermin	AR	P10275	Nuclear receptor
Inermin	RPS6KA3	P51812	Kinase
Inermin	EZR	P15311	Unclassified protein
Inermin	ESRRA	P11474	Nuclear receptor
Inermin	ESRRB	O95718	Nuclear receptor
Inermin	DAO	P14920	Enzyme

Inermin	GRM4	Q14833	Family C G protein-coupled receptor
Inermin	CLK1	P49759	Kinase
Inermin	DYRK1B	Q9Y463	Kinase
Inermin	HDAC2	Q92769	Eraser
Inermin	TRPM8	Q7Z2W7	Voltage-gated ion channel
Inermin	TTR	P02766	Secreted protein
Inermin	DGAT1	O75907	Enzyme
Inermin	CHEK2	O96017	Kinase
Inermin	ALOX12	P18054	Enzyme
Inermin	KDR	P35968	Kinase
Inermin	HDAC4	P56524	Eraser
Inermin	GRK2	P25098	Kinase
Inermin	ALPG	P10696	Enzyme
Inermin	PLAA	Q9Y263	Unclassified protein
Inermin	CHEK1	O14757	Kinase
Inermin	WEE1	P30291	Kinase
Inermin	HSD17B3	P37058	Enzyme
Inermin	HSD17B2	P37059	Enzyme
Inermin	MTOR	P42345	Kinase
Inermin	PIK3CD	O00329	Enzyme
Inermin	PIK3CB	P42338	Enzyme
Inermin	PIK3CG	P48736	Enzyme
Inermin	PIK3CA	P42336	Enzyme
Inermin	PGF	P49763	Unclassified protein
Inermin	VEGFA	P15692	Secreted protein
Inermin	F3	P13726	Surface antigen
Inermin	TUBB1	Q9H4B7	Structural protein
Inermin	TUBB3	Q13509	Structural protein
Inermin	CCND3 CCND1 CDK4 CCND2	P30281 P24385 P11802 P30279	Other cytosolic protein
Inermin	ABL1	P00519	Kinase
Inermin	RAF1	P04049	Kinase
Inermin	JAK3	P52333	Kinase
Inermin	LCK	P06239	Kinase

Inermin	LNPEP	Q9UIQ6	Protease
Inermin	CDK2	P24941	Kinase
Inermin	PARP1	P09874	Enzyme
Inermin	PRKDC	P78527	Kinase
Inermin	HCK	P08631	Kinase
Inermin	PI4KB	Q9UBF8	Enzyme
Inermin	PIM2	Q9P1W9	Kinase
Inermin	BRAF	P15056	Kinase
Inermin	EPHB4	P54760	Kinase
Inermin	ABCB1	P08183	Primary active transporter
Inermin	MIF	P14174	Enzyme
Inermin	MMP8	P22894	Protease
Inermin	CXCR1	P25024	Family A G protein-coupled receptor
Inermin	PIM3	Q86V86	Kinase
Inermin	TBK1	Q9UHD2	Kinase
Inermin	IMPDH1	P20839	Oxidoreductase
Inermin	TYMS	P04818	Transferase
Inermin	IMPDH2	P12268	Oxidoreductase
Inermin	GUSB	P08236	Enzyme
Inermin	CA14	Q9ULX7	Lyase
Inermin	ADAM17	P78536	Protease
Inermin	ADCY5	O95622	Enzyme
Inermin	MAP3K8	P41279	Kinase
Inermin	SRC	P12931	Kinase
Inermin	COMT	P21964	Transferase
Inermin	ADORA2B	P29275	Family A G protein-coupled receptor
Inermin	HDAC5	Q9UQL6	Eraser
Inermin	HDAC7	Q8WUI4	Eraser
kaempferol	NOX4	Q9NPH5	Enzyme
kaempferol	AKR1B1	P15121	Enzyme
kaempferol	XDH	P47989	Oxidoreductase
kaempferol	TYR	P14679	Oxidoreductase
kaempferol	FLT3	P36888	Kinase

kaempferol	CA2	P00918	Lyase
kaempferol	ALOX5	P09917	Oxidoreductase
kaempferol	CA7	P43166	Lyase
kaempferol	HSD17B2	P37059	Enzyme
kaempferol	ABCC1	P33527	Primary active transporter
kaempferol	HSD17B1	P14061	Enzyme
kaempferol	AHR	P35869	Transcription factor
kaempferol	CA12	O43570	Lyase
kaempferol	ESRRA	P11474	Nuclear receptor
kaempferol	ABCB1	P08183	Primary active transporter
kaempferol	CYP1B1	Q16678	Cytochrome P450
kaempferol	ABCG2	Q9UNQ0	Primary active transporter
kaempferol	ADORA1	P30542	Family A G protein-coupled receptor
kaempferol	CA4	P22748	Lyase
kaempferol	ACHE	P22303	Hydrolase
kaempferol	MAOA	P21397	Oxidoreductase
kaempferol	GLO1	Q04760	Enzyme
kaempferol	SYK	P43405	Kinase
kaempferol	GSK3B	P49841	Kinase
kaempferol	MMP9	P14780	Protease
kaempferol	MMP2	P08253	Protease
kaempferol	ALOX15	P16050	Enzyme
kaempferol	ALOX12	P18054	Enzyme
kaempferol	PTPRS	Q13332	Phosphatase
kaempferol	ADORA2A	P29274	Family A G protein-coupled receptor
kaempferol	CDK5R1 CDK5	Q15078 Q00535	Kinase
kaempferol	CCNB3 CDK1 CCNB1 CCNB2	Q8WWL7 P06493 P14635 O95067	Other cytosolic protein
kaempferol	ARG1	P05089	Enzyme
kaempferol	GPR35	Q9HC97	Family A G protein-coupled receptor
kaempferol	ESR2	Q92731	Nuclear receptor
kaempferol	DAPK1	P53355	Kinase
kaempferol	MPG	P29372	Enzyme
kaempferol	SLC22A12	Q96S37	Electrochemical transporter

kaempferol	TTR	P02766	Secreted protein
kaempferol	AKR1B10	O60218	Enzyme
kaempferol	TNKS2	Q9H2K2	Enzyme
kaempferol	TNKS	O95271	Enzyme
kaempferol	CDK6	Q00534	Kinase
kaempferol	CDK2	P24941	Kinase
kaempferol	CYP19A1	P11511	Cytochrome P450
kaempferol	CSNK2A1	P68400	Kinase
kaempferol	EGFR	P00533	Kinase
kaempferol	AVPR2	P30518	Family A G protein-coupled receptor
kaempferol	IGF1R	P08069	Kinase
kaempferol	F2	P00734	Protease
kaempferol	PIM1	P11309	Kinase
kaempferol	AURKB	Q96GD4	Kinase
kaempferol	DRD4	P21917	Family A G protein-coupled receptor
kaempferol	MPO	P05164	Enzyme
kaempferol	PIK3R1	P27986	Enzyme
kaempferol	PYGL	P06737	Enzyme
kaempferol	CA1	P00915	Lyase
kaempferol	SRC	P12931	Kinase
kaempferol	PTK2	Q05397	Kinase
kaempferol	KDR	P35968	Kinase
kaempferol	MMP13	P45452	Protease
kaempferol	MMP3	P08254	Protease
kaempferol	CA3	P07451	Lyase
kaempferol	PLK1	P53350	Kinase
kaempferol	CA6	P23280	Lyase
kaempferol	CDK1	P06493	Kinase
kaempferol	PKN1	Q16512	Kinase
kaempferol	CA14	Q9ULX7	Lyase
kaempferol	CA9	Q16790	Lyase
kaempferol	MET	P08581	Kinase
kaempferol	NEK2	P51955	Kinase

kaempferol	CXCR1	P25024	Family A G protein-coupled receptor
kaempferol	CAMK2B	Q13554	Kinase
kaempferol	ALK	Q9UM73	Kinase
kaempferol	AKT1	P31749	Kinase
kaempferol	NEK6	Q9HC98	Kinase
kaempferol	PLA2G1B	P04054	Enzyme
kaempferol	CA5A	P35218	Lyase
kaempferol	BACE1	P56817	Protease
kaempferol	AXL	P30530	Kinase
kaempferol	NUAK1	O60285	Kinase
kaempferol	AKR1C2	P52895	Enzyme
kaempferol	AKR1C1	Q04828	Enzyme
kaempferol	AKR1C3	P42330	Enzyme
kaempferol	AKR1C4	P17516	Enzyme
kaempferol	CA13	Q8N1Q1	Lyase
kaempferol	AKR1A1	P14550	Enzyme
kaempferol	APP	P05067	Membrane receptor
kaempferol	PARP1	P09874	Enzyme
kaempferol	MMP12	P39900	Protease
kaempferol	CD38	P28907	Enzyme
kaempferol	TOP1	P11387	Isomerase
kaempferol	ESR1	P03372	Nuclear receptor
kaempferol	PTGS2	P35354	Oxidoreductase
kaempferol	CFTR	P13569	Other ion channel
kaempferol	PFKFB3	Q16875	Enzyme
kaempferol	AMY1A	P04745	Enzyme
kaempferol	GRK6	P43250	Kinase
kaempferol	TERT	O14746	Enzyme
kaempferol	MAPT	P10636	Unclassified protein
Celabenzine	DRD4	P21917	Family A G protein-coupled receptor
Celabenzine	HTR2A	P28223	Family A G protein-coupled receptor
Celabenzine	OPRK1	P41145	Family A G protein-coupled receptor
Celabenzine	SLC6A4	P31645	Electrochemical transporter

Celabenzine	OPRM1	P35372	Family A G protein-coupled receptor
Celabenzine	CCR5	P51681	Family A G protein-coupled receptor
Celabenzine	EPHX2	P34913	Protease
Celabenzine	SLC6A3	Q01959	Electrochemical transporter
Celabenzine	CTSB	P07858	Protease
Celabenzine	EGFR	P00533	Kinase
Celabenzine	ADRA1D	P25100	Family A G protein-coupled receptor
Celabenzine	ADRA1A	P35348	Family A G protein-coupled receptor
Celabenzine	ADRA1B	P35368	Family A G protein-coupled receptor
Celabenzine	CHRNA4 CHRNB2	P43681 P17787	Ligand-gated ion channel
Celabenzine	OPRD1	P41143	Family A G protein-coupled receptor
Celabenzine	PRCP	P42785	Protease
Celabenzine	CAPN1	P07384	Protease
Celabenzine	DPP4	P27487	Protease
Celabenzine	SLC6A2	P23975	Electrochemical transporter
Celabenzine	TACR1	P25103	Family A G protein-coupled receptor
Celabenzine	F2	P00734	Protease
Celabenzine	MMP8	P22894	Protease
Celabenzine	DRD2	P14416	Family A G protein-coupled receptor
Celabenzine	THRB	P10828	Nuclear receptor
Celabenzine	P2RX7	Q99572	Ligand-gated ion channel
Celabenzine	CCR3	P51677	Family A G protein-coupled receptor
Celabenzine	DRD3	P35462	Family A G protein-coupled receptor
Celabenzine	MC4R	P32245	Family A G protein-coupled receptor
Celabenzine	HSD11B1	P28845	Enzyme
Celabenzine	EP300	Q09472	Writer
Celabenzine	KCNH2	Q12809	Voltage-gated ion channel
Celabenzine	F10	P00742	Protease
Celabenzine	ITGA2B ITGB3	P08514 P05106	Membrane receptor
Celabenzine	PGGT1B FNTA	P53609 P49354	Enzyme
Celabenzine	SIGMAR1	Q99720	Membrane receptor
Celabenzine	ACACB	O00763	Ligase
Celabenzine	PSMB5	P28074	Protease

Celabenzine	CHRM2	P08172	Family A G protein-coupled receptor
Celabenzine	MLYCD	O95822	Enzyme
Celabenzine	ABCG2	Q9UNQ0	Primary active transporter
Celabenzine	SYK	P43405	Kinase
Celabenzine	CHRM3	P20309	Family A G protein-coupled receptor
Celabenzine	F7	P08709	Protease
Celabenzine	UTS2R	Q9UKP6	Family A G protein-coupled receptor
Celabenzine	HSP90AA1	P07900	Other cytosolic protein
Celabenzine	PDE10A	Q9Y233	Phosphodiesterase
Celabenzine	CCR1	P32246	Family A G protein-coupled receptor
Celabenzine	AVPR1A	P37288	Family A G protein-coupled receptor
Celabenzine	MAPK14	Q16539	Kinase
Celabenzine	NTRK1	P04629	Kinase
Celabenzine	TRPV3	Q8NET8	Voltage-gated ion channel
Celabenzine	CTSK	P43235	Protease
Celabenzine	CTSS	P25774	Protease
Celabenzine	BAZ2B	Q9UIF8	Reader
Celabenzine	CECR2	Q9BXF3	Reader
Celabenzine	BAZ2A	Q9UIF9	Reader
Celabenzine	JAK3	P52333	Kinase
Celabenzine	CYP11B2	P19099	Cytochrome P450
Celabenzine	JAK1	P23458	Kinase
Celabenzine	JAK2	O60674	Kinase
Celabenzine	PDE9A	O76083	Phosphodiesterase
Celabenzine	AURKB	Q96GD4	Kinase
Celabenzine	AURKA	O14965	Kinase
Celabenzine	L3MBTL3	Q96JM7	Reader
Celabenzine	CRHR1	P34998	Family B G protein-coupled receptor
Celabenzine	ADORA1	P30542	Family A G protein-coupled receptor
Celabenzine	KDR	P35968	Kinase
Celabenzine	PRKDC	P78527	Kinase
Celabenzine	PRKCQ	Q04759	Kinase
Celabenzine	GPBAR1	Q8TDU6	Family A G protein-coupled receptor

Celabenzine	L3MBTL1	Q9Y468	Reader
Celabenzine	CDK5R1 CDK5	Q15078 Q00535	Kinase
Celabenzine	CHRM1	P11229	Family A G protein-coupled receptor
Celabenzine	MTOR	P42345	Kinase
Celabenzine	MET	P08581	Kinase
Celabenzine	PIK3CA	P42336	Enzyme
Celabenzine	IGF1R	P08069	Kinase
Celabenzine	IMPDH2	P12268	Oxidoreductase
Celabenzine	MAPK10	P53779	Kinase
Celabenzine	PDE4D	Q08499	Phosphodiesterase
Celabenzine	PDE4C	Q08493	Phosphodiesterase
Celabenzine	PARP3	Q9Y6F1	Enzyme
Celabenzine	CREBBP	Q92793	Writer
Celabenzine	PARP4	Q9UKK3	Enzyme
Celabenzine	TNKS	O95271	Enzyme
Celabenzine	RAF1	P04049	Kinase
Celabenzine	FLT3	P36888	Kinase
Celabenzine	YES1	P07947	Kinase
Celabenzine	PLA2G2A	P14555	Enzyme
Celabenzine	BRAF	P15056	Kinase
Celabenzine	SLC6A9	P48067	Electrochemical transporter
Celabenzine	CHRM4	P08173	Family A G protein-coupled receptor
Celabenzine	PDE2A	O00408	Phosphodiesterase
Celabenzine	CALCRL	Q16602	Family B G protein-coupled receptor
Celabenzine	PDE5A	O76074	Phosphodiesterase
Celabenzine	CHRNB1 CHRNA1 CHRNG CHRND	P11230 P02708 P07510 Q07001	Ligand-gated ion channel
Celabenzine	ABCC1	P33527	Primary active transporter
Celabenzine	H1F0	P07305	Unclassified protein
Celabenzine	ALK	Q9UM73	Kinase
Celabenzine	NTRK2	Q16620	Kinase
Deoxyharringtonine	BACE1	P56817	Protease
Deoxyharringtonine	REN	P00797	Protease
Deoxyharringtonine	PAK4	O96013	Kinase

Deoxyharringtonine	BDKRB1	P46663	Family A G protein-coupled receptor
	PSEN2 PSENEN NCSTN APH1A PSEN1	P49810 Q9NZ42 Q92542 Q96BI3 P49768	
Deoxyharringtonine	APH1B	Q8WW43	Protease
Deoxyharringtonine	GSK3B	P49841	Kinase
Deoxyharringtonine	GHSR	Q92847	Family A G protein-coupled receptor
Deoxyharringtonine	ADRA1D	P25100	Family A G protein-coupled receptor
Deoxyharringtonine	ADORA1	P30542	Family A G protein-coupled receptor
Deoxyharringtonine	ADRA1A	P35348	Family A G protein-coupled receptor
Deoxyharringtonine	ADRA1B	P35368	Family A G protein-coupled receptor
Deoxyharringtonine	ADORA2A	P29274	Family A G protein-coupled receptor
Deoxyharringtonine	SYK	P43405	Kinase
Deoxyharringtonine	KDR	P35968	Kinase
Deoxyharringtonine	FGFR1	P11362	Kinase
Deoxyharringtonine	CDK9	P50750	Kinase
Deoxyharringtonine	PIK3CB	P42338	Enzyme
Deoxyharringtonine	NOX4	Q9NPH5	Enzyme
Deoxyharringtonine	PDE5A	O76074	Phosphodiesterase
Deoxyharringtonine	CNR2	P34972	Family A G protein-coupled receptor
Deoxyharringtonine	CASP8	Q14790	Protease
Deoxyharringtonine	CASP1	P29466	Protease
Deoxyharringtonine	OPRM1	P35372	Family A G protein-coupled receptor
Deoxyharringtonine	OPRD1	P41143	Family A G protein-coupled receptor
Deoxyharringtonine	UGCG	Q16739	Transferase
Deoxyharringtonine	XIAP	P98170	Other cytosolic protein
Deoxyharringtonine	BIRC2	Q13490	Enzyme
Deoxyharringtonine	AURKB	Q96GD4	Kinase
Deoxyharringtonine	AURKA	O14965	Kinase
Deoxyharringtonine	CFD	P00746	Protease
Deoxyharringtonine	TACR2	P21452	Family A G protein-coupled receptor
Deoxyharringtonine	ABCC1	P33527	Primary active transporter
Deoxyharringtonine	ACHE	P22303	Hydrolase
Deoxyharringtonine	CTSD	P07339	Protease
Deoxyharringtonine	PDE11A	Q9HCR9	Phosphodiesterase

Deoxyharringtonine	EGFR	P00533	Kinase
Deoxyharringtonine	KIF11	P52732	Other cytosolic protein
Deoxyharringtonine	ERBB2	P04626	Kinase
Deoxyharringtonine	CHRM3	P20309	Family A G protein-coupled receptor
Deoxyharringtonine	HTR4	Q13639	Family A G protein-coupled receptor
Deoxyharringtonine	SLC6A4	P31645	Electrochemical transporter
Deoxyharringtonine	HRH3	Q9Y5N1	Family A G protein-coupled receptor
Deoxyharringtonine	HTR2A	P28223	Family A G protein-coupled receptor
Deoxyharringtonine	TACR1	P25103	Family A G protein-coupled receptor
Deoxyharringtonine	CTSE	P14091	Protease
Deoxyharringtonine	IGF1R	P08069	Kinase
Deoxyharringtonine	DRD2	P14416	Family A G protein-coupled receptor
Deoxyharringtonine	MDM2	Q00987	Other nuclear protein
Deoxyharringtonine	PRKDC	P78527	Kinase
Deoxyharringtonine	MCHR1	Q99705	Family A G protein-coupled receptor
Deoxyharringtonine	GSK3A	P49840	Kinase
Deoxyharringtonine	PLAT	P00750	Protease
Deoxyharringtonine	F2	P00734	Protease
Deoxyharringtonine	F10	P00742	Protease
Deoxyharringtonine	SRC	P12931	Kinase
Deoxyharringtonine	HRH4	Q9H3N8	Family A G protein-coupled receptor
Deoxyharringtonine	BRD4	O60885	Reader
Deoxyharringtonine	HTR2C	P28335	Family A G protein-coupled receptor
Deoxyharringtonine	CCR3	P51677	Family A G protein-coupled receptor
Deoxyharringtonine	HSP90AA1	P07900	Other cytosolic protein
Deoxyharringtonine	PDE10A	Q9Y233	Phosphodiesterase
Deoxyharringtonine	RPS6KB1	P23443	Kinase
Deoxyharringtonine	BACE2	Q9Y5Z0	Protease
Deoxyharringtonine	ADRA2A	P08913	Family A G protein-coupled receptor
Deoxyharringtonine	ADRA2C	P18825	Family A G protein-coupled receptor
Deoxyharringtonine	ADRA2B	P18089	Family A G protein-coupled receptor
Deoxyharringtonine	NOS2	P35228	Enzyme
Deoxyharringtonine	CACNA2D1	P54289	Calcium channel auxiliary subunit alpha2delta

			family
Deoxyharringtonine	FNTA FNTB	P49354 P49356	Enzyme
Deoxyharringtonine	JAK3	P52333	Kinase
Deoxyharringtonine	PIK3CD	O00329	Enzyme
Deoxyharringtonine	PLAU	P00749	Protease
Deoxyharringtonine	PDE8B	O95263	Phosphodiesterase
Deoxyharringtonine	CHEK1	O14757	Kinase
Deoxyharringtonine	ADRB2	P07550	Family A G protein-coupled receptor
Deoxyharringtonine	ADRB1	P08588	Family A G protein-coupled receptor
Deoxyharringtonine	ADRB3	P13945	Family A G protein-coupled receptor
Deoxyharringtonine	ELANE	P08246	Protease
Deoxyharringtonine	CHRM2	P08172	Family A G protein-coupled receptor
Deoxyharringtonine	DRD4	P21917	Family A G protein-coupled receptor
Deoxyharringtonine	DRD3	P35462	Family A G protein-coupled receptor
Deoxyharringtonine	PRKCG	P05129	Kinase
Deoxyharringtonine	PRKCD	Q05655	Kinase
Deoxyharringtonine	PRKCA	P17252	Kinase
Deoxyharringtonine	PRKCB	P05771	Kinase
Deoxyharringtonine	PARP1	P09874	Enzyme
Deoxyharringtonine	PRKCE	Q02156	Kinase
Deoxyharringtonine	HTR1D	P28221	Family A G protein-coupled receptor
Deoxyharringtonine	ATAD2	Q6PL18	Reader
Deoxyharringtonine	HTR6	P50406	Family A G protein-coupled receptor
Deoxyharringtonine	SCN9A	Q15858	Voltage-gated ion channel
Deoxyharringtonine	MAP3K12	Q12852	Enzyme
Deoxyharringtonine	FLT3	P36888	Kinase
Deoxyharringtonine	CDK2 CCNA1 CCNA2	P24941 P78396 P20248	Other cytosolic protein
Deoxyharringtonine	PDPK1	O15530	Kinase
Deoxyharringtonine	JAK1	P23458	Kinase
Deoxyharringtonine	JAK2	O60674	Kinase
Deoxyharringtonine	PDE9A	O76083	Phosphodiesterase
Deoxyharringtonine	TYK2	P29597	Kinase
Deoxyharringtonine	PRKCQ	Q04759	Kinase

Dianthramine	AKR1C2	P52895	Enzyme
Dianthramine	CA2	P00918	Lyase
Dianthramine	AKR1C3	P42330	Enzyme
Dianthramine	CTSV	O60911	Protease
Dianthramine	CTSL	P07711	Protease
Dianthramine	AKR1C1	Q04828	Enzyme
Dianthramine	CA1	P00915	Lyase
Dianthramine	CA12	O43570	Lyase
Dianthramine	CA9	Q16790	Lyase
Dianthramine	FOLH1	Q04609	Protease
Dianthramine	PTGS2	P35354	Oxidoreductase
Dianthramine	TTR	P02766	Secreted protein
Dianthramine	MPO	P05164	Enzyme
Dianthramine	PTPN1	P18031	Phosphatase
arachidonate	FABP4	P15090	Fatty acid binding protein family
arachidonate	PPARG	P37231	Nuclear receptor
arachidonate	PPARA	Q07869	Nuclear receptor
arachidonate	PPARD	Q03181	Nuclear receptor
arachidonate	ALOX5	P09917	Oxidoreductase
arachidonate	FFAR1	O14842	Family A G protein-coupled receptor
arachidonate	FABP3	P05413	Fatty acid binding protein family
arachidonate	PTGS1	P23219	Oxidoreductase
arachidonate	CNR1	P21554	Family A G protein-coupled receptor
arachidonate	FAAH	O00519	Enzyme
arachidonate	TERT	O14746	Enzyme
arachidonate	FABP5	Q01469	Fatty acid binding protein family
arachidonate	FABP1	P07148	Fatty acid binding protein family
arachidonate	TOP1	P11387	Isomerase
arachidonate	PTGES	O14684	Enzyme
arachidonate	SCD	O00767	Enzyme
arachidonate	PTGER2	P43116	Family A G protein-coupled receptor
arachidonate	POLB	P06746	Enzyme
arachidonate	CYP26B1	Q9NR63	Cytochrome P450

arachidonate	CYP26A1	O43174	Cytochrome P450
arachidonate	CES2	O00748	Enzyme
arachidonate	ALOX12	P18054	Enzyme
arachidonate	CDC25A	P30304	Phosphatase
arachidonate	PTPN1	P18031	Phosphatase
arachidonate	PTGDR2	Q9Y5Y4	Family A G protein-coupled receptor
arachidonate	PTPN11	Q06124	Phosphatase
arachidonate	HSD11B1	P28845	Enzyme
arachidonate	PTGER1	P34995	Family A G protein-coupled receptor
arachidonate	RXRA	P19793	Nuclear receptor
arachidonate	RARG	P13631	Nuclear receptor
arachidonate	RARB	P10826	Nuclear receptor
arachidonate	RARA	P10276	Nuclear receptor
arachidonate	RXRB	P28702	Nuclear receptor
arachidonate	RXRG	P48443	Nuclear receptor
arachidonate	PTGER4	P35408	Family A G protein-coupled receptor
arachidonate	ESR2	Q92731	Nuclear receptor
arachidonate	SLC22A12	Q96S37	Electrochemical transporter
arachidonate	HSD11B2	P80365	Enzyme
arachidonate	ALOX15	P16050	Enzyme
arachidonate	TBXAS1	P24557	Cytochrome P450
arachidonate	NR1H3	Q13133	Nuclear receptor
arachidonate	FABP2	P12104	Fatty acid binding protein family
arachidonate	PTPN2	P17706	Phosphatase
arachidonate	HMGCR	P04035	Oxidoreductase
arachidonate	PTGIR	P43119	Family A G protein-coupled receptor
arachidonate	ENPP2	Q13822	Enzyme
arachidonate	PTGES2	Q9H7Z7	Enzyme
arachidonate	LTB4R	Q15722	Family A G protein-coupled receptor
arachidonate	GLRA1	P23415	Ligand-gated ion channel
arachidonate	NR3C1	P04150	Nuclear receptor
arachidonate	PRKCH	P24723	Kinase
arachidonate	AKR1B10	O60218	Enzyme

arachidonate	GRM2	Q14416	Family C G protein-coupled receptor
arachidonate	NR0B2	Q15466	Nuclear receptor
arachidonate	PLA2G4A	P47712	Enzyme
arachidonate	FFAR4	Q5NUL3	Family A G protein-coupled receptor
arachidonate	DAGLA	Q9Y4D2	Enzyme
arachidonate	TRPA1	O75762	Voltage-gated ion channel
arachidonate	OXER1	Q8TDS5	Family A G protein-coupled receptor
arachidonate	SLC16A1	P53985	Electrochemical transporter
arachidonate	PDE4A	P27815	Phosphodiesterase
arachidonate	CNR2	P34972	Family A G protein-coupled receptor
arachidonate	PTPN6	P29350	Phosphatase
	PSEN2 PSENEN NCSTN APH1A PSEN1	P49810 Q9NZ42 Q92542 Q96BI3 P49768	
arachidonate	APH1B	Q8WW43	Protease
arachidonate	DAGLB	Q8NCG7	Enzyme
arachidonate	GCG	P01275	Unclassified protein
arachidonate	TRPM8	Q7Z2W7	Voltage-gated ion channel
arachidonate	GYS1	P13807	Enzyme
arachidonate	ALOX5AP	P20292	Other cytosolic protein
arachidonate	NOS2	P35228	Enzyme
arachidonate	MMP13	P45452	Protease
arachidonate	MMP3	P08254	Protease
arachidonate	RBP4	P02753	Secreted protein
arachidonate	MMP8	P22894	Protease
arachidonate	LDHA	P00338	Enzyme
arachidonate	ESR1	P03372	Nuclear receptor
arachidonate	ACE	P12821	Protease
arachidonate	ITGAL ICAM1 ITGB2	P20701 P05362 P05107	Membrane receptor
arachidonate	AGTR1	P30556	Family A G protein-coupled receptor
arachidonate	HNF4A	P41235	Unclassified protein
arachidonate	TRPV1	Q8NER1	Voltage-gated ion channel
arachidonate	KDM2A	Q9Y2K7	Eraser
arachidonate	KDM5C	P41229	Eraser
arachidonate	PLA2G10	O15496	Enzyme

arachidonate	RORC	P51449	Nuclear receptor
arachidonate	PDE4D	Q08499	Phosphodiesterase
arachidonate	DHODH	Q02127	Oxidoreductase
arachidonate	EDNRA	P25101	Family A G protein-coupled receptor
arachidonate	PDE4B	Q07343	Phosphodiesterase
arachidonate	BMP1	P13497	Protease
arachidonate	CCKBR	P32239	Family A G protein-coupled receptor
arachidonate	MDM2	Q00987	Other nuclear protein
arachidonate	CMA1	P23946	Protease
arachidonate	CTSG	P08311	Protease
arachidonate	AR	P10275	Nuclear receptor
arachidonate	FNTA FNTB	P49354 P49356	Enzyme
arachidonate	PSEN1	P49768	Other ion channel
arachidonate	SOAT1	P35610	Enzyme
arachidonate	SOAT2	O75908	Enzyme
arachidonate	F2R	P25116	Family A G protein-coupled receptor
Frutinone A	NFKB1	P19838	Other cytosolic protein
Frutinone A	ACHE	P22303	Hydrolase
Frutinone A	CA2	P00918	Lyase
Frutinone A	CA1	P00915	Lyase
Frutinone A	CA12	O43570	Lyase
Frutinone A	CA9	Q16790	Lyase
Frutinone A	CA7	P43166	Lyase
Frutinone A	CA5B	Q9Y2D0	Lyase
Frutinone A	CA5A	P35218	Lyase
Frutinone A	CA4	P22748	Lyase
Frutinone A	CA14	Q9ULX7	Lyase
Frutinone A	CA13	Q8N1Q1	Lyase
Frutinone A	CA6	P23280	Lyase
Frutinone A	CA3	P07451	Lyase
Frutinone A	MAOA	P21397	Oxidoreductase
Frutinone A	PDE10A	Q9Y233	Phosphodiesterase
Frutinone A	PIK3CD PIK3R1	O00329 P27986	Enzyme

Frutinone A	ADORA1	P30542	Family A G protein-coupled receptor
Frutinone A	CDK2	P24941	Kinase
Frutinone A	CDK1	P06493	Kinase
Frutinone A	ESR2	Q92731	Nuclear receptor
Frutinone A	AGPAT2	O15120	Enzyme
Frutinone A	TRAP1	Q12931	Other cytosolic protein
Frutinone A	HSP90B1	P14625	Other membrane protein
Frutinone A	CTSL	P07711	Protease
Frutinone A	PDE8B	O95263	Phosphodiesterase
Frutinone A	ESR1	P03372	Nuclear receptor
Frutinone A	HSP90AA1	P07900	Other cytosolic protein
Frutinone A	ALPL	P05186	Enzyme
Frutinone A	MAPK8	P45983	Kinase
Frutinone A	MAPK10	P53779	Kinase
Frutinone A	MAPK9	P45984	Kinase
Frutinone A	CRHR1	P34998	Family B G protein-coupled receptor
Ginsenoside-Rh4	STAT3	P40763	Transcription factor
Ginsenoside-Rh4	IL2	P60568	Secreted protein
Ginsenoside-Rh4	BCL2L1	Q07817	Other ion channel
Ginsenoside-Rh4	MMP9	P14780	Protease
	PSEN2 PSENEN NCSTN APH1A PSEN1	P49810 Q9NZ42 Q92542 Q96BI3 P49768	
Ginsenoside-Rh4	APH1B	Q8WW43	Protease
Ginsenoside-Rh4	PTAFR	P25105	Family A G protein-coupled receptor
Ginsenoside-Rh4	AR	P10275	Nuclear receptor
Ginsenoside-Rh4	NR3C1	P04150	Nuclear receptor
Ginsenoside-Rh4	MET	P08581	Kinase
Ginsenoside-Rh4	ALK	Q9UM73	Kinase
Ginsenoside-Rh4	MTOR	P42345	Kinase
Ginsenoside-Rh4	PIK3CA	P42336	Enzyme
Ginsenoside-Rh4	PRF1	P14222	Other ion channel
Ginsenoside-Rh4	HSP90AA1	P07900	Other cytosolic protein
Ginsenoside-Rh4	MAPK14	Q16539	Kinase
Ginsenoside-Rh4	SIRT2	Q8IXJ6	Eraser

Ginsenoside-Rh4	MCHR1	Q99705	Family A G protein-coupled receptor
Ginsenoside-Rh4	TYMS	P04818	Transferase
Ginsenoside-Rh4	MAOA	P21397	Oxidoreductase
Ginsenoside-Rh4	MAPK1	P28482	Kinase
Ginsenoside-Rh4	HSD11B1	P28845	Enzyme
Ginsenoside-Rh4	SCD	O00767	Enzyme
Ginsenoside-Rh4	HTR1A	P08908	Family A G protein-coupled receptor
Ginsenoside-Rh4	TRPV4	Q9HBA0	Voltage-gated ion channel
Ginsenoside-Rh4	ADORA1	P30542	Family A G protein-coupled receptor
Ginsenoside-Rh4	PIM1	P11309	Kinase
Ginsenoside-Rh4	CDK2	P24941	Kinase
Ginsenoside-Rh4	PIM2	Q9P1W9	Kinase
Ginsenoside-Rh4	HDAC6	Q9UBN7	Eraser
Ginsenoside-Rh4	HDAC1	Q13547	Eraser
Ginsenoside-Rh4	EGFR	P00533	Kinase
Ginsenoside-Rh4	PDE5A	O76074	Phosphodiesterase
Ginsenoside-Rh4	CDK2 CCNA1 CCNA2	P24941 P78396 P20248	Other cytosolic protein
Ginsenoside-Rh4	PDE6A	P16499	Phosphodiesterase
Ginsenoside-Rh4	CCR1	P32246	Family A G protein-coupled receptor
Ginsenoside-Rh4	IRAK4	Q9NWZ3	Kinase
Ginsenoside-Rh4	CSNK2A1	P68400	Kinase
Ginsenoside-Rh4	CSNK2A2	P19784	Kinase
Ginsenoside-Rh4	PTGES	O14684	Enzyme
Ginsenoside-Rh4	SYK	P43405	Kinase
Ginsenoside-Rh4	SIGMAR1	Q99720	Membrane receptor
Ginsenoside-Rh4	PGK1	P00558	Enzyme
Ginsenoside-Rh4	PIK3CB	P42338	Enzyme
Ginsenoside-Rh4	ADORA3	P0DMS8	Family A G protein-coupled receptor
Ginsenoside-Rh4	REN	P00797	Protease
Ginsenoside-Rh4	RASGRP3	Q8IV61	Other cytosolic protein
Ginsenoside-Rh4	ATP1A1	P05023	Primary active transporter
Ginsenoside-Rh4	PIK3CG	P48736	Enzyme
Ginsenoside-Rh4	SLC5A2	P31639	Electrochemical transporter

Ginsenoside-Rh4	PDPK1	O15530	Kinase
Ginsenoside-Rh4	LIMK2	P53671	Kinase
Ginsenoside-Rh4	SMO	Q99835	Frizzled family G protein-coupled receptor
Ginsenoside-Rh4	AGTR1	P30556	Family A G protein-coupled receptor
Ginsenoside-Rh4	S1PR1	P21453	Family A G protein-coupled receptor
Ginsenoside-Rh4	AURKA	O14965	Kinase
Ginsenoside-Rh4	AURKB	Q96GD4	Kinase
Ginsenoside-Rh4	SLC6A4	P31645	Electrochemical transporter
Ginsenoside-Rh4	SLC6A3	Q01959	Electrochemical transporter
Ginsenoside-Rh4	NR4A2	P43354	Nuclear receptor
Ginsenoside-Rh4	MAPK8	P45983	Kinase
Ginsenoside-Rh4	SGK1	O00141	Kinase
Ginsenoside-Rh4	NTRK1	P04629	Kinase
Ginsenoside-Rh4	F10	P00742	Protease
Ginsenoside-Rh4	SLC5A1	P13866	Electrochemical transporter
Ginsenoside-Rh4	CHEK2	O96017	Kinase
Ginsenoside-Rh4	CASP1	P29466	Protease
Ginsenoside-Rh4	UPP1	Q16831	Enzyme
Ginsenoside-Rh4	HDAC3	O15379	Eraser
Ginsenoside-Rh4	TBXAS1	P24557	Cytochrome P450
Ginsenoside-Rh4	AKR1B1	P15121	Enzyme
Ginsenoside-Rh4	MAOB	P27338	Oxidoreductase
Ginsenoside-Rh4	JAK3	P52333	Kinase
Ginsenoside-Rh4	PPARG	P37231	Nuclear receptor
Ginsenoside-Rh4	JAK1	P23458	Kinase
Ginsenoside-Rh4	CYP2C9	P11712	Cytochrome P450
Ginsenoside-Rh4	MAP2K1	Q02750	Kinase
Ginsenoside-Rh4	S1PR3	Q99500	Family A G protein-coupled receptor
Ginsenoside-Rh4	SLC8A1	P32418	Electrochemical transporter
Ginsenoside-Rh4	ABCB11	O95342	Primary active transporter
Ginsenoside-Rh4	CNR1	P21554	Family A G protein-coupled receptor
Ginsenoside-Rh4	GAK	O14976	Kinase
Ginsenoside-Rh4	VEGFA	P15692	Secreted protein

Ginsenoside-Rh4	FGF1	P05230	Secreted protein
Ginsenoside-Rh4	FGF2	P09038	Secreted protein
Ginsenoside-Rh4	MKNK2	Q9HBH9	Kinase
Ginsenoside-Rh4	EPHB4	P54760	Kinase
Ginsenoside-Rh4	ITGB7 ITGA4	P26010 P13612	Membrane receptor
Ginsenoside-Rh4	PDE11A	Q9HCR9	Phosphodiesterase
Ginsenoside-Rh4	GYS1	P13807	Enzyme
Ginsenoside-Rh4	BCL2A1	Q16548	Unclassified protein
Ginsenoside-Rh4	RORC	P51449	Nuclear receptor
Ginsenoside-Rh4	PTGER1	P34995	Family A G protein-coupled receptor
Ginsenoside-Rh4	ADORA2A	P29274	Family A G protein-coupled receptor
Ginsenoside-Rh4	P2RX3	P56373	Ligand-gated ion channel
Girinimbin	DYRK1A	Q13627	Kinase
Girinimbin	BCHE	P06276	Hydrolase
Girinimbin	CLK4	Q9HAZ1	Kinase
Girinimbin	HTR2B	P41595	Family A G protein-coupled receptor
Girinimbin	HTR2C	P28335	Family A G protein-coupled receptor
Girinimbin	SLC6A3	Q01959	Electrochemical transporter
Girinimbin	HTR6	P50406	Family A G protein-coupled receptor
Girinimbin	AKT1	P31749	Kinase
Girinimbin	CLK2	P49760	Kinase
Girinimbin	DYRK3	O43781	Kinase
Girinimbin	SLC6A4	P31645	Electrochemical transporter
Girinimbin	SAE1 UBA2	Q9UBE0 Q9UBT2	Enzyme
Girinimbin	TACR1	P25103	Family A G protein-coupled receptor
Girinimbin	TACR3	P29371	Family A G protein-coupled receptor
Girinimbin	CDC25B	P30305	Phosphatase
Girinimbin	P2RX7	Q99572	Ligand-gated ion channel
Girinimbin	GRM2	Q14416	Family C G protein-coupled receptor
Girinimbin	HRH2	P25021	Family A G protein-coupled receptor
Girinimbin	HTR2A	P28223	Family A G protein-coupled receptor
Girinimbin	ADRB3	P13945	Family A G protein-coupled receptor
Girinimbin	ADORA2A	P29274	Family A G protein-coupled receptor

Girinimbin	ADORA3	P0DMS8	Family A G protein-coupled receptor
Girinimbin	OXTR	P30559	Family A G protein-coupled receptor
Girinimbin	CDK1	P06493	Kinase
Girinimbin	CDC25A	P30304	Phosphatase
Girinimbin	NPY5R	Q15761	Family A G protein-coupled receptor
Girinimbin	ADRA2A	P08913	Family A G protein-coupled receptor
Girinimbin	ADRA2C	P18825	Family A G protein-coupled receptor
Girinimbin	ADRA2B	P18089	Family A G protein-coupled receptor
Girinimbin	PDE5A	O76074	Phosphodiesterase
Girinimbin	KDR	P35968	Kinase
Girinimbin	HSD11B2	P80365	Enzyme
Girinimbin	HSD11B1	P28845	Enzyme
Girinimbin	TAAR1	Q96RJ0	Family A G protein-coupled receptor
Girinimbin	IDH1	O75874	Enzyme
Girinimbin	PPP1CA	P62136	Phosphatase
Girinimbin	METAP1	P53582	Protease
Girinimbin	LCK	P06239	Kinase
Girinimbin	PDE10A	Q9Y233	Phosphodiesterase
Girinimbin	CRHR1	P34998	Family B G protein-coupled receptor
Girinimbin	ABCC1	P33527	Primary active transporter
Girinimbin	CTRB1	P17538	Protease
Girinimbin	FAAH	O00519	Enzyme
Girinimbin	GPR55	Q9Y2T6	Family A G protein-coupled receptor
Girinimbin	FLT1	P17948	Kinase
Girinimbin	CNR1	P21554	Family A G protein-coupled receptor
Girinimbin	PIM1	P11309	Kinase
Girinimbin	AR	P10275	Nuclear receptor
Girinimbin	ERBB2	P04626	Kinase
Girinimbin	NR3C1	P04150	Nuclear receptor
Girinimbin	EGFR	P00533	Kinase
Girinimbin	ADORA1	P30542	Family A G protein-coupled receptor
Girinimbin	PFKFB3	Q16875	Enzyme
Girinimbin	MAPK14	Q16539	Kinase

Girinimbin	TSPO	P30536	Membrane receptor
Girinimbin	LIPE	Q05469	Enzyme
Girinimbin	QPCT	Q16769	Enzyme
Girinimbin	GABRB3 GABRA3 GABRG2	P28472 P34903 P18507	Ligand-gated ion channel
Girinimbin	GABRB3 GABRG2 GABRA1	P28472 P18507 P14867	Ligand-gated ion channel
Girinimbin	GABRB3 GABRG2 GABRA5	P28472 P18507 P31644	Ligand-gated ion channel
Girinimbin	GABRA2 GABRB3 GABRG2	P47869 P28472 P18507	Ligand-gated ion channel
	PSEN2 PSENEN NCSTN APH1A PSEN1	P49810 Q9NZ42 Q92542 Q96BI3 P49768	
Girinimbin	APH1B	Q8WW43	Protease
Girinimbin	CHRM3	P20309	Family A G protein-coupled receptor
Girinimbin	VCP	P55072	Primary active transporter
Girinimbin	NR1H4	Q96RI1	Nuclear receptor
Girinimbin	PGGT1B FNTA	P53609 P49354	Enzyme
Girinimbin	GABRG2 GABRB3 GABRA6	P18507 P28472 Q16445	Ligand-gated ion channel
Girinimbin	GABRB3 GABRA4 GABRG2	P28472 P48169 P18507	Ligand-gated ion channel
Girinimbin	PDGFRB	P09619	Kinase
Girinimbin	ADORA2B	P29275	Family A G protein-coupled receptor
Girinimbin	FGFR1	P11362	Kinase
Girinimbin	HCRTR2	O43614	Family A G protein-coupled receptor
Girinimbin	HCRTR1	O43613	Family A G protein-coupled receptor
Girinimbin	AVPR2	P30518	Family A G protein-coupled receptor
Girinimbin	APP	P05067	Membrane receptor
Girinimbin	EPHX1	P07099	Protease
Girinimbin	ELANE	P08246	Protease
Girinimbin	P2RY1	P47900	Family A G protein-coupled receptor
Girinimbin	DYRK2	Q92630	Kinase
Girinimbin	RET	P07949	Kinase
Girinimbin	EPHX2	P34913	Protease
Girinimbin	PTAFR	P25105	Family A G protein-coupled receptor
Girinimbin	ALK	Q9UM73	Kinase
Girinimbin	TRPV1	Q8NER1	Voltage-gated ion channel
Girinimbin	MDM2	Q00987	Other nuclear protein
Girinimbin	NR1H3	Q13133	Nuclear receptor

Girinimbin	NR1H2	P55055	Nuclear receptor
Girinimbin	PTGDR2	Q9Y5Y4	Family A G protein-coupled receptor
Girinimbin	CTSB	P07858	Protease
Girinimbin	HRH1	P35367	Family A G protein-coupled receptor
Girinimbin	HRH3	Q9Y5N1	Family A G protein-coupled receptor
Girinimbin	RORC	P51449	Nuclear receptor
Girinimbin	TYMS	P04818	Transferase
Girinimbin	RORB	Q92753	Nuclear receptor
Girinimbin	AURKA	O14965	Kinase
Girinimbin	RORA	P35398	Nuclear receptor
Girinimbin	GRIN1 GRIN2B	Q05586 Q13224	Ligand-gated ion channel
Girinimbin	HTR1A	P08908	Family A G protein-coupled receptor
Girinimbin	PDE7A	Q13946	Phosphodiesterase
Girinimbin	MGLL	Q99685	Enzyme
Gomisin B	PDE10A	Q9Y233	Phosphodiesterase
Gomisin B	CDK5R1 CDK5	Q15078 Q00535	Kinase
Gomisin B	KDR	P35968	Kinase
Gomisin B	CDK2	P24941	Kinase
Gomisin B	CDK1	P06493	Kinase
Gomisin B	HDAC6	Q9UBN7	Eraser
Gomisin B	HDAC1	Q13547	Eraser
Gomisin B	LIMK2	P53671	Kinase
Gomisin B	FKBP1A	P62942	Isomerase
Gomisin B	LTB4R	Q15722	Family A G protein-coupled receptor
Gomisin B	P2RX3	P56373	Ligand-gated ion channel
Gomisin B	EGFR	P00533	Kinase
Gomisin B	PDE2A	O00408	Phosphodiesterase
Gomisin B	NTRK1	P04629	Kinase
Gomisin B	JAK2	O60674	Kinase
Gomisin B	TLR4	O00206	Toll-like and Il-1 receptors
Gomisin B	CCND1 CDK4	P24385 P11802	Kinase
Gomisin B	CCNE2 CDK2 CCNE1	O96020 P24941 P24864	Other cytosolic protein
Gomisin B	ROS1	P08922	Kinase

Gomisin B	PDE5A	O76074	Phosphodiesterase
Gomisin B	GSK3B	P49841	Kinase
Gomisin B	PRKCB	P05771	Kinase
Gomisin B	BRPF1	P55201	Reader
Gomisin B	MAPK14	Q16539	Kinase
Gomisin B	HTR2B	P41595	Family A G protein-coupled receptor
Gomisin B	ABL1	P00519	Kinase
Gomisin B	HTR2C	P28335	Family A G protein-coupled receptor
Gomisin B	MAPK8	P45983	Kinase
Gomisin B	NPY5R	Q15761	Family A G protein-coupled receptor
Gomisin B	ABCC9	O60706	Primary active transporter
Gomisin B	CFD	P00746	Protease
Gomisin B	MAP2K1	Q02750	Kinase
Gomisin B	KLK5	Q9Y337	Protease
Gomisin B	PIM1	P11309	Kinase
Gomisin B	MTOR	P42345	Kinase
Gomisin B	PIK3CA	P42336	Enzyme
Gomisin B	PIM2	Q9P1W9	Kinase
Gomisin B	PIM3	Q86V86	Kinase
	PSEN2 PSENEN NCSTN APH1A PSEN1	P49810 Q9NZ42 Q92542 Q96BI3 P49768	
Gomisin B	APH1B	Q8WW43	Protease
Gomisin B	CTSD	P07339	Protease
Gomisin B	GPR139	Q6DWJ6	Family A G protein-coupled receptor
Gomisin B	CDK2 CCNA1 CCNA2	P24941 P78396 P20248	Other cytosolic protein
Gomisin B	DYRK1A	Q13627	Kinase
Gomisin B	IGF1R	P08069	Kinase
Gomisin B	F2	P00734	Protease
Gomisin B	PTGS2	P35354	Oxidoreductase
Gomisin B	JAK3	P52333	Kinase
Gomisin B	MET	P08581	Kinase
Gomisin B	LRRK2	Q5S007	Kinase
Gomisin B	OPRL1	P41146	Family A G protein-coupled receptor
Gomisin B	OPRM1	P35372	Family A G protein-coupled receptor

Gomisin B	OPRK1	P41145	Family A G protein-coupled receptor
Gomisin B	CRHR1	P34998	Family B G protein-coupled receptor
Gomisin B	CA1	P00915	Lyase
Gomisin B	CA9	Q16790	Lyase
Gomisin B	CCR1	P32246	Family A G protein-coupled receptor
Gomisin B	MAPK10	P53779	Kinase
Gomisin B	MAPK1	P28482	Kinase
Gomisin B	CCNE1 CDK2	P24864 P24941	Kinase
Gomisin B	CDK7 CCNH	P50613 P51946	Other cytosolic protein
Gomisin B	CDK9 CCNT1	P50750 O60563	Other cytosolic protein
Gomisin B	SCN9A	Q15858	Voltage-gated ion channel
Gomisin B	ERBB2	P04626	Kinase
Gomisin B	NCOR2 HDAC3	Q9Y618 O15379	Eraser
Gomisin B	AURKA	O14965	Kinase
Gomisin B	PSEN1	P49768	Other ion channel
Gomisin B	TACR1	P25103	Family A G protein-coupled receptor
Gomisin B	CCKBR	P32239	Family A G protein-coupled receptor
Gomisin B	GCK	P35557	Enzyme
Gomisin B	MERTK	Q12866	Kinase
Gomisin B	TNF	P01375	Secreted protein
Gomisin B	CCKAR	P32238	Family A G protein-coupled receptor
Gomisin B	PIK3CG	P48736	Enzyme
Gomisin B	MAP3K20	Q9NYL2	Kinase
Gomisin B	MAPK11	Q15759	Kinase
Gomisin B	TGFBR2	P37173	Kinase
Gomisin B	TGFBR1	P36897	Kinase
Gomisin B	FLT3	P36888	Kinase
Gomisin B	PDE4B	Q07343	Phosphodiesterase
Gomisin B	PARP1	P09874	Enzyme
Gomisin B	HDAC4	P56524	Eraser
Gomisin B	RPS6KA2	Q15349	Kinase
Gomisin B	HMGCR	P04035	Oxidoreductase
Gomisin B	AR	P10275	Nuclear receptor

Gomisin B	PDE4D	Q08499	Phosphodiesterase
Gomisin B	HCRTR2	O43614	Family A G protein-coupled receptor
Gomisin B	TK1	P04183	Transferase
Gomisin B	ADAM17	P78536	Protease
Gomisin B	MKNK2	Q9HBH9	Kinase
Gomisin B	CHEK1	O14757	Kinase
Gomisin B	HCRTR1	O43613	Family A G protein-coupled receptor
Gomisin B	TUBB1	Q9H4B7	Structural protein
Gomisin B	F9	P00740	Protease
Gomisin B	CXCR2	P25025	Family A G protein-coupled receptor
Gomisin B	INSR	P06213	Kinase
Gomisin B	RET	P07949	Kinase
Gomisin B	CCNB3 CDK1 CCNB1 CCNB2	Q8WWL7 P06493 P14635 O95067	Other cytosolic protein
Gomisin B	ADORA2A	P29274	Family A G protein-coupled receptor
Gomisin B	ADORA3	P0DMS8	Family A G protein-coupled receptor
Gomisin B	SYK	P43405	Kinase
Panaxadiol	CYP19A1	P11511	Cytochrome P450
Panaxadiol	CYP51A1	Q16850	Cytochrome P450
Panaxadiol	SHH	Q15465	Unclassified protein
Panaxadiol	NOS2	P35228	Enzyme
Panaxadiol	APP	P05067	Membrane receptor
Panaxadiol	CCR1	P32246	Family A G protein-coupled receptor
Panaxadiol	HSD11B2	P80365	Enzyme
Panaxadiol	ALK	Q9UM73	Kinase
Panaxadiol	MDM2	Q00987	Other nuclear protein
Panaxadiol	SLC6A2	P23975	Electrochemical transporter
Panaxadiol	ADORA1	P30542	Family A G protein-coupled receptor
Panaxadiol	ADORA2A	P29274	Family A G protein-coupled receptor
Panaxadiol	MDM4	O15151	Unclassified protein
Panaxadiol	CNR1	P21554	Family A G protein-coupled receptor
Panaxadiol	GRM1	Q13255	Family C G protein-coupled receptor
Panaxadiol	CNR2	P34972	Family A G protein-coupled receptor
Panaxadiol	S1PR3	Q99500	Family A G protein-coupled receptor

Panaxadiol	S1PR1	P21453	Family A G protein-coupled receptor
Panaxadiol	TACR2	P21452	Family A G protein-coupled receptor
Panaxadiol	SLC6A3	Q01959	Electrochemical transporter
Panaxadiol	CRHR1	P34998	Family B G protein-coupled receptor
Panaxadiol	KCNH2	Q12809	Voltage-gated ion channel
Panaxadiol	HCRTR2	O43614	Family A G protein-coupled receptor
Panaxadiol	HCRTR1	O43613	Family A G protein-coupled receptor
Panaxadiol	AVPR1A	P37288	Family A G protein-coupled receptor
Panaxadiol	F10	P00742	Protease
Panaxadiol	IL6ST	P40189	Membrane receptor
Panaxadiol	AKR1C3	P42330	Enzyme
Panaxadiol	NR1I2	O75469	Nuclear receptor
Panaxadiol	RORC	P51449	Nuclear receptor
Panaxadiol	PFKFB3	Q16875	Enzyme
Panaxadiol	MAPK14	Q16539	Kinase
Panaxadiol	LSS	P48449	Enzyme
Panaxadiol	RORA	P35398	Nuclear receptor
Panaxadiol	NR3C1	P04150	Nuclear receptor
Panaxadiol	TBXA2R	P21731	Family A G protein-coupled receptor
Panaxadiol	CHRM2	P08172	Family A G protein-coupled receptor
Panaxadiol	CHRM1	P11229	Family A G protein-coupled receptor
Panaxadiol	PTGS2	P35354	Oxidoreductase
Panaxadiol	KDR	P35968	Kinase
Panaxadiol	BACE1	P56817	Protease
Panaxadiol	MTNR1A	P48039	Family A G protein-coupled receptor
Panaxadiol	MTNR1B	P49286	Family A G protein-coupled receptor
Panaxadiol	MAPK8	P45983	Kinase
Panaxadiol	PTGES	O14684	Enzyme
Panaxadiol	LIMK2	P53671	Kinase
Panaxadiol	RASGRP3	Q8IV61	Other cytosolic protein
Panaxadiol	DRD2	P14416	Family A G protein-coupled receptor
Panaxadiol	PIK3CB	P42338	Enzyme
Panaxadiol	MET	P08581	Kinase

Panaxadiol	TRPV1	Q8NER1	Voltage-gated ion channel
Panaxadiol	GRM2	Q14416	Family C G protein-coupled receptor
Panaxadiol	HPGDS	O60760	Transferase
Panaxadiol	GRIN2A GRIN1	Q12879 Q05586	Ligand-gated ion channel
Panaxadiol	PYGL	P06737	Enzyme
Panaxadiol	CDK2	P24941	Kinase
Panaxadiol	GRIA2	P42262	Ligand-gated ion channel
Panaxadiol	MAPK1	P28482	Kinase
Panaxadiol	PIK3C3	Q8NEB9	Enzyme
Panaxadiol	PTGS1	P23219	Oxidoreductase
Panaxadiol	PDE3A	Q14432	Phosphodiesterase
Panaxadiol	PDE3B	Q13370	Phosphodiesterase
Panaxadiol	PIK3CD	O00329	Enzyme
Panaxadiol	PIK3CG	P48736	Enzyme
Panaxadiol	PI4KB	Q9UBF8	Enzyme
Panaxadiol	PIK3CA	P42336	Enzyme
Panaxadiol	CCR2	P41597	Family A G protein-coupled receptor
Panaxadiol	PGGT1B	P53609	Enzyme
Panaxadiol	HMGCR	P04035	Oxidoreductase
Panaxadiol	ITGAL ICAM1 ITGB2	P20701 P05362 P05107	Membrane receptor
Panaxadiol	PDE2A	O00408	Phosphodiesterase
Panaxadiol	PDE4B	Q07343	Phosphodiesterase
Panaxadiol	SLC10A2	Q12908	Electrochemical transporter
Panaxadiol	ALOX5AP	P20292	Other cytosolic protein
Panaxadiol	ACKR3	P25106	Family A G protein-coupled receptor
Panaxadiol	OPRL1	P41146	Family A G protein-coupled receptor
Panaxadiol	FAAH	O00519	Enzyme
Panaxadiol	PDE10A	Q9Y233	Phosphodiesterase
suchilactone	DYRK1A	Q13627	Kinase
suchilactone	DYRK2	Q92630	Kinase
suchilactone	DYRK1B	Q9Y463	Kinase
suchilactone	MET	P08581	Kinase
suchilactone	PIK3CA PIK3R1	P42336 P27986	Enzyme

suchilactone	CLK4	Q9HAZ1	Kinase
suchilactone	TGFBR1	P36897	Kinase
suchilactone	CDC25A	P30304	Phosphatase
suchilactone	CDC25B	P30305	Phosphatase
suchilactone	CASP3	P42574	Protease
suchilactone	CASP7	P55210	Protease
suchilactone	CTSL	P07711	Protease
suchilactone	CLK1	P49759	Kinase
suchilactone	CDK2 CCNA1 CCNA2	P24941 P78396 P20248	Other cytosolic protein
suchilactone	FLT1	P17948	Kinase
suchilactone	CCND1 CDK4	P24385 P11802	Kinase
suchilactone	PDGFRB	P09619	Kinase
suchilactone	FLT4	P35916	Kinase
suchilactone	IGF1R	P08069	Kinase
suchilactone	FLT3	P36888	Kinase
suchilactone	PDGFRA	P16234	Kinase
suchilactone	PTK2	Q05397	Kinase
suchilactone	PLK4	O00444	Kinase
suchilactone	TEK	Q02763	Kinase
suchilactone	AURKA	O14965	Kinase
suchilactone	EPHB4	P54760	Kinase
suchilactone	PDE4B	Q07343	Phosphodiesterase
suchilactone	CYP17A1	P05093	Cytochrome P450
suchilactone	PDE2A	O00408	Phosphodiesterase
suchilactone	LRRK2	Q5S007	Kinase
suchilactone	GRK3	P35626	Kinase
suchilactone	RAF1	P04049	Kinase
suchilactone	CASP9	P55211	Protease
suchilactone	GRK2	P25098	Kinase
suchilactone	QPCT	Q16769	Enzyme
suchilactone	CASP2	P42575	Protease
suchilactone	GRK5	P34947	Kinase
suchilactone	TAAR1	Q96RJ0	Family A G protein-coupled receptor

suchilactone	ROCK2	O75116	Kinase
suchilactone	SCD	O00767	Enzyme
suchilactone	JAK3	P52333	Kinase
suchilactone	JAK1	P23458	Kinase
suchilactone	JAK2	O60674	Kinase
suchilactone	MAPK10	P53779	Kinase
suchilactone	PLK1	P53350	Kinase
suchilactone	CYP11B1	P15538	Cytochrome P450
suchilactone	NR3C2	P08235	Nuclear receptor
suchilactone	PDE11A	Q9HCR9	Phosphodiesterase
suchilactone	HSP90AB1	P08238	Other cytosolic protein
suchilactone	PRKCA	P17252	Kinase
suchilactone	TSPO	P30536	Membrane receptor
suchilactone	ATP4B ATP4A	P51164 P20648	Primary active transporter
suchilactone	PDE4A	P27815	Phosphodiesterase
suchilactone	ADORA3	P0DMS8	Family A G protein-coupled receptor
suchilactone	PDE4C	Q08493	Phosphodiesterase
suchilactone	ASAH1	Q13510	Enzyme
suchilactone	PIK3CA	P42336	Enzyme
suchilactone	QTRT1	Q9BXR0	Enzyme
suchilactone	DRD1	P21728	Family A G protein-coupled receptor
	PSEN2 PSENEN NCSTN APH1A PSEN1	P49810 Q9NZ42 Q92542 Q96BI3 P49768	
suchilactone	APH1B	Q8WW43	Protease
suchilactone	NTRK1	P04629	Kinase
suchilactone	GSK3A	P49840	Kinase
suchilactone	IRAK4	Q9NWZ3	Kinase
suchilactone	MDM2	Q00987	Other nuclear protein
suchilactone	BRAF	P15056	Kinase
suchilactone	TNNI3K	Q59H18	Kinase
suchilactone	PORCN	Q9H237	Enzyme
suchilactone	GRIA1	P42261	Ligand-gated ion channel
suchilactone	HRH3	Q9Y5N1	Family A G protein-coupled receptor
suchilactone	NPY5R	Q15761	Family A G protein-coupled receptor

suchilactone	MTNR1B	P49286	Family A G protein-coupled receptor
suchilactone	MAPKAPK2	P49137	Kinase
suchilactone	RPS6KA3	P51812	Kinase
suchilactone	PARP1	P09874	Enzyme
suchilactone	SIRT2	Q8IXJ6	Eraser
suchilactone	SLC9A1	P19634	Electrochemical transporter
suchilactone	CDC7	O00311	Kinase
suchilactone	PIM1	P11309	Kinase
suchilactone	SYK	P43405	Kinase
suchilactone	TYK2	P29597	Kinase
suchilactone	PIM2	Q9P1W9	Kinase
suchilactone	PIM3	Q86V86	Kinase
suchilactone	PTK2B	Q14289	Kinase
suchilactone	PPIA	P62937	Isomerase
suchilactone	ADRA1D	P25100	Family A G protein-coupled receptor
suchilactone	HTR2C	P28335	Family A G protein-coupled receptor
suchilactone	MAPK8	P45983	Kinase
suchilactone	TTK	P33981	Kinase
suchilactone	P2RX7	Q99572	Ligand-gated ion channel
suchilactone	GABRB3 GABRA3 GABRG2	P28472 P34903 P18507	Ligand-gated ion channel
suchilactone	GABRB3 GABRG2 GABRA1	P28472 P18507 P14867	Ligand-gated ion channel
suchilactone	GABRB3 GABRG2 GABRA5	P28472 P18507 P31644	Ligand-gated ion channel
suchilactone	GABRA2 GABRB3 GABRG2	P47869 P28472 P18507	Ligand-gated ion channel
suchilactone	CSNK1A1	P48729	Kinase
suchilactone	CSNK1D	P48730	Kinase
suchilactone	PI4KB	Q9UBF8	Enzyme
suchilactone	CHUK	O15111	Kinase
suchilactone	CTSB	P07858	Protease
suchilactone	CLK2	P49760	Kinase
suchilactone	HTR2A	P28223	Family A G protein-coupled receptor
alexandrin_qt	IL2	P60568	Secreted protein
alexandrin_qt	STAT3	P40763	Transcription factor
alexandrin_qt	S1PR3	Q99500	Family A G protein-coupled receptor

alexandrin_qt	S1PR1	P21453	Family A G protein-coupled receptor
	PSEN2 PSENEN NCSTN APH1A PSEN1	P49810 Q9NZ42 Q92542 Q96BI3 P49768	
alexandrin_qt	APH1B	Q8WW43	Protease
alexandrin_qt	S1PR5	Q9H228	Family A G protein-coupled receptor
alexandrin_qt	S1PR4	O95977	Family A G protein-coupled receptor
alexandrin_qt	RBP4	P02753	Secreted protein
alexandrin_qt	PTAFR	P25105	Family A G protein-coupled receptor
alexandrin_qt	PFKFB3	Q16875	Enzyme
alexandrin_qt	FLT1	P17948	Kinase
alexandrin_qt	MET	P08581	Kinase
alexandrin_qt	PPM1B	O75688	Phosphatase
alexandrin_qt	PPP1CC	P36873	Phosphatase
alexandrin_qt	PPP2CA	P67775	Phosphatase
alexandrin_qt	PPP2R5A	Q15172	Phosphatase
alexandrin_qt	ALOX5	P09917	Oxidoreductase
alexandrin_qt	HSD11B2	P80365	Enzyme
alexandrin_qt	VCP	P55072	Primary active transporter
alexandrin_qt	PTPN1	P18031	Phosphatase
alexandrin_qt	MAOA	P21397	Oxidoreductase
alexandrin_qt	ADORA1	P30542	Family A G protein-coupled receptor
alexandrin_qt	ADORA2B	P29275	Family A G protein-coupled receptor
alexandrin_qt	ADORA3	P0DMS8	Family A G protein-coupled receptor
alexandrin_qt	MAPK14	Q16539	Kinase
alexandrin_qt	CDC25B	P30305	Phosphatase
ginsenoside Rg5	STAT3	P40763	Transcription factor
ginsenoside Rg5	ALDH2	P05091	Oxidoreductase
ginsenoside Rg5	VEGFA	P15692	Secreted protein
ginsenoside Rg5	FGF1	P05230	Secreted protein
ginsenoside Rg5	FGF2	P09038	Secreted protein
ginsenoside Rg5	HPSE	Q9Y251	Enzyme
ginsenoside Rg5	HSP90AA1	P07900	Other cytosolic protein
ginsenoside Rg5	ATP1A1	P05023	Primary active transporter
ginsenoside Rg5	F2	P00734	Protease

ginsenoside Rg5	ADORA1	P30542	Family A G protein-coupled receptor
ginsenoside Rg5	MTOR	P42345	Kinase
ginsenoside Rg5	SLC5A2	P31639	Electrochemical transporter
ginsenoside Rg5	SLC5A1	P13866	Electrochemical transporter
ginsenoside Rg5	MAPK8	P45983	Kinase
ginsenoside Rg5	F7	P08709	Protease
ginsenoside Rg5	TACR2	P21452	Family A G protein-coupled receptor
ginsenoside Rg5	MMP9	P14780	Protease
ginsenoside Rg5	MAP2K1	Q02750	Kinase
ginsenoside Rg5	EGFR	P00533	Kinase
ginsenoside Rg5	S1PR3	Q99500	Family A G protein-coupled receptor
ginsenoside Rg5	S1PR1	P21453	Family A G protein-coupled receptor
ginsenoside Rg5	MAPK14	Q16539	Kinase
ginsenoside Rg5	PTPN1	P18031	Phosphatase
ginsenoside Rg5	ADORA2A	P29274	Family A G protein-coupled receptor
ginsenoside Rg5	SIRT2	Q8IXJ6	Eraser
ginsenoside Rg5	FCER2	P06734	Membrane receptor
ginsenoside Rg5	ADK	P55263	Enzyme
ginsenoside Rg5	MMP1	P03956	Protease
ginsenoside Rg5	PIK3CG	P48736	Enzyme
ginsenoside Rg5	SF3B3	Q15393	Unclassified protein
ginsenoside Rg5	EIF4A1	P60842	Hydrolase
ginsenoside Rg5	ADORA3	P0DMS8	Family A G protein-coupled receptor
ginsenoside Rg5	GABRA5	P31644	Ligand-gated ion channel
ginsenoside Rg5	LRRK2	Q5S007	Kinase
ginsenoside Rg5	PRF1	P14222	Other ion channel
ginsenoside Rg5	UGCG	Q16739	Transferase
ginsenoside Rg5	ADRB2	P07550	Family A G protein-coupled receptor
ginsenoside Rg5	ADRB1	P08588	Family A G protein-coupled receptor
ginsenoside Rg5	ADRB3	P13945	Family A G protein-coupled receptor
ginsenoside Rg5	GBA2	Q9HCG7	Enzyme
ginsenoside Rg5	FKBP1A	P62942	Isomerase
ginsenoside Rg5	MAPK11	Q15759	Kinase

ginsenoside Rg5	AGTR2	P50052	Family A G protein-coupled receptor
ginsenoside Rg5	PDE5A	O76074	Phosphodiesterase
ginsenoside Rg5	MMP13	P45452	Protease
ginsenoside Rg5	MMP2	P08253	Protease
ginsenoside Rg5	PDE6A	P16499	Phosphodiesterase
ginsenoside Rg5	MMP8	P22894	Protease
Fumarine	DRD2	P14416	Family A G protein-coupled receptor
Fumarine	DRD1	P21728	Family A G protein-coupled receptor
Fumarine	HTR1A	P08908	Family A G protein-coupled receptor
Fumarine	F3	P13726	Surface antigen
Fumarine	HTR7	P34969	Family A G protein-coupled receptor
Fumarine	CHRM4	P08173	Family A G protein-coupled receptor
Fumarine	SLC6A3	Q01959	Electrochemical transporter
Fumarine	ADRA1D	P25100	Family A G protein-coupled receptor
Fumarine	ADRA1A	P35348	Family A G protein-coupled receptor
Fumarine	DRD3	P35462	Family A G protein-coupled receptor
Fumarine	DRD4	P21917	Family A G protein-coupled receptor
Fumarine	CDK2 CCNA1 CCNA2	P24941 P78396 P20248	Other cytosolic protein
Fumarine	CDC7	O00311	Kinase
Fumarine	CHEK1	O14757	Kinase
Fumarine	SIGMAR1	Q99720	Membrane receptor
Fumarine	PARP2	Q9UGN5	Enzyme
Fumarine	PIM1	P11309	Kinase
Fumarine	PIM3	Q86V86	Kinase
Fumarine	TRHR	P34981	Family A G protein-coupled receptor
Fumarine	CYP19A1	P11511	Cytochrome P450
Fumarine	MAPK8	P45983	Kinase
Fumarine	JAK3	P52333	Kinase
Fumarine	JAK1	P23458	Kinase
Fumarine	JAK2	O60674	Kinase
Fumarine	TTK	P33981	Kinase
Fumarine	ADRA1B	P35368	Family A G protein-coupled receptor
Fumarine	MKNK2	Q9HBH9	Kinase

Fumarine	CCNE1 CDK2	P24864 P24941	Kinase
Fumarine	NEK1	Q96PY6	Kinase
Fumarine	LRRK2	Q5S007	Kinase
Fumarine	PDPK1	O15530	Kinase
Fumarine	TYMS	P04818	Transferase
Fumarine	APP	P05067	Membrane receptor
Fumarine	PIK3CD	O00329	Enzyme
Fumarine	PIK3CB	P42338	Enzyme
Fumarine	PIK3CA	P42336	Enzyme
Fumarine	DUSP3	P51452	Phosphatase
Fumarine	ERN1	O75460	Enzyme
Fumarine	PTGS2	P35354	Oxidoreductase
Fumarine	DRD5	P21918	Family A G protein-coupled receptor
ginsenoside Ra1	STAT3	P40763	Transcription factor
ginsenoside Ra1	PTAFR	P25105	Family A G protein-coupled receptor
ginsenoside Ra1	IL2	P60568	Secreted protein
ginsenoside Ra1	VEGFA	P15692	Secreted protein
ginsenoside Ra1	FGF1	P05230	Secreted protein
ginsenoside Ra1	FGF2	P09038	Secreted protein
ginsenoside Ra1	HPSE	Q9Y251	Enzyme
ginsenoside Ra1	RORC	P51449	Nuclear receptor
ginsenoside Ra1	ATP1A1	P05023	Primary active transporter
	PSEN2 PSENEN NCSTN APH1A PSEN1	P49810 Q9NZ42 Q92542 Q96BI3 P49768	
ginsenoside Ra1	APH1B	Q8WW43	Protease
ginsenoside Ra1	HSP90AA1	P07900	Other cytosolic protein
ginsenoside Ra1	LGALS4	P56470	Other cytosolic protein
ginsenoside Ra1	LGALS8	O00214	Other cytosolic protein
ginsenoside Ra1	BCL2L1	Q07817	Other ion channel
ginsenoside Ra1	CDK1	P06493	Kinase
ginsenoside Ra1	HSD11B2	P80365	Enzyme
ginsenoside Ra1	HSD11B1	P28845	Enzyme
ginsenoside Ra3	STAT3	P40763	Transcription factor
ginsenoside Ra3	PTAFR	P25105	Family A G protein-coupled receptor

ginsenoside Ra3	VEGFA	P15692	Secreted protein
ginsenoside Ra3	FGF1	P05230	Secreted protein
ginsenoside Ra3	FGF2	P09038	Secreted protein
ginsenoside Ra3	HPSE	Q9Y251	Enzyme
ginsenoside Ra3	IL2	P60568	Secreted protein
	PSEN2 PSENEN NCSTN APH1A PSEN1	P49810 Q9NZ42 Q92542 Q96BI3 P49768	
ginsenoside Ra3	APH1B	Q8WW43	Protease
ginsenoside Ra3	BCL2L1	Q07817	Other ion channel
ginsenoside Ra3	HSP90AA1	P07900	Other cytosolic protein
ginsenoside Ra3	LGALS4	P56470	Other cytosolic protein
ginsenoside Ra3	LGALS8	O00214	Other cytosolic protein
ginsenoside Ra3	CDK1	P06493	Kinase
ginsenoside Ra3	RORC	P51449	Nuclear receptor
ginsenoside Rc	STAT3	P40763	Transcription factor
ginsenoside Rc	PTAFR	P25105	Family A G protein-coupled receptor
ginsenoside Rc	IL2	P60568	Secreted protein
ginsenoside Rc	VEGFA	P15692	Secreted protein
ginsenoside Rc	FGF1	P05230	Secreted protein
ginsenoside Rc	FGF2	P09038	Secreted protein
ginsenoside Rc	HPSE	Q9Y251	Enzyme
ginsenoside Rc	RORC	P51449	Nuclear receptor
ginsenoside Rc	HSP90AA1	P07900	Other cytosolic protein
	PSEN2 PSENEN NCSTN APH1A PSEN1	P49810 Q9NZ42 Q92542 Q96BI3 P49768	
ginsenoside Rc	APH1B	Q8WW43	Protease
ginsenoside Rc	CDK1	P06493	Kinase
ginsenoside Rc	LGALS4	P56470	Other cytosolic protein
ginsenoside Rc	LGALS3	P17931	Other cytosolic protein
ginsenoside Rc	LGALS8	O00214	Other cytosolic protein
ginsenoside Rc	BCL2L1	Q07817	Other ion channel
ginsenoside Rc	HSD11B2	P80365	Enzyme
ginsenoside Rc	HSD11B1	P28845	Enzyme
notoginsenoside Fe	PTAFR	P25105	Family A G protein-coupled receptor
notoginsenoside Fe	IL2	P60568	Secreted protein

notoginsenoside Fe	STAT3	P40763	Transcription factor
notoginsenoside Fe	VEGFA	P15692	Secreted protein
notoginsenoside Fe	FGF1	P05230	Secreted protein
notoginsenoside Fe	FGF2	P09038	Secreted protein
notoginsenoside Fe	HPSE	Q9Y251	Enzyme
notoginsenoside Fe	RORC	P51449	Nuclear receptor
notoginsenoside Fe	ATP1A1	P05023	Primary active transporter
notoginsenoside Fe	HSP90AA1	P07900	Other cytosolic protein
	PSEN2 PSENEN NCSTN APH1A PSEN1	P49810 Q9NZ42 Q92542 Q96BI3 P49768	
notoginsenoside Fe	APH1B	Q8WW43	Protease
notoginsenoside Fe	CDK1	P06493	Kinase
notoginsenoside Fe	LGALS4	P56470	Other cytosolic protein
notoginsenoside Fe	LGALS3	P17931	Other cytosolic protein
notoginsenoside Fe	LGALS8	O00214	Other cytosolic protein
notoginsenoside Fe	BCL2L1	Q07817	Other ion channel
notoginsenoside Fe	HSD11B2	P80365	Enzyme
notoginsenoside Fe	HSD11B1	P28845	Enzyme

Table5 All target proteins of ginseng active ingredients

HMGCR	HSD17B3	NEK2	REN	LDHA	MGLL	STAT3	CA2	KCNH2	CDK2 CCNA1 CCNA2	MAOB	CASP7
NR1H3	HSD17B2	CAMK2B	PAK4	ACE	FKBP1A	IL2	ALOX5	F10	PDPK1	CYP2C9	FLT4
NPC1L1	MTOR	ALK	BDKRB1	ITGAL ICAM1 ITGB2	TLR4	HSP90AA1	CA7	ITGA2B ITGB3	TYK2	MAP2K1	PDGFRA
CYP17A1	PIK3CD	AKT1	GHSR	AGTR1	CCND1 CDK4	PTAFR	ABCC1	PGGT1B FNTA	CTSV	S1PR3	PLK4
AR	PIK3CB	NEK6	FGFR1	HNF4A	CCNE2	PSEN2	HSD17B1	SIGMAR	CTSL	SLC8A1	TEK

					CDK2 CCNE1	PSENEN NCSTN APH1A PSEN1 APH1B		1			
SREBF2	PIK3CG	PLA2G1 B	CDK9	TRPV1	ROS1	VEGFA	AHR	ACACB	FOLH1	ABCB11	GRK3
CYP19A1	PIK3CA	CA5A	CNR2	KDM2A	BRPF1	FGF1	CA12	PSMB5	FABP4	GAK	CASP9
RORC	PGF	BACE1	CASP8	KDM5C	ABCC9	FGF2	CYP1B1	MLYCD	PPARG	MKNK2	CASP2
ESR1	F3	AXL	CASP1	PLA2G10	KLK5	HPSE	ABCG2	CHRM3	PPARA	ITGB7 ITGA4	GRK5
ESR2	TUBB1	NUAK1	UGCG	DHODH	GPR139	ATP1A1	CA4	F7	FFAR1	BCL2A1	ROCK2
SHBG	TUBB3	AKR1C2	XIAP	EDNRA	LRRK2	BCL2L1	MAOA	UTS2R	FABP3	P2RX3	CYP11B1
SLC6A2	CCND3 CCND1 CDK4 CCND2	AKR1C1	BIRC2	PDE4B	OPRL1	S1PR1	GLO1	PDE10A	PTGS1	DYRK1A	NR3C2
SERPINA 6	ABL1	AKR1C3	CFD	BMP1	CCNE1 CDK2	LGALS4	GSK3B	CCR1	CNR1	CLK4	HSP90AB 1
PTPN1	RAF1	AKR1C4	CTSD	CCKBR	CDK7 CCNH	LGALS3	MMP9	AVPR1A	FAAH	HTR2B	ATP4B ATP4A
CHRM2	JAK3	CA13	PDE11A	CMA1	CDK9 CCNT1	LGALS8	MMP2	MAPK14	FABP5	CLK2	ASAH1
RORA	LCK	AKR1A1	KIF11	CTSG	NCOR2 HDAC3	TACR2	PTPRS	NTRK1	FABP1	DYRK3	QTRT1
BCHE	LNPEP	APP	ERBB2	PSEN1	GCK	ADORA1	ADORA2 A	TRPV3	PTGES	SAE1 UBA2	DRD1
SLC6A4	CDK2	MMP12	HTR4	SOAT1	MERTK	SYK	CDK5R1 CDK5	CTSK	SCD	TACR3	TNNI3K
ACHE	PARP1	CD38	HRH3	SOAT2	TNF	CDK1	CCNB3 CDK1 CCNB1	CTSS	CYP26B1	HRH2	PORCN

							CCNB2				
CYP2C19	PRKDC	TOP1	CTSE	F2R	CCKAR	CES1	ARG1	BAZ2B	CYP26A1	OXTR	GRIA1
G6PD	HCK	PTGS2	MDM2	NFKB1	MAP3K20	ALOX15	GPR35	CECR2	PTGDR2	NPY5R	SLC9A1
CDC25A	PI4KB	CFTR	MCHR1	CA5B	MAPK11	MAPKAPK 2	DAPK1	BAZ2A	PTPN11	TAAR1	CDC7
CES2	PIM2	PFKFB3	GSK3A	PIK3CD PIK3R1	TGFBR2	CSNK1G1	MPG	CYP11B2	RXRA	IDH1	PTK2B
NR1H2	BRAF	AMY1A	PLAT	AGPAT2	TGFBR1	RPS6KA1	SLC22A12	JAK1	RARG	PPP1CA	PPIA
VDR	EPHB4	GRK6	HRH4	TRAP1	RPS6KA2	ROCK1	AKR1B10	JAK2	RARB	METAP1	TTK
PTGER1	ABCB1	TERT	BRD4	HSP90B1	TK1	MAPK1	TNKS2	PDE9A	RARA	CTRB1	CSNK1A1
PTGER2	MIF	MAPT	HTR2C	ALPL	F9	ERN1	TNKS	AURKA	RXRB	GPR55	CSNK1D
HSD11B1	MMP8	HTR2A	RPS6KB1	MAPK8	CXCR2	RET	CDK6	L3MBTL3	RXRG	FLT1	CHUK
PPARD	CXCR1	OPRK1	BACE2	MAPK9	INSR	RPS6KA3	CSNK2A1	CRHR1	PTGER4	TSPO	S1PR5
GLRA1	PIM3	OPRM1	ADRA2A	PRF1	MDM4	EZR	EGFR	PRKCQ	FABP2	LIPE	S1PR4
SQLE	TBK1	CCR5	ADRA2C	SIRT2	GRM1	ESRRA	AVPR2	GPBAR1	PTGIR	QPCT	PPM1B
NR1I3	IMPDH1	EPHX2	ADRA2B	HTR1A	IL6ST	ESRRB	IGF1R	L3MBTL1	ENPP2	GABRB3 GABRA3 GABRG2	PPP1CC
DHCR7	TYMS	SLC6A3	CACNA2D1	TRPV4	NR1I2	DAO	F2	CHRM1	PTGES2	GABRB3 GABRG2 GABRA1	PPP2CA
PTPN6	IMPDH2	CTSB	FNTA FNTB	HDAC6	LSS	GRM4	PIM1	MAPK10	LTB4R	GABRB3 GABRG2 GABRA5	PPP2R5A
NR3C1	GUSB	ADRA1D	PLAU	HDAC1	TBXA2R	CLK1	AURKB	PDE4D	PRKCH	GABRA2 GABRB3 GABRG2	ALDH2
TBXAS1	CA14	ADRA1A	PDE8B	PDE6A	MTNR1A	DYRK1B	DRD4	PDE4C	GRM2	VCP	FCER2
GCGR	ADAM17	ADRA1B	ADRB2	IRAK4	MTNR1B	HDAC2	MPO	PARP3	NR0B2	NR1H4	ADK
NOS2	ADCY5	CHRNA4 CHRNB2	ADRB1	CSNK2A2	HPGDS	TRPM8	PIK3R1	CREBBP	PLA2G4A	GABRG2 GABRB3 GABRA6	MMP1

HSD11B2	MAP3K8	OPRD1	ADRB3	PGK1	GRIN2A<br>GRIN1	TTR	PYGL	PARP4	FFAR4	GABRB3<br>GABRA4<br>GABRG2	SF3B3
POLB	SRC	PRCP	ELANE	ADORA3	GRIA2	DGAT1	CA1	YES1	DAGLA	PDGFRB	EIF4A1
PRKCD	COMT	CAPN1	PRKCG	RASGRP3	PIK3C3	CHEK2	PTK2	PLA2G2A	TRPA1	HCRTR2	GABRA5
PTPN2	ADORA2B	DPP4	PRKCA	SLC5A2	PDE3A	ALOX12	MMP13	SLC6A9	OXER1	HCRTR1	GBA2
KCNK2	HDAC5	TACR1	PRKCB	LIMK2	PDE3B	KDR	MMP3	CHRM4	SLC16A1	EPHX1	AGTR2
CYP51A1	HDAC7	THRB	PRKCE	SMO	CCR2	HDAC4	CA3	PDE2A	PDE4A	P2RY1	HTR7
FDFT1	NOX4	P2RX7	HTR1D	NR4A2	PGGT1B	GRK2	PLK1	CALCRL	DAGLB	DYRK2	PARP2
CDC25B	AKR1B1	CCR3	ATAD2	SGK1	SLC10A2	ALPG	CA6	PDE5A	GCG	HRH1	TRHR
SHH	XDH	DRD3	HTR6	SLC5A1	ACKR3	PLAA	PKN1	CHRNB1<br>CHRNA1<br>CHRNG<br>CHRND	GYS1	RORB	NEK1
UGT2B7	TYR	MC4R	SCN9A	UPP1	PIK3CA<br>PIK3R1	CHEK1	CA9	H1F0	ALOX5AP	GRIN1<br>GRIN2B	DUSP3
DRD2	FLT3	EP300	MAP3K12	HDAC3	CASP3	WEE1	MET	NTRK2	RBP4	PDE7A	DRD5

Table 6 Degree unDir, Betweenness unDir, Closeness unDir analyzed by CentiScaPe 2.2 for protein interactions in CHF disease modules

Betweenness unDir	Closeness unDir	Degree unDir	name	selected	shared name
91.72983	9.24E-04	10	A2M	TRUE	A2M
11679.44	0.001299	95	AKT1	FALSE	AKT1
7490.024	0.001255	93	IL6	FALSE	IL6
14217.41	0.001272	92	TP53	FALSE	TP53
5938.163	0.001252	85	STAT3	FALSE	STAT3
38.34685	8.55E-04	8	ELANE	TRUE	ELANE

10438.09	0.001256	85	EGFR	FALSE	EGFR
12555.53	0.001266	82	ACTB	FALSE	ACTB
13.49837	8.21E-04	7	SERPINC1	TRUE	SERPINC1
4078.203	0.001211	77	TNF	FALSE	TNF
11005.79	0.001241	71	GAPDH	FALSE	GAPDH
1465.764	8.38E-04	10	ACACA	FALSE	ACACA
0	6.27E-04	1	PPAT	TRUE	PPAT
433.2189	8.08E-04	12	DLAT	TRUE	DLAT
3605.283	0.001209	70	IL1B	FALSE	IL1B
6473.795	0.001211	66	CTNNB1	FALSE	CTNNB1
5540.525	0.001203	65	INS	FALSE	INS
2910.342	0.001167	58	MMP9	FALSE	MMP9
0	6.93E-04	3	ELOVL6	TRUE	ELOVL6
2638.028	0.001135	57	IFNG	FALSE	IFNG
0	6.47E-04	2	ALDH6A1	TRUE	ALDH6A1
3282.294	0.001147	57	TLR4	FALSE	TLR4
6002.806	0.001172	57	MAPK3	FALSE	MAPK3
2732.326	0.001167	54	MYC	FALSE	MYC
4337.157	0.00116	53	ALB	FALSE	ALB
26.48076	8.43E-04	8	XDH	TRUE	XDH
1737.116	0.001126	53	EGF	FALSE	EGF
1608.388	0.001168	50	NFKB1	FALSE	NFKB1
17.73605	9.05E-04	15	SELE	FALSE	SELE
1035.603	0.001099	49	CCL2	FALSE	CCL2
137.3744	7.92E-04	8	NPPB	TRUE	NPPB
548.3597	8.75E-04	13	NPPA	TRUE	NPPA
5039.945	0.001139	48	PPARG	FALSE	PPARG
2619.634	0.001116	47	CD4	FALSE	CD4
0.70936	7.60E-04	4	NR3C2	TRUE	NR3C2
1806.277	0.001109	47	IGF1	FALSE	IGF1
1819.061	0.001134	46	HIF1A	FALSE	HIF1A
1079.08	0.00108	46	ICAM1	FALSE	ICAM1
2451.803	0.001121	45	FOS	FALSE	FOS

5993.238	0.001037	41	AGT	FALSE	AGT
4279.124	0.001098	40	HSPA8	FALSE	HSPA8
0	7.19E-04	2	MEP1A	TRUE	MEP1A
2480.607	0.00106	39	LEP	FALSE	LEP
1964.797	0.001115	39	NFKBIA	FALSE	NFKBIA
3311.642	0.001116	39	CAV1	FALSE	CAV1
1107.368	0.001078	39	JAK2	FALSE	JAK2
170.9905	8.69E-04	9	PCK1	TRUE	PCK1
0	7.14E-04	4	HMGCS2	TRUE	HMGCS2
2043.542	9.94E-04	38	CRP	FALSE	CRP
738.4317	0.001101	38	FGF2	FALSE	FGF2
0	6.35E-04	1	ACOT2	TRUE	ACOT2
2285.695	8.51E-04	9	PFKM	FALSE	PFKM
1747.052	0.001114	37	SIRT1	FALSE	SIRT1
1471.286	0.001107	37	PTEN	FALSE	PTEN
19.42539	6.60E-04	2	CKM	TRUE	CKM
11.05056	7.76E-04	3	ACTA2	TRUE	ACTA2
472.9245	8.60E-04	10	TNNI3	TRUE	TNNI3
3644.497	0.001079	37	HDAC1	FALSE	HDAC1
1046.47	0.001016	37	CSF3	FALSE	CSF3
87.72393	8.50E-04	3	CAPZB	TRUE	CAPZB
127.1875	7.65E-04	8	TNNT2	TRUE	TNNT2
1021.434	0.001105	36	PTGS2	FALSE	PTGS2
183.0736	0.001034	36	IL1A	FALSE	IL1A
578.5002	0.001017	36	VCAM1	FALSE	VCAM1
5125.346	0.001117	35	APP	FALSE	APP
1357.903	0.001053	34	ITGB1	FALSE	ITGB1
2012.416	0.001066	34	GRB2	FALSE	GRB2
63.69062	9.25E-04	8	DDX5	TRUE	DDX5
2624.295	0.001105	33	HSPA5	FALSE	HSPA5
588.8673	0.001089	33	CCND1	FALSE	CCND1
1056.853	0.001087	33	CDH1	FALSE	CDH1
2653.757	0.001053	33	SMAD4	FALSE	SMAD4

759.9762	0.00105	33	KDR	FALSE	KDR
581.4809	0.001022	32	SERPINE1	FALSE	SERPINE1
1913.607	0.001073	32	STAT5A	FALSE	STAT5A
1915.057	0.001088	32	MAPK8	FALSE	MAPK8
221.2302	0.001013	31	CSF2	FALSE	CSF2
37.57972	0.001005	17	CDH2	FALSE	CDH2
3764.068	0.001063	30	PPARGC1A	FALSE	PPARGC1A
1346.895	0.001073	30	GSK3B	FALSE	GSK3B
15.90136	8.62E-04	4	RHOC	TRUE	RHOC
1752.855	0.00106	29	SMAD2	FALSE	SMAD2
2407.417	0.001071	29	NFE2L2	FALSE	NFE2L2
963.4441	9.85E-04	29	CXCL10	FALSE	CXCL10
1914.92	0.001065	29	MAPK14	FALSE	MAPK14
1494.12	0.001012	27	ADIPOQ	FALSE	ADIPOQ
797.3224	9.81E-04	27	EDN1	FALSE	EDN1
447.3276	9.58E-04	27	VWF	FALSE	VWF
2269.021	0.001025	26	NOS3	FALSE	NOS3
1764.735	9.85E-04	25	SQSTM1	FALSE	SQSTM1
549.9734	0.001055	25	MDM2	FALSE	MDM2
327.2151	0.001024	25	SHC1	FALSE	SHC1
774.6616	0.00103	24	CCN2	FALSE	CCN2
2366.291	0.001025	23	HSPA9	FALSE	HSPA9
4.629894	8.61E-04	5	H2BC7	TRUE	H2BC7
2634.263	0.001026	23	VCP	FALSE	VCP
1996.443	0.001011	23	CALR	FALSE	CALR
733.6311	9.73E-04	23	CD36	FALSE	CD36
1978.162	9.57E-04	23	POMC	FALSE	POMC
2684.524	9.74E-04	23	SOD1	FALSE	SOD1
620.8482	9.47E-04	22	REN	FALSE	REN
847.4377	0.001042	22	HMOX1	FALSE	HMOX1
549.7577	0.001026	22	EZH2	FALSE	EZH2
191.2988	0.001015	22	TNFRSF1A	FALSE	TNFRSF1A
134.7807	9.57E-04	22	CCL3	FALSE	CCL3

686.5822	0.001017	21	YWHAZ	FALSE	YWHAZ
139.0726	0.001007	21	SOCS3	FALSE	SOCS3
1126.744	9.11E-04	21	UBA52	FALSE	UBA52
1800.705	9.66E-04	21	CDK1	FALSE	CDK1
107.235	8.96E-04	4	KAT8	TRUE	KAT8
602.1593	9.90E-04	20	ACE	FALSE	ACE
1688.476	9.22E-04	20	AGTR1	FALSE	AGTR1
1348.107	9.89E-04	20	VCL	FALSE	VCL
258.3997	8.53E-04	5	TUBA1C	TRUE	TUBA1C
608.2295	0.001028	20	CYBB	FALSE	CYBB
48.26402	8.99E-04	7	H4C15	TRUE	H4C15
2166.883	9.95E-04	20	ARRB1	FALSE	ARRB1
4.629894	8.61E-04	5	H2BC6	TRUE	H2BC6
2771.642	9.98E-04	20	PPP2CA	FALSE	PPP2CA
220.4196	9.90E-04	20	KITLG	FALSE	KITLG
2164.139	9.92E-04	20	BTRC	FALSE	BTRC
839.4088	9.81E-04	19	ACE2	FALSE	ACE2
3.250568	7.61E-04	7	ACVRL1	TRUE	ACVRL1
55.44057	8.37E-04	7	SMAD1	TRUE	SMAD1
165.8299	9.76E-04	19	ITGB3	FALSE	ITGB3
283.0652	7.89E-04	10	BMPR2	TRUE	BMPR2
91.89067	8.43E-04	9	SMAD9	TRUE	SMAD9
9.186233	7.76E-04	7	BMPR1B	TRUE	BMPR1B
1.762613	7.60E-04	6	GDF2	TRUE	GDF2
3509.506	0.001026	19	ENO1	FALSE	ENO1
148.5662	8.67E-04	10	UCP1	TRUE	UCP1
2.044949	8.39E-04	2	CDH13	TRUE	CDH13
6.369534	8.20E-04	5	CIDEA	TRUE	CIDEA
231.7839	9.76E-04	19	NTRK1	FALSE	NTRK1
68.61436	8.07E-04	6	ADRB3	TRUE	ADRB3
25.33004	8.32E-04	6	RBP4	TRUE	RBP4
213.3922	9.35E-04	19	CCR5	FALSE	CCR5
920.8422	9.42E-04	19	IRF1	FALSE	IRF1

128.1924	9.54E-04	18	SYK	FALSE	SYK
0	7.58E-04	3	CFD	TRUE	CFD
12.7976	7.36E-04	2	ADRA2C	TRUE	ADRA2C
73.39861	7.01E-04	7	GNA12	TRUE	GNA12
160.734	7.90E-04	8	ADRB1	TRUE	ADRB1
1556.543	8.96E-04	17	CS	FALSE	CS
46.21753	7.47E-04	4	CAMK2D	TRUE	CAMK2D
405.3015	9.46E-04	17	ACTG1	FALSE	ACTG1
945.1517	9.73E-04	17	GCG	FALSE	GCG
51.57476	7.87E-04	7	AVPR2	TRUE	AVPR2
255.3734	9.70E-04	17	LGALS3	FALSE	LGALS3
111.8377	8.95E-04	7	CAV3	TRUE	CAV3
195.841	0.001009	17	HMGB1	FALSE	HMGB1
70.63225	8.67E-04	7	NOX4	TRUE	NOX4
1565.53	9.64E-04	17	SOD2	FALSE	SOD2
25.68793	7.56E-04	4	EDNRA	TRUE	EDNRA
538.2213	7.69E-04	4	RXFP3	TRUE	RXFP3
273.1471	7.34E-04	3	RLN3	TRUE	RLN3
329.4469	9.59E-04	16	LRRK2	FALSE	LRRK2
12.31656	7.78E-04	7	AVP	TRUE	AVP
121.9485	8.04E-04	8	EDNRB	TRUE	EDNRB
0	7.33E-04	1	MAS1	TRUE	MAS1
0	6.73E-04	1	HTR2B	TRUE	HTR2B
312.2177	9.66E-04	16	EZR	FALSE	EZR
218.6789	9.57E-04	16	MSN	FALSE	MSN
0	5.65E-04	1	AK1	TRUE	AK1
961.1315	7.30E-04	3	PGM1	FALSE	PGM1
1146.511	9.35E-04	16	YWHAB	FALSE	YWHAB
56.33684	9.92E-04	21	HGF	FALSE	HGF
6.44413	8.58E-04	3	ARHGDIB	TRUE	ARHGDIB
302.9923	0.001018	16	TFRC	FALSE	TFRC
871.776	9.91E-04	16	NPM1	FALSE	NPM1
478.1419	8.98E-04	16	HDAC4	FALSE	HDAC4

140.2072	9.68E-04	16	CD9	FALSE	CD9
31.99923	9.07E-04	4	MAP3K8	TRUE	MAP3K8
386.534	9.26E-04	15	DPP4	FALSE	DPP4
364.6765	0.001005	15	VIM	FALSE	VIM
624.8304	9.29E-04	15	ENG	FALSE	ENG
183.0266	9.27E-04	15	RETN	FALSE	RETN
742.4567	9.36E-04	15	CANX	FALSE	CANX
70.58684	9.36E-04	13	CRK	TRUE	CRK
833.3732	9.00E-04	15	F3	FALSE	F3
212.6247	9.33E-04	15	DCN	FALSE	DCN
305.6335	9.43E-04	15	UBE2I	FALSE	UBE2I
3.315262	8.79E-04	5	PDPK1	TRUE	PDPK1
15.97469	9.84E-04	16	ZEB1	FALSE	ZEB1
81.06371	9.76E-04	13	KLF4	TRUE	KLF4
1802.845	9.07E-04	15	GPX4	FALSE	GPX4
1564.349	9.27E-04	14	FASN	FALSE	FASN
134.2264	8.84E-04	14	GHRL	TRUE	GHRL
16.89219	9.29E-04	9	PIK3CG	TRUE	PIK3CG
107.9783	9.32E-04	7	NR4A1	TRUE	NR4A1
836.1352	9.69E-04	14	CCT3	FALSE	CCT3
113.9817	9.48E-04	14	TLN1	TRUE	TLN1
405.6783	9.14E-04	14	ADRB2	TRUE	ADRB2
49.92462	8.45E-04	4	PON1	TRUE	PON1
192.7723	9.87E-04	14	HSPB1	TRUE	HSPB1
13.83973	8.37E-04	6	SPARC	TRUE	SPARC
254.8752	9.35E-04	14	YWHAE	TRUE	YWHAE
1134.914	9.48E-04	13	RAC1	FALSE	RAC1
306.6448	9.56E-04	13	NOS2	TRUE	NOS2
625.0222	9.42E-04	13	CALM1	TRUE	CALM1
68.57003	9.17E-04	18	SELP	FALSE	SELP
378.2917	9.63E-04	13	PDIA3	TRUE	PDIA3
152.2096	9.53E-04	13	ELAVL1	TRUE	ELAVL1
228.3244	8.75E-04	9	PTH	TRUE	PTH

197.592	8.72E-04	9	PDHB	TRUE	PDHB
22.09452	8.35E-04	5	HK1	TRUE	HK1
26.32395	8.24E-04	4	APCS	TRUE	APCS
0	7.72E-04	1	APLP2	TRUE	APLP2
0	7.74E-04	2	APOC1	TRUE	APOC1
103.0806	7.78E-04	2	HSD17B10	TRUE	HSD17B10
915.9246	9.51E-04	13	ISG15	FALSE	ISG15
976.155	9.43E-04	12	TUBA1A	FALSE	TUBA1A
265.2445	9.75E-04	12	NEDD4	TRUE	NEDD4
409.0948	9.31E-04	12	GRK2	TRUE	GRK2
452.7773	8.12E-04	6	UBQLN1	TRUE	UBQLN1
69.154	8.84E-04	5	CRYAB	TRUE	CRYAB
138.4974	9.33E-04	12	YWHAQ	TRUE	YWHAQ
6.161942	8.76E-04	6	AREG	TRUE	AREG
3.708534	8.64E-04	6	NRG1	TRUE	NRG1
526.6855	9.43E-04	12	HSPD1	TRUE	HSPD1
1.487049	7.65E-04	2	BHLHE40	TRUE	BHLHE40
14.48271	8.35E-04	5	NRIP1	TRUE	NRIP1
423.1628	9.61E-04	12	NCL	TRUE	NCL
1100.459	8.80E-04	11	ACTA1	FALSE	ACTA1
19.15277	8.26E-04	9	CCR10	TRUE	CCR10
28.91379	8.21E-04	3	KSR1	TRUE	KSR1
26.79745	7.42E-04	3	PTH1R	TRUE	PTH1R
12.6555	8.18E-04	4	ATP1A1	TRUE	ATP1A1
12.6555	8.18E-04	4	ATP1A3	TRUE	ATP1A3
0	6.61E-04	1	ATP2A1	TRUE	ATP2A1
429.2173	7.95E-04	10	RYR2	TRUE	RYR2
0	6.77E-04	2	SLC8A1	TRUE	SLC8A1
67.37982	8.34E-04	10	BAG3	TRUE	BAG3
7.09462	8.84E-04	6	HSPA1A	TRUE	HSPA1A
17.95741	8.36E-04	5	HSPA2	TRUE	HSPA2
8.282413	7.28E-04	2	BAG6	TRUE	BAG6
25.42436	7.63E-04	2	SGTA	TRUE	SGTA

1.929624	6.72E-04	2	BMP1	TRUE	BMP1
52.92816	6.99E-04	3	KCNK3	TRUE	KCNK3
24.12734	8.60E-04	4	BNIP3L	TRUE	BNIP3L
1803.955	8.99E-04	11	ATP2A2	FALSE	ATP2A2
27.83813	8.03E-04	5	GABARAPL1	TRUE	GABARAPL1
19.95554	8.16E-04	5	CUL3	TRUE	CUL3
47.6586	7.79E-04	3	FBXO2	TRUE	FBXO2
0	7.10E-04	1	HIVEP2	TRUE	HIVEP2
286.0669	9.46E-04	11	P4HB	TRUE	P4HB
0	7.12E-04	2	FBXO6	TRUE	FBXO6
304.8398	9.17E-04	11	CCT8	TRUE	CCT8
0	7.23E-04	2	C1QA	TRUE	C1QA
56.73481	7.73E-04	4	C1QB	TRUE	C1QB
43.76618	8.45E-04	4	PTX3	TRUE	PTX3
97.39746	8.64E-04	7	DLG1	TRUE	DLG1
0	8.03E-04	5	PPIB	TRUE	PPIB
302.3385	9.08E-04	11	NOS1	TRUE	NOS1
1390.435	8.88E-04	11	GSTP1	FALSE	GSTP1
14.97189	8.06E-04	4	HLA-E	TRUE	HLA-E
26.8385	7.67E-04	5	TAP1	TRUE	TAP1
652.5855	9.46E-04	10	PPP1CA	TRUE	PPP1CA
263.5768	9.03E-04	10	SLC9A1	TRUE	SLC9A1
38.89575	9.49E-04	10	CD81	TRUE	CD81
26.18365	7.74E-04	5	TOMM20	TRUE	TOMM20
837.6001	7.58E-04	6	RAB7A	FALSE	RAB7A
60.80837	7.34E-04	4	CAT	TRUE	CAT
17.77082	7.11E-04	2	PEX5	TRUE	PEX5
4.727977	7.23E-04	3	SOD3	TRUE	SOD3
268.6985	9.62E-04	10	CD38	TRUE	CD38
0	7.71E-04	1	SNAP23	TRUE	SNAP23
257.5771	7.86E-04	4	KCNA5	TRUE	KCNA5
292.4261	8.70E-04	9	TXNRD1	TRUE	TXNRD1
26.61586	8.92E-04	17	CX3CL1	FALSE	CX3CL1

1524.195	8.90E-04	10	PRKCE	FALSE	PRKCE
150.9169	9.71E-04	10	IGFBP3	TRUE	IGFBP3
31.79559	9.26E-04	19	CCL4	FALSE	CCL4
705.9489	9.11E-04	9	ACLY	TRUE	ACLY
51.18364	9.32E-04	20	CXCL2	FALSE	CXCL2
111.9277	8.87E-04	9	GSN	TRUE	GSN
152.7938	8.83E-04	9	H2BC4	TRUE	H2BC4
45.2501	8.71E-04	9	CCND2	TRUE	CCND2
170.1686	9.19E-04	9	NOX1	TRUE	NOX1
18.7831	8.74E-04	7	IGFBP7	TRUE	IGFBP7
22.68471	8.80E-04	8	E2F4	TRUE	E2F4
114.9512	8.73E-04	8	PCNA	TRUE	PCNA
876.1451	9.05E-04	9	ELN	FALSE	ELN
279.7483	8.41E-04	6	GNB2	TRUE	GNB2
0	7.44E-04	3	CTTNBP2	TRUE	CTTNBP2
2.659632	7.50E-04	3	NOP56	TRUE	NOP56
112.1634	7.78E-04	7	CDC20	TRUE	CDC20
0	7.92E-04	2	FABP5	TRUE	FABP5
12.15945	7.85E-04	3	OLR1	TRUE	OLR1
197.585	8.98E-04	9	ARNTL	TRUE	ARNTL
3.543955	7.97E-04	3	LGALS9	TRUE	LGALS9
66.51707	9.07E-04	13	IFNGR1	TRUE	IFNGR1
46.44739	9.14E-04	13	PLAT	TRUE	PLAT
161.7136	8.95E-04	9	OPTN	TRUE	OPTN
818.3796	7.21E-04	4	MAPRE1	FALSE	MAPRE1
876.6582	8.93E-04	9	XPO1	FALSE	XPO1
1373.459	8.18E-04	7	CDC5L	FALSE	CDC5L
0	6.56E-04	2	LSM3	TRUE	LSM3
0	6.56E-04	2	SCNM1	TRUE	SCNM1
0	6.16E-04	1	SRPK2	TRUE	SRPK2
1142.653	8.51E-04	8	SNW1	FALSE	SNW1
2.122083	7.83E-04	3	RPL17	TRUE	RPL17
0	7.39E-04	2	ENSA	TRUE	ENSA

742.9392	9.53E-04	8	EIF2AK3	FALSE	EIF2AK3
0	5.76E-04	1	CIAO1	TRUE	CIAO1
802	7.50E-04	5	RSAD2	FALSE	RSAD2
0	7.86E-04	1	CIRBP	TRUE	CIRBP
102.1224	7.30E-04	2	CKB	TRUE	CKB
15.92904	8.40E-04	5	COL18A1	TRUE	COL18A1
27.72051	7.47E-04	3	COX5B	TRUE	COX5B
1000.813	8.55E-04	6	PTGS1	FALSE	PTGS1
35.66023	7.63E-04	3	UQCRFS1	TRUE	UQCRFS1
1.298939	7.52E-04	3	CPT1B	TRUE	CPT1B
169.0901	8.61E-04	6	PDK4	TRUE	PDK4
117.545	8.92E-04	8	ALDOA	TRUE	ALDOA
11.45482	8.09E-04	4	PAPPA	TRUE	PAPPA
0	6.59E-04	1	GK	TRUE	GK
13.40594	7.86E-04	5	PDHA1	TRUE	PDHA1
30.93052	8.81E-04	8	IL22RA1	TRUE	IL22RA1
41.46045	8.87E-04	10	IL10RB	TRUE	IL10RB
8.866362	9.18E-04	10	IL6ST	TRUE	IL6ST
54.40346	9.29E-04	14	CSF3R	TRUE	CSF3R
388.1697	8.10E-04	5	PSMB1	TRUE	PSMB1
47.12448	9.46E-04	13	PRL	TRUE	PRL
0	8.15E-04	1	SOX17	TRUE	SOX17
0	8.77E-04	3	SIAH1	TRUE	SIAH1
802	8.33E-04	3	TLE4	FALSE	TLE4
0	8.76E-04	3	FHL2	TRUE	FHL2
50.90584	8.56E-04	4	CUL7	TRUE	CUL7
81.24784	8.45E-04	7	MX1	TRUE	MX1
0	8.00E-04	3	VCAN	TRUE	VCAN
51.25445	8.08E-04	5	DNM1L	TRUE	DNM1L
0	7.60E-04	2	DNMT3L	TRUE	DNMT3L
41.90738	9.27E-04	8	DUSP1	TRUE	DUSP1
36.73394	8.62E-04	6	ZFP36	TRUE	ZFP36
0	8.22E-04	2	DUSP6	TRUE	DUSP6

24.70172	9.13E-04	12	GHR	TRUE	GHR
33.39953	9.32E-04	9	FGF1	TRUE	FGF1
260.6633	9.20E-04	8	HK2	TRUE	HK2
669.5471	9.07E-04	7	MSTN	TRUE	MSTN
330.9856	9.17E-04	6	RAB5A	TRUE	RAB5A
17.84519	7.87E-04	5	EGR2	TRUE	EGR2
88.82174	8.73E-04	7	NFATC1	TRUE	NFATC1
59.21373	8.83E-04	7	JUNB	TRUE	JUNB
0.666667	6.91E-04	2	EIF2A	TRUE	EIF2A
84.99353	7.95E-04	3	EIF2AK4	TRUE	EIF2AK4
13.30803	8.71E-04	5	G3BP1	TRUE	G3BP1
0	7.53E-04	1	ELMSAN1	TRUE	ELMSAN1
0	6.64E-04	1	FBLN5	TRUE	FBLN5
511.4012	8.06E-04	10	PRDX6	TRUE	PRDX6
0	6.61E-04	1	F2RL1	TRUE	F2RL1
20.45274	6.83E-04	2	HADHA	TRUE	HADHA
1274.052	8.89E-04	5	LMNA	FALSE	LMNA
62.58618	8.68E-04	6	TRIM25	TRUE	TRIM25
0	7.47E-04	3	GATM	TRUE	GATM
0	7.90E-04	2	GBP2	TRUE	GBP2
459.0456	7.34E-04	5	NPR1	TRUE	NPR1
4.520117	5.92E-04	2	GCH1	TRUE	GCH1
350.8783	7.28E-04	2	SPR	TRUE	SPR
5.133451	8.03E-04	6	GCLC	TRUE	GCLC
217.3042	8.88E-04	5	CEBPD	TRUE	CEBPD
0	8.42E-04	1	GDF15	TRUE	GDF15
0	8.34E-04	4	MC4R	TRUE	MC4R
0	7.02E-04	7	GSTM2	TRUE	GSTM2
0	7.02E-04	7	GSTM1	TRUE	GSTM1
129.0257	7.71E-04	8	GSTA1	TRUE	GSTA1
0	7.02E-04	7	GSTM4	TRUE	GSTM4
0	7.02E-04	7	GSTM3	TRUE	GSTM3
13.01016	7.50E-04	2	RHOU	TRUE	RHOU

0.326923	7.84E-04	2	PTPRA	TRUE	PTPRA
42.82758	7.78E-04	3	PEBP1	TRUE	PEBP1
140.6946	8.14E-04	6	PPP3R1	TRUE	PPP3R1
6.86125	8.06E-04	4	MAP2K7	TRUE	MAP2K7
0	5.07E-04	1	GYG1	TRUE	GYG1
809.241	6.37E-04	3	PYGB	FALSE	PYGB
34.11918	8.75E-04	8	HDAC5	TRUE	HDAC5
5.951146	8.66E-04	8	YWHAG	TRUE	YWHAG
17.70662	7.91E-04	4	PFKFB3	TRUE	PFKFB3
0	1	1	ID1	FALSE	ID1
0	1	1	ID3	FALSE	ID3
0.25	7.82E-04	4	IFNL3	TRUE	IFNL3
14.09495	7.58E-04	2	PPM1B	TRUE	PPM1B
7.948357	6.67E-04	2	KCNJ8	TRUE	KCNJ8
0	6.55E-04	1	TMPO	TRUE	TMPO
28.29724	7.65E-04	3	PAK2	TRUE	PAK2
11.843	8.16E-04	3	PLA2G2A	TRUE	PLA2G2A
0	5.59E-04	1	PDE4DIP	TRUE	PDE4DIP
0	7.67E-04	1	MVP	TRUE	MVP
0	7.70E-04	1	NKIRAS1	TRUE	NKIRAS1
49.79821	7.75E-04	4	TPM4	TRUE	TPM4
0	6.57E-04	1	NXF1	TRUE	NXF1
0	6.56E-04	1	PDLIM5	TRUE	PDLIM5
0	6.92E-04	1	UCN2	TRUE	UCN2
0	7.82E-04	1	RXRG	TRUE	RXRG
6.220851	7.41E-04	3	PPP1R1A	TRUE	PPP1R1A
3.022222	6.46E-04	3	UBQLN4	TRUE	UBQLN4
103.1711	7.43E-04	4	PSMB5	TRUE	PSMB5
0	6.37E-04	1	PTGIR	TRUE	PTGIR
0	1	1	RAB1B	FALSE	RAB1B
0	1	1	TMED2	FALSE	TMED2
0	5.81E-04	1	RAB7B	TRUE	RAB7B
0	6.87E-04	1	RALBP1	TRUE	RALBP1

0	8.42E-04	1	RGCC	TRUE	RGCC
0	5.88E-04	2	RXFP4	TRUE	RXFP4
0	0.5	2	TCTN2	FALSE	TCTN2
0	0.5	2	TMEM17	FALSE	TMEM17
0	0.5	2	TMEM216	FALSE	TMEM216
0	6.24E-04	1	TLE5	TRUE	TLE5
0	7.08E-04	1	TNS2	TRUE	TNS2
0.4	6.85E-04	2	TUBB1	TRUE	TUBB1

Table 7 Degree unDir, Betweenness unDir, and Closeness unDir calculated by CentiScaPe 2.2 for the Traditional Chinese Medicine-Component-Protein Interaction Network.

Betweenness unDir	Closeness unDir	Degree unDir	displayName	name
45339.33	8.95E-04	52	ACTB	entrez.60
27153.98	8.12E-04	41	HDAC1	entrez.3065
33573.1	8.77E-04	35	HSPA8	entrez.3312
25731.58	8.08E-04	33	ITGB1	entrez.3688
17251.1	8.45E-04	29	TUBA1A	entrez.7846
20198.33	8.43E-04	29	ENO1	entrez.2023
12887.84	7.87E-04	22	ADRB2	entrez.154
11479.59	7.52E-04	19	CCND1	entrez.595
4327.09	7.15E-04	18	PDHA1	entrez.5160
6884.08	7.22E-04	18	PPARG	entrez.5468
6381.527	7.37E-04	18	NCL	entrez.4691
7050.644	7.48E-04	17	HSPB1	entrez.3315
8904.869	6.93E-04	16	GRK2	entrez.156
6430.593	7.43E-04	16	RAC1	entrez.5879
5232.961	7.15E-04	15	PPM1B	entrez.5495

5192.073	6.16E-04	15	PFKM	entrez.5213
4593.125	7.42E-04	15	SOD1	entrez.6647
4973.137	7.01E-04	15	ICAM1	entrez.3383
6921.838	7.03E-04	14	CANX	entrez.821
4508.497	7.24E-04	13	SIRT1	entrez.23411
4718.386	7.70E-04	12	VIM	entrez.7431
3821.543	7.26E-04	12	HDAC5	entrez.10014
5934.211	7.19E-04	12	PPP2CA	entrez.5515
6189.562	7.20E-04	12	CAV1	entrez.857
4176.934	6.92E-04	11	MAPK14	entrez.1432
2018.602	7.04E-04	11	LDHB	entrez.3945
3860.664	6.87E-04	11	ATP1A1	entrez.476
6206.096	7.20E-04	11	APP	entrez.351
3203.52	7.67E-04	11	CRK	entrez.1398
3913.261	7.56E-04	11	HSPA9	entrez.3313
4864.288	7.50E-04	10	ELAVL1	entrez.1994
5016.507	6.97E-04	10	GSK3B	entrez.2932
5068.521	6.64E-04	10	ALB	entrez.213
3184.518	7.60E-04	10	VCAM1	entrez.7412
1986.868	6.89E-04	9	TNFRSF1A	entrez.7132
1197.597	6.70E-04	9	TUBA1C	entrez.84790
996.645	6.75E-04	9	ALDOA	entrez.226
2495.392	7.10E-04	9	VCL	entrez.7414
2847.637	6.47E-04	9	TRIM25	entrez.7706
3462.728	8.01E-04	9	HSPD1	entrez.3329
2885.335	7.10E-04	9	HSPA1A	entrez.3303
3958.786	6.12E-04	8	AGT	entrez.183
1420.615	6.44E-04	8	SMAD4	entrez.4089
1004.461	6.96E-04	8	MSN	entrez.4478
2367.848	6.06E-04	8	F2RL1	entrez.2150
2356.558	6.73E-04	8	RAB5A	entrez.5868
1607.213	6.66E-04	7	MAPRE1	entrez.22919
2808.654	6.15E-04	7	AGTR1	entrez.185

2206.984	7.49E-04	7	TP53	entrez.7157
1712.662	7.31E-04	7	PDIA3	entrez.2923
1590.518	6.57E-04	7	HDAC4	entrez.9759
2416.383	7.10E-04	7	HIF1A	entrez.3091
2534.908	6.99E-04	7	YWHAZ	entrez.7534
1520.199	6.75E-04	7	CALR	entrez.811
1372.307	6.05E-04	6	TPM4	entrez.7171
558.2379	6.35E-04	6	DLAT	entrez.1737
390.3876	6.39E-04	6	DNM1L	entrez.10059
963.6206	6.58E-04	6	P4HB	entrez.5034
695.2673	6.12E-04	6	PRKAR2B	entrez.5577
483.7332	6.23E-04	6	PSMB1	entrez.5689
1043.361	6.87E-04	6	STAT5A	entrez.6776
1859.962	6.11E-04	6	BMPR1B	entrez.658
1033.751	6.43E-04	6	CAT	entrez.847
1493.517	7.51E-04	6	GAPDH	entrez.2597
416.1267	6.63E-04	6	ITGB3	entrez.3690
1418.395	6.57E-04	6	YWHAQ	entrez.10971
3132.28	7.60E-04	6	MYC	entrez.4609
771.1366	6.86E-04	5	KCNA5	entrez.3741
1516.591	6.62E-04	5	GNA12	entrez.2768
1583.18	6.58E-04	5	TLR4	entrez.7099
2872.025	6.86E-04	5	JAK2	entrez.3717
1963.845	7.00E-04	5	GSN	entrez.2934
921.7016	6.96E-04	5	ACTG1	entrez.71
957.0193	7.00E-04	5	LRRK2	entrez.120892
1876.493	6.22E-04	5	TNF	entrez.7124
1100.284	6.21E-04	5	ACLY	entrez.47
1036.13	6.33E-04	5	SMAD1	entrez.4086
1305.907	6.82E-04	5	BHLHE40	entrez.8553
1174.336	6.35E-04	5	PEBP1	entrez.5037
2722.364	6.84E-04	5	ZFP36	entrez.7538
1951.947	6.95E-04	5	AKT1	entrez.207

529.8694	6.90E-04	5	PCK1	entrez.5105
666.9054	6.51E-04	5	SLC9A1	entrez.6548
1393.145	5.97E-04	5	NR4A1	entrez.3164
994.0874	6.47E-04	5	HMGB1	entrez.3146
448.8367	6.42E-04	5	HADHA	entrez.3030
735.6071	5.91E-04	5	BMPR2	entrez.659
1937.704	6.59E-04	5	PLA2G2A	entrez.5320
2420.438	6.35E-04	5	IGFBP3	entrez.3486
1008.114	6.26E-04	5	IFNGR1	entrez.3459
2606.688	7.12E-04	5	NFKBIA	entrez.4792
1551.379	7.34E-04	5	CTNNB1	entrez.1499
5176.18	6.76E-04	5	HSPA2	entrez.3306
1028.058	6.08E-04	5	RAB1B	entrez.81876
1178.979	7.26E-04	5	CD4	entrez.920
1067.901	6.98E-04	5	NOS2	entrez.4843
593.8337	6.25E-04	5	PPARGC1A	entrez.10891
2718.773	6.13E-04	5	VCAN	entrez.1462
1048.614	6.31E-04	4	TLE4	entrez.7091
1329.721	6.05E-04	4	VAPA	entrez.9218
1531.744	6.56E-04	4	RALBP1	entrez.10928
547.0162	6.31E-04	4	TFRC	entrez.7037
1227.393	6.67E-04	4	EGFR	entrez.1956
1272.687	6.29E-04	4	PDE4DIP	entrez.9659
993.7721	6.09E-04	4	PAK2	entrez.5062
722.2767	6.08E-04	4	RPL17	entrez.6139
958.446	6.57E-04	4	PDPK1	entrez.5170
1982.816	5.96E-04	4	RTN1	entrez.6252
496.9936	6.37E-04	4	HK2	entrez.3099
1392.746	6.87E-04	4	PPP1CA	entrez.5499
1279.188	7.33E-04	4	SHC1	entrez.6464
1346.16	6.56E-04	4	EZR	entrez.7430
1686.368	6.29E-04	4	TMEM17	entrez.200728
1080.637	6.42E-04	4	YWHAB	entrez.7529

474.773	6.53E-04	4	YWHAG	entrez.7532
372.8617	6.58E-04	4	FASN	entrez.2194
744.2102	7.14E-04	4	CCND2	entrez.894
388.4473	5.97E-04	4	POMC	entrez.5443
433.8586	6.05E-04	4	ARNTL	entrez.406
999.8614	6.21E-04	4	FHL2	entrez.2274
1291.384	6.37E-04	4	NFE2L2	entrez.4780
1358.19	6.46E-04	4	PDLIM5	entrez.10611
1244.94	6.57E-04	4	PTEN	entrez.5728
395.8317	6.36E-04	4	KDR	entrez.3791
408.9956	6.11E-04	4	CAMK2D	entrez.817
499.9194	6.63E-04	4	NEDD4	entrez.4734
742.9134	6.14E-04	3	FBXO6	entrez.26270
637.3634	6.25E-04	3	ACTA1	entrez.58
2910.043	6.63E-04	3	SMAD9	entrez.4093
668.786	6.60E-04	3	ACACA	entrez.31
2044.937	6.68E-04	3	ISG15	entrez.9636
664.4766	6.32E-04	3	SIAH1	entrez.6477
410.5183	6.35E-04	3	CCT3	entrez.7203
507.0711	6.31E-04	3	SQSTM1	entrez.8878
1160.983	6.10E-04	3	TCTN2	entrez.79867
1852.383	6.39E-04	3	SGTA	entrez.6449
901.2485	6.39E-04	3	MVP	entrez.9961
1545.249	6.29E-04	3	STAT3	entrez.6774
881.5436	6.68E-04	3	ARRB1	entrez.408
1000.269	6.56E-04	3	CDC5L	entrez.988
1692.245	6.54E-04	3	SRPK2	entrez.6733
411.7626	6.37E-04	3	NFATC1	entrez.4772
994.0602	6.45E-04	3	HSPA5	entrez.3309
407.5826	6.36E-04	3	NPM1	entrez.4869
593.8842	6.59E-04	3	NOS3	entrez.4846
672.6536	5.99E-04	2	LGALS9	entrez.3965
822.7112	6.01E-04	2	FBXO2	entrez.26232

446.6684	6.45E-04	2	DDX5	entrez.1655
1092.129	6.59E-04	2	ACE	entrez.1636
585.2864	6.18E-04	2	GSTM3	entrez.2947
457.3644	6.47E-04	2	CCR10	entrez.2826
719.3956	6.20E-04	2	CXCL2	entrez.2920
646.1995	5.98E-04	2	TNS2	entrez.23371
437.4969	6.43E-04	2	H2BC7	entrez.8343
555.154	6.53E-04	2	CUL3	entrez.8452
514.709	6.44E-04	2	SLC25A11	entrez.8402
448.3899	6.11E-04	2	MIDEAS	entrez.91748
896.7477	6.19E-04	2	PPIB	entrez.5479
408.0259	5.99E-04	2	VCP	entrez.7415
1029.816	6.45E-04	2	CCT8	entrez.10694
636.0901	6.15E-04	2	RXFP3	entrez.51289
520.1412	6.00E-04	2	ENG	entrez.2022
648.2188	6.00E-04	2	SYK	entrez.6850
474.4258	6.02E-04	2	NFKB1	entrez.4790
1250.922	6.37E-04	2	BAG6	entrez.7917
2330.67	6.37E-04	2	PTGIR	entrez.5739
440.2259	6.46E-04	2	CCN2	entrez.1490

Table 8 Top 30 drug candidates and their molecular docking results with key proteins

Durg	STAT3	AKT1	IL6	EGFR	TP53
Sunitinib	-10.3	-8.9	-9	-11.8	-8.4
Nintedanib	-12.8	-11.6	-11	-16.5	-11
Midostaurin	-9.8	-9.4	-9.8	-12.3	-10.7
Bosutinib	-11.5	-10.9	-9.7	-14.1	-9.7

Dasatinib	/	-8.5	-8.8	-12.6	-8.2
Sorafenib	-11.2	-10.1	-10.5	-13.5	-9.9
Crizotinib	-9.5	-8.8	-8.9	-11.5	-8.3
Ruxolitinib	-7.6	-7.6	-8.3	-9.2	-6.9
Vandetanib	-9.3	-8.1	-8.2	-11	-7.8
Neratinib	-10	-8.9	-9.7	-13.1	-9.2
Pazopanib	-10.3	-9.2	-9.4	-12.7	-9.3
Axitinib	-9.8	-8.6	-9.1	-13.1	-8.8
Erlotinib	-8.2	-7.9	-8.5	-9.3	-7.3
Tamoxifen	-9.5	-10	-9.3	-12.2	-9.3
Astemizole	-10.3	-9.1	-9.5	-12	-8.6
Chlorpromazine	-6.6	-6.7	-7.1	-8	-6.1
Clotrimazole	-10.4	-10.5	-11.3	-12.9	-9.9
Amiodarone	-8.4	-8.8	-8.5	-10.3	-8
Miconazole	-10	-8.7	-9.4	-11.6	-8.7
Imatinib	-11.3	-9.5	-9.8	-14.4	-10.2
Nilotinib	-10.1	-9.5	-10.2	-13.6	-9.7
Econazole	-8.2	-7.9	-8	-9.8	-7.6
Gefitinib	-8.5	-7.1	-7.4	-10	-7.1
Tioguanine	-5.2	-5	-5	-5.5	-3.9
Metformin	-5.8	-4.6	-4.6	-4.9	-3.4
Raloxifene	-10.6	-9.8	-9.9	-14.4	-9.3
Fluphenazine	-9.7	-8.6	-8.9	-11	-8.1
Captopril	-6.6	-6.3	-6.6	-8.3	-5.6
Haloperidol	-9	-8.4	-8.4	-11.2	-8
Diethylstilbestrol	-7.9	-7.6	-8.2	-9.6	-7.2

Table 9 Key Panax ginseng components and their molecular docking results with key proteins

	EGFR	AKT1	PTGS2	NFKB1	TNF	PPARG

Gomisin B	-10.9	-8.3	-13	-6.1	-11.5	-11.2
Deoxyharringtonine	-10.5	-8.4	-9.9	-6	-10.7	-10.6
Ginsenoside Rh4	-16.6	-10.7	-16.1	-7.9	-15.1	-13.6
Girinimbin	-10.8	-9	-12.1	-6.3	-10	-10.2
ginsenoside Rg5	-15	-10.4	-13.8	-7.6	-15	-13.7
arachidonate	-10.5	-7.9	-9.8	-5.5	-8.6	-9

Table 10 The protein used for molecular docking and its Entry ID

Protein	Entry ID
AKT1	1H10
EGFR	1XKK
IL-6	1ALU
TP53	1C26
PPARG	1ZGY
NFKB1	7RG5
PTGS2	5F19
STAT3	6NJS
